# Loss of OsEAF6, a Subunit of the Histone Acetyltransferase Complex, Causes Hybrid Breakdown in Intersubspecific Rice Crosses

**DOI:** 10.3389/fpls.2022.866404

**Published:** 2022-03-08

**Authors:** Takahiko Kubo, Atsushi Yoshimura, Nori Kurata

**Affiliations:** ^1^Plant Genetics Laboratory, National Institute of Genetics, Mishima, Japan; ^2^Plant Breeding Laboratory, Faculty of Agriculture, Kyushu University, Fukuoka, Japan

**Keywords:** hybrid breakdown, histone acetyltransferase, rice, duplicate recessive gene, speciation

## Abstract

Gene duplication plays an important role in genetic diversification, adaptive evolution, and speciation. Understanding the mechanisms and effects of postzygotic isolation genes is important for further studies of speciation and crop breeding. The duplicate recessive genes *hwe1* and *hwe2* cause hybrid breakdown, characterized by poor vegetative growth and reproductive dysgenesis in intersubspecific crosses between *Oryza sativa* ssp. *indica* and *japonica*. Using a map-based cloning strategy, we found that *HWE1* and *HWE2* encode the Esa1-associated factor 6 (EAF6) protein, a component of histone acetyltransferase complexes. The *indica hwe1* and *japonica hwe2* alleles lacked functional *EAF6*, demonstrating that the double recessive homozygote causes hybrid breakdown. Morphological and physiological observations showed that weak plants with double recessive homozygotes had serious morphological defects with a wide range of effects on development and organs, leading to leaves with reduced chlorophyll content, flower and pistil malformation, and anomalies of gametogenesis. These findings suggest that EAF6 plays a pivotal role in the transcriptional regulation of essential genes during the vegetative and reproductive development of rice.

## Introduction

In eukaryotic cells, histone acetylation regulates the chromatin structure, affecting gene transcription, DNA replication, and DNA damage repair. Nucleosome acetyltransferase of histone 4 (NuA4), a histone acetyltransferase (HAT) complex, is composed of multiple proteins and preferentially acetylates histones H4 and H2A on the nucleosome. The components of NuA4 are highly conserved in yeast and human (Doyon et al., 2004). Yeast NuA4 consists of 13 subunits, with two independent NuA4 sub-complexes, namely, piccolo-NuA4, composed of Esa1, Epl1, Yng2, and Eaf6, and the TINTIN triad of Eaf5/7/3 (Wang X. et al., 2018). Piccolo-NuA4, which is thought to also exist alone, contains the catalytic subunit protein essential Sas2-related acetyltransferase-1 (Esa1) (Ohba et al., 1999; Boudreault et al., 2003). Esa1 alone can acetylate free histones but cannot acetylate nucleosomal histones (Doyon et al., 2004). This protein also plays a crucial role in cell cycle progression and DNA double-strand break repair (Clarke et al., 1999; Bird et al., 2002) and is essential for yeast cell viability (Doyon et al., 2004). Another component, Esa1-associated factor 6 (EAF6, known as MEAF6 in mammals), interacts with Piccolo-NuA4 through Yng2 in yeast (Mitchell et al., 2008). Unlike the catalytic subunit Esa1, the yeast *eaf6*Δ mutant is viable without detectable changes, indicating that the NuA4 subunit is not essential for yeast cellular processes (Lafon et al., 2007). However, a study in human showed that a fusion protein of MEAF6 with PHD finger protein 1 generated by chromosomal translocation caused endometrial stromal tumors in human (Panagopoulos et al., 2012). These findings suggest that EAF6 (MEAF6) is important in cell proliferation. Yeast EAF6 is also a component of another HAT complex, nucleosome acetyltransferase of histone 3, which acetylates histone H3. This HAT complex was reported to be involved in transcriptional activation and cell cycle regulation (Lafon et al., 2007). Compared with the number of studies performed to characterize NuA4 components in yeast and mammals, studies in plants are limited. Two components of the NuA4 complex, namely, EAF1 and YAF9, were found to regulate flowering *via* histone H4 acetylation in *Arabidopsis* (Zacharaki et al., 2012; Bieluszewski et al., 2015). Double mutations in HAM1 and HAM2 (*Arabidopsis* ESA1 homologs) induced lethality in diploid plants and haploid gametophytes, suggesting a function in histone acetylation during mitotic cell division of gametogenesis in *Arabidopsis* (Latrasse et al., 2008). The plant EAF6 protein is an uncharacterized potential subunit of plant NuA4; less is known about its biological functions in plant development and growth.

Hybrid breakdown is defined as deleterious characteristics, such as sterility and non-viability, occurring only after F_2_ generations of crosses between distantly related species. Similar to other hybrid incompatibility mechanisms, hybrid breakdown contributes to speciation by restricting gene flow between diverging taxa. Although such phenomena are widely observed in numerous animal and plant species (Stebbins, 1958), few hybrid breakdown genes have been identified and characterized at the molecular level. Seminal studies in plants demonstrated that autoimmune responses involving nucleotide-binding site-leucine-rich repeat genes can cause hybrid weakness and breakdown (Bomblies et al., 2007; Alcazar et al., 2010). A further systematic study using a large diallel cross containing more than 6,400 cross combinations in *Arabidopsis* revealed that one hybrid necrosis gene, *Dangerous Mix 2* (*DM2*), plays a central role in the epistatic network involving numerous independent loci related to hybrid necrosis (Chae et al., 2014). Such autoimmune systems cause hybrid incompatibility, including the hybrid breakdown in other plant species (Hannah et al., 2007; Jeuken et al., 2009; Yamamoto et al., 2010; Chen et al., 2014). Previous studies demonstrated that defense systems against biotic stress, including programmed cell death and nucleotide-binding site-leucine-rich repeats, are major and common causes of hybrid weakness and hybrid breakdown in plant species. Although progress has been made recently in identifying the genes involved in hybrid incompatibility, the molecular basis of hybrid breakdown other than the autoimmune response is poorly understood in plants. In many cases, the causal genes remain unknown. Whether other physiological mechanisms underlie hybrid weakness and how different genes contribute to genetic diversification and speciation are also unclear.

Our previous study demonstrated hybrid breakdown characterized by weak growth and complete sterility between *Oryza sativa* ssp. *indica* and *japonica* (2*n* = 24). Genetic analysis has revealed that this weakness is caused by double recessive genes, *hwe1* and *hwe2*, which are localized on rice chromosomes 1 and 12, respectively (Kubo and Yoshimura, 2002). Although the phenomenon and basic genetics of this hybrid breakdown were characterized more than a decade ago, the molecular mechanism is not well-understood. The specific objectives of this study were to isolate *hwe1* and *hwe2*, characterize the abnormal phenotype of the weak plant morphologically and physiologically, and identify the physiological function of the causal genes at the molecular level.

## Materials and Methods

### Plant Materials

The characterization of weak plants and high-resolution mapping was carried out using the backcross population derived from the population previously used for rough mapping of *hwe1* and *hwe2* (Kubo and Yoshimura, 2002). Additionally, we used two other *indica/japonica* populations, namely, BC_2_F_2_ derived from the Nipponbare/93-11 cross (Kubo et al., 2011) and a newly developed Nipponbare/IR8 F_2_ population.

### Characterization of Morphological and Physiological Traits

Normal and weak segregants (BC_3_F_6–7_, *n* = 10) were evaluated to determine their seed fertility, column length, and number. The chlorophyll content was examined using the last fully opened leaf blades from each genotype (*n* = 5) at the tillering stage. Seed fertility was evaluated as previously described (Kubo et al., 2016). To evaluate leaf cell viability related to the autoimmune response, leaves from normal and weak plants during vegetative development were collected and stained with trypan blue. For staining, the detached leaves were completely submerged in lactic acid-phenol-trypan blue solution (0.5 mg/ml trypan blue, 25% phenol, 25% lactic acid, and 25% glycerol) and microwaved for 1.0 min in a domestic microwave oven. The tissue was destained by placing the samples in staining solution without trypan blue and overnight incubation. The tissue was transferred to 50% ethanol and observed under a stereomicroscope.

### Map-Based Cloning

To perform high-resolution mapping of the *hwe1 and hwe2* loci, seedlings of the segregating populations (approximately 2,387 BC_3_F_5–6_ plants for *hwe2* and 383 BC_3_F_5–6_ individuals for *hwe1*) were genotyped using polymerase chain reaction (PCR)-based markers, and plants with recombination around the *hwe1* and *hwe2* loci were identified. PCR-based markers, insertion and deletion markers, and simple sequence repeat markers were identified using sequence polymorphism data for Nipponbare and 93-11 (MSU7.0)^1^. The primer sequences for the DNA markers are listed in Supplementary Table 1. For DNA marker genotyping, crude DNA extracts of seedling leaves were prepared using 0.25 M NaOH followed by neutralization with 0.1 M Tris–HCl. These DNA extracts were used in PCR with GoTaq polymerase (Promega, Madison, WI, United States) and the following cycling profile: 94°C for 2 min, followed by 30 cycles of 94°C for 20 s, 50–60°C for 20 s, and 72°C for 30 s.

### Gene Cloning and Rice Transformation

Gene cloning and allelic diversity analyses were carried out using PCR analysis of purified DNA from rice varieties and wild accessions prepared using the CTAB method (Murray and Thompson, 1980), followed by sequencing analysis on an ABI Prism 3130xl genetic analyzer (Applied Biosystems, Foster City, CA, United States). For the complementation test, the Asominori genomic DNA fragment (7,023 bp) containing the LOC_Os12g20310 gene with flanking 5′ (2,872 bp) and 3′ (1,487 bp) regions was amplified with KOD-Plus-Neo DNA polymerase (Toyobo, Osaka, Japan) using the primer G20310 (Supplementary Table 1). For another candidate, LOC_Os12g20324, the Asominori genomic DNA fragment (7,689 bp) was amplified by PCR using the primer G20324. The amplified fragments were cloned into the pBluescript SK cloning vector and subcloned into the pPZP2H-lac binary vector (Fuse et al., 2001). The cloned genomic fragments and empty vector were transformed into *HWE1* heterozygotes (*Hwe1/hwe1hwe2/hwe2*) *via Agrobacterium tumefaciens*-mediated transformation (Hiei et al., 1994; Nishimura et al., 2006). Complementation was examined based on the phenotype of the selfed progeny (T1 and T2) of the T0 transformant.

### Reverse Transcription-PCR Analysis

Total RNA from Asominori plant tissues (i.e., leaf, stem, root, young, and flowering panicles) and NILs were prepared using an RNeasy Plant Mini Kit (Qiagen, Hilden, Germany). cDNA was generated by reverse transcription of 2.0 μg of total RNA using the SuperScript III First-Strand Synthesis System (Life Technologies, Carlsbad, CA, United States). RT-PCR analysis was performed using two primer sets (i.e., eaf6sp and eaf6co) (Supplementary Table 1) to discriminate the products from the Nipponbare *HWE1* and *HWE2* loci. The cDNA of *OsAct1* (rice *Actin 1*) was amplified using the Act1 primer and used as a standard control. RT-PCR was performed in a Biometra thermocycler with the following cycling profile: 94°C for 2 min, followed by 32 cycles at 94°C for 20 s, 58°C for 20 s, and 72°C for 30 s.

### Histological Experiments

To observe the embryo sac, pre-flowering panicles were collected from normal and weak plants, fixed, and stored in FAA solution (45% ethanol, 5% formalin, and 5% acetic acid). After fixation, the samples were embedded in paraffin (Paraplast Plus; McCormick Scientific, St. Louis, MO, United States), sectioned, and stained with hematoxylin. To observe the morphology of the mature pollen grains, ethanol-fixed pollen grains were stained with 1.0% iodine-potassium iodide (I_2_-KI) and observed under a microscope. Male gametogenesis was analyzed using young panicles collected from normal and weak plants at different developmental stages. Panicles were fixed and stored in FAA solution. After fixation, the micropores were extracted from the anther using forceps, and released micropores were stained with hematoxylin solution as described by Kindiger and Beckett (1985).

### Histochemical Analysis of Beta-Glucuronidase Expression

An Asominori genome fragment containing 2,872 bp of the upstream region of *OsEAF6* (*LOC_Os12g20310*) was amplified using the primer set G20310pro (Supplementary Table 1) and cloned into the *Kpn* I-*Spe* I site of the binary vector pBGH2 (Ito and Kurata, 2008) to drive beta-glucuronidase (GUS) expression. The resulting construct, *ProOsEAF6:GUS*, was transformed into Asominori plants. For GUS staining, tissue samples (i.e., leaf, young spikelets, and stem) were vacuum-infiltrated with staining solution (50 mM Na_3_PO_4_, pH 7.0, 1.0 mg/ml 5-bromo-4-chloro-3-indolyl-β-glucuronide, and 0.5% Triton X-100) and incubated at 37°C for 16 h. The stained samples were fixed for 10 min in formaldehyde, acetic acid, and 22% ethanol (5:5:90, v/v) and then destained in 100% ethanol until the chlorophyll was removed.

### Subcellular Localization Analysis

Transient expression assays using polyethylene glycol-mediated transformation were performed as previously described (Shen et al., 2014). The *35S:OsEAF6-mCherry* construct was prepared by amplifying mCherry from the pmCherry-C1 vector (Takara) and inserting it into the plant binary vector pRI201-ON (Takara). The coding sequence (CDS) of *OsEAF6* (*LOC_Os12g20310*) was cloned into the pCR-Blunt II TOPO vector (Life Technologies) and transferred into the mCherry-pRI201-ON vector. Rice Oc cells were provided by RIKEN BRC through the National Bio-Resource Project of MEXT, Japan. Protoplasts from Oc cells were adjusted to a concentration of 1.0–2.0 × 10^6^ cells/ml; a 0.1-ml aliquot was transfected with 10–20 μg plasmids. After 16 h of incubation at 28°C, transformed cells were observed under an optical/fluorescence microscope (Biozero BZ-8000, Keyence, Osaka, Japan).

### Genome Sequence and Haplotype Analysis

The genome sequences of cultivated and wild rice accessions were downloaded from the Gramene database^2^. Genome sequences of the *japonica* variety Nipponbare (IRGSP version 1.0) and *indica* varieties IR8 (IOMAP version 1) and 93-11 (ASM465v1) were used to compare chromosomal structural differences between *indica* and *japonica* around the *HWE1* and *HWE2* regions. The genomic sequences were identified using GenomeMatcher version 2.03 software (Ohtsubo et al., 2008). The genome sequences of the following species were also used: *Oryza nivara*, accession W0106 (version AWHD00000000); *Oryza rufipogon*, W1943 (PRJEB4137); *Oryza barthii*, IRGC105608 (ABRL00000000); *Oryza glaberrima*, IRGC96717 (AGI1.1); *Oryza glumaepatula*, GEN1233_2 (ALNU02000000); *Oryza meridionalis*, W2112 (Oryza_meridionalis_v1.3); *Oryza punctata*, IRGC105690 (AVCL01000000); *Oryza brachyantha*, IRGC101232 (AGAT00000000), and *Leersia perrieri*, IRGC105164 (version 1.4). Gene annotation of Leersia was based on the Gramene database, and the others were based on RiceGAAS (Sakata et al., 2002). Haplotype analysis of the *HWE1* and *HWE2* loci was performed based on published SNP data for cultivated and wild rice compared with the Nipponbare genome. SNP data for cultivars and wild rice were obtained from the International Rice Information System^3^ and OryzaGenome^4^, respectively.

### Phylogenetic Analysis of EAF6 Protein Homologs

A search for EAF6 homologs in different organisms was performed using the BLASTP program (NCBI) website. Protein sequences of 25 plant species and 8 microbe and animal species were obtained from phytozome^5^ and the NCBI database (Supplementary Table 2). Protein sequences were aligned using ClustalW to construct a phylogenetic tree using the neighbor-joining method (Saitou and Nei, 1987) and MEGA6 software (Tamura et al., 2013). Bootstrap values were calculated with 1,000 replications.

## Results

### Characteristics of Hybrid Breakdown

**FIGURE 1:**
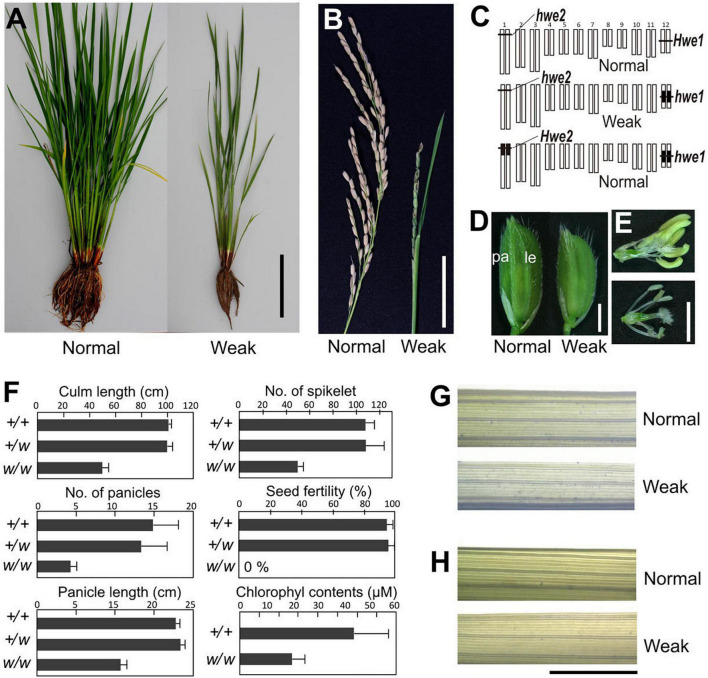
Morphology of weak plants. **(A)** Plant morphology at vegetative stage 3 weeks before heading. **(B)** Morphology of panicle at the mature seed stage. Weak plants exhibited a partially sheathed panicle. **(C)** Graphical genotype of weak plants. White and black bars represent Asominori and IR24 chromosomes, respectively. Only the double recessive homozygote showed the weakness phenotype. **(D)** Spikelets of normal (*left*) and weak plants (*right*). Le, lemma; pa, palea. **(E)** Flower organs of normal (*upper*) and weak plants (*lower*). **(F)** Characterization of weak plants. Column length, number of panicles, number of spikelets per panicle, seed fertility, and leaf chlorophyll contents at 3 weeks before flowering. +/+: *Hwe1/Hwe1 hwe2/hwe2*, +/w: *Hwe1/hwe1, hwe2/hwe2*, and w/w: *hwe1/hwe1 hwe2/hwe2*. *N* = 10 for each genotype excluding the chlorophyll contents (*N* = 5). **(G,H)** Trypan blue staining to assess viability leaf blade cell. Third youngest leaf blades from normal and weak plants at the developmental stage before heading [**(G)**, 90 days after sowing] and after heading [**(H)**, 120 days after sowing) were stained by trypan blue. Neither showed remarkable cell lethality. Scale bar = 10.0 cm in **(A)**, 5.0 cm in **(B)**, 2.0 mm in **(D,E)**, and 5.0 mm in **(G,H)**.

A single substitution with *indica* (cv. IR24) chromosome 12 in a *japonica* (Asominori) genetic background causes hybrid breakdown characterized by poor growth and complete sterility (Kubo and Yoshimura, 2002). Simple epistasis with double recessive genes, named *hwe1* and *hwe2*, was sufficient to explain this hybrid breakdown (Figures 1A–C). Similarly, genetic analysis showed that epistasis between *hwe1* and *hwe2* caused a hybrid breakdown in other cross combinations (Nipponbare/93-11 and Nipponbare/IR8) (Supplementary Figures 1, 2). The weak phenotype was characterized by shorter and smaller culm lengths, partially sheathed panicles, and both male and female sterility (Figures 1B–F and Supplementary Figures 3A,B). The weak segregant appeared as pale green compared with normal plants at the adult stage, likely due to the lower chlorophyll content in the leaves (approximately half of that in the normal segregant) (Figure 1F). Leaves from weak plants during the vegetative growth stage were not stained by trypan blue (Figures 1G,H), suggesting that this weak phenotype did not result from an autoimmune response by nucleotide-binding site-leucine-rich repeat or other related molecules, as previously reported (Bomblies et al., 2007; Alcazar et al., 2010). A much more severe phenotype was observed in the reproductive organs. Weak plants produced smaller panicles and yielded fewer spikelets compared with normal plants (Figures 1B,F). An abnormal phenotype was also found in the reproductive organs, such as depressed palea or palea-less flowers, degenerated anthers, and abnormal stigma formation (Supplementary Figures 4A–L). Microscopy revealed that pollen sterility was attributed to meiotic defects, including abnormal or incomplete cell division (Supplementary Figures 3C–M). Although the female gametes were also completely sterile, various ovules swollen by imbibition were observed without fertilization in weak plants (Supplementary Figure 4M).

### Identification of *HWE1* and *HWE2*

**FIGURE 2:**
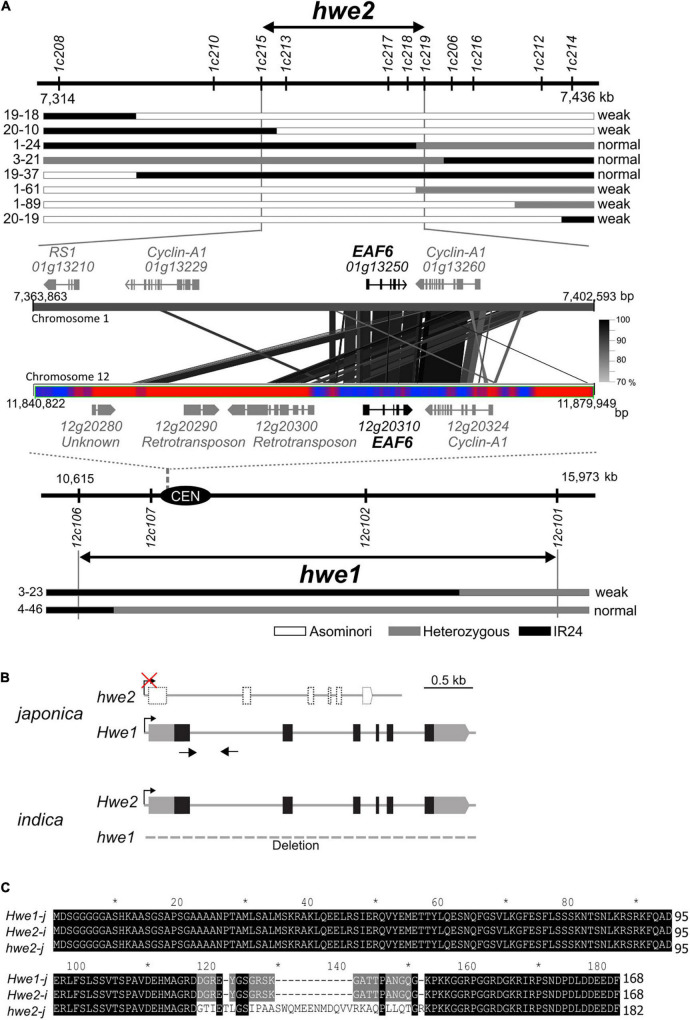
Identification of causal genes for *hwe1* and *hwe2*. **(A)** Map locations of *hwe2* (*upper*) and *hwe1* (*lower*), and MSU7.0 gene annotation in candidate regions. The prefix “LOC_” in locus ID is omitted for convenience. The most informative recombinant genotypes are shown along with the maps. Similarity of DNA sequences between chromosomes 1 and 12 is shown as connected with black and gray lines. The bar with red and blue heat map represents the abundance of repetitive DNA sequences around the centromere (CEN) of chromosome 12. Red represents the repeat region. **(B)** Gene structure of *HWE1* and *HWE2* of *japonica* (Asominori) and *indica* (93-11). Gray and black boxes denote untranslated region (UTR) and coding sequence (CDS) of rice *OsEAF6*, respectively. The CDS sequences of *OsEAF6* were identical between *japonica* and *indica*. **(C)** Multiple alignments of predicted OsEAF6 protein sequences of *japonica* (*Hwe1-j* and *hwe2-j*) and *indica* (*Hwe2-i*).

*HWE1* and *HWE2* have been roughly mapped to rice chromosomes 12 and 1, respectively (Kubo and Yoshimura, 2002). Using the small-scale mapping population (*N* = 383), we identified the *HWE1* locus within a 5.4-Mb region close to the centromere of chromosome 12 (Figure 2A). As meiotic recombination is repressed around the centromere and pericentromeric regions, we then focused on the partner gene *HWE2*. To isolate *HWE2*, we screened recombinant individuals from a large segregating population (*N* = 2,387). The result showed that the *HWE2* locus was delimited within a 38.7-kb region between the PCR markers *1c215* and *1c219*, which encoded four predicted genes (i.e., *LOC_Os01g13210*, *LOC_Os01g13229*, *LOC_Os01g13250*, and *LOC_Os01g13260*) (Figure 2A). Of these four genes, two genes (i.e., *LOC_Os01g13250* and *LOC_Os01g13260*) shared homology with *LOC_Os12g20310* and *LOC_Os12g20324* in the *HWE1* region, indicating a small segmental duplication between chromosomes 1 and 12 of the Nipponbare genome. In comparative sequence analysis, the DNA sequence of the *indica Hwe2* allele showed much greater similarity to that of the *japonica Hwe1* allele than to that of the *japonica hwe2* allele (Supplementary Figure 5). However, there were no segmental blocks corresponding to *LOC_Os12g20310* and *LOC_Os12g20324* on *indica* chromosome 12 (93-11 and IR8 genomes). In the context of double recessive epistasis, these duplicated genes were considered good candidates for *HWE1*/*2*. The *LOC_Os01g13250* and *LOC_Os12g20310* loci encode *EAF6*, and the adjacent *LOC_Os01g13260* and *LOC_Os12g20324* encode the cyclin-A1 protein. We performed complementation analysis to determine whether one or both of these genes contribute to hybrid breakdown. Due to the complete sterility of weak segregants, we transformed *Hwe1/hwe1* heterozygotes with Asominori genomic DNA containing *LOC_Os12g20310* or *LOC_Os12g20324* and then evaluated the complementation in their selfed progeny (T1 and T2 generations). The resultant transformants with *LOC_Os12g20310* recovered the weak growth phenotype with complete sterility, whereas the other transformants with *LOC_Os12g20324* did not (Supplementary Figure 6 and Supplementary Tables 3, 4). This result indicates that *OsEAF6*, encoded by *HWE1*/*2*, was responsible for the hybrid breakdown. A full-length cDNA of *OsEAF6* has been previously cloned (GenBank, CT830710). The gene structure of the Nipponbare allele of *LOC_Os01g13250* was predicted to encode a variant form of *OsEAF6* with extra amino acids (14 extra amino acids) in the C-terminus (Figures 2B,C and Supplementary Figure 7). We predicted that this variant form (*hwe2-j*) was functionally defective.

### Hybrid Breakdown Attributed to Loss of *OsEAF6* Expression

**FIGURE 3:**
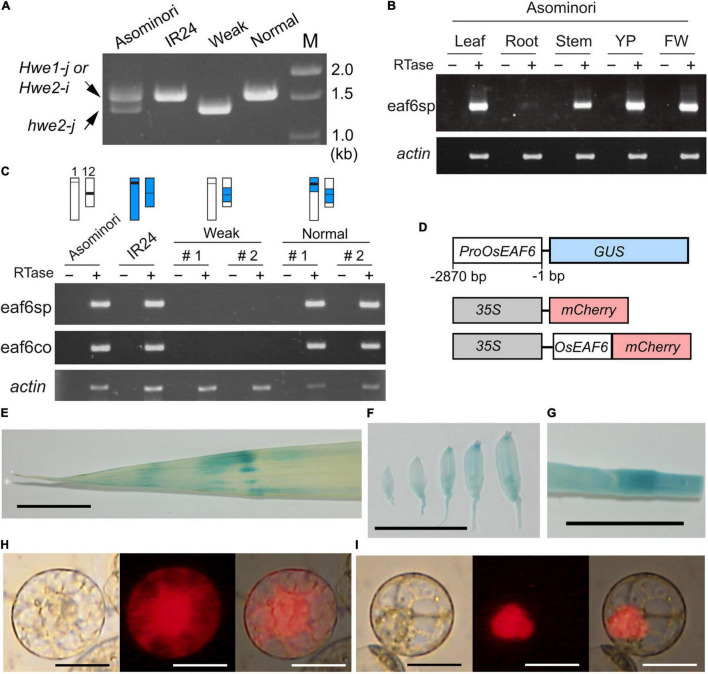
Expression pattern and cellular localization of the OsEAF6. **(A)** Amplified genomic DNA product obtained with the primer eaf6co. The expected sizes of DNA bands were 1,557 bp from *Hwe1-j and Hwe2-i* and 1,352 bp from *hwe2-j.* The primer eaf6co was designed for the identical sequence region between *Hwe1-j* (functional gene) and *hwe2-j* (pseudogene). It is noted that the eaf6co primer could amplify the product from genomic DNA but not from mRNA, as shown in **(C)**. **(B)** mRNA expression of *OsEAF6* in rice tissues detected using RT-PCR analysis. eaf6sp is a primer set specific to functional *OsEAF6* [*japonica Hwe1* (*Hwe1-j*) allele and *indica Hwe2* (*Hwe2-i*) allele] but not the pseudogene on the *hwe2-j* allele. YP, young panicle; FW, flowering panicles **(C)** mRNA expression of *OsEAF6* in the NILs and parents with the two different primer sets by RT-PCR analysis. Genotype of each sample for chromosomes 1 and 12 is shown on the upper part. White and blue bars represent Asominori and IR24 chromosomes, respectively. Two individuals for the weak and normal plants (#1 and 2) are shown. **(D)** Diagram of constructs designed for expression analysis of *OsEAF6.* **(E–G)** GUS expression analysis of *OsEAF6* by using *ProOsEAF6:GUS* expresser in leaf **(E)**, developing flowers **(F)**, and stem **(G)** tissues. **(H,I)** Subcellular localization of OsEAF6 protein fused with mCherry **(I)** and mCherry alone **(H)** in rice protoplast cells. Transient expression of each fluorescent construct was observed. Left, DIC image; middle, mCherry channel image; right, merged image. Scale bar = 1.0 cm in **(E–G)**, and 20 μm in **(H–I)**.

To verify this hypothesis, we examined the expression of *LOC_Os12g20310* and *LOC_Os01g13250* in Asominori and IR24 using RT-PCR with two primer sets, namely, eaf6sp and eaf6co. The first primer set, eaf6sp, was specific to Nipponbare *LOC_Os12g20310* but not to *LOC_Os01g13250*, which encodes the valiant form. The second primer, eaf6co, matched the conserved identical sequence between *LOC_Os12g20310* and *LOC_Os01g13250* and was used to examine the presence of the variant mRNA from *LOC_Os01g13250* (Figure 3A). The mRNA from *LOC_Os12g20310* was ubiquitously expressed in all organs examined, except for the root, which showed faint expression (Figure 3B). *OsEAF6* was not expressed in the weak plants, whereas co-introgression with the IR24 segment around the *HWE1* and *HWE2* loci recovered the expression (Figure 3C), indicating that active *OsEAF6* mRNA was generated from the *japonica Hwe1-j* and *indica Hwe2-i* alleles. The absence of the mRNA signal from *LOC_Os01g13250* with the eaf6co primer indicated that *japonica hwe2-j* is an *OsEAF6* pseudogene (Figure 3C). Thus, these results support that defects were present in functional *OsEAF6* on the recessive *hwe2-j* and *hwe1-i* alleles and indicate that the lack of *OsEAF6* on the *hwe1* and *hwe2* alleles induces hybrid breakdown.

### Tissue and Subcellular Localization of OsEAF6

In higher plants, EAF6 proteins are 144–170 amino acids long and highly conserved throughout herbaceous and woody plants (Supplementary Figure 8 and Supplementary Table 2). Their sequences are partially conserved with those of yeast and animal species. Yeast (*Saccharomyces cerevisiae*) EAF6 is a small protein (113 amino acids) and subunit of the NuA4 HAT complex that is involved in transcriptional regulation through nuclear H4 acetylation (Mitchell et al., 2008). To gain insight into the function of OsEAF6, we examined the expression of *OsEAF6* in transgenic rice plants with *ProOsEAF6:GUS* (Figure 3C). *GUS* expression was observed in vegetative organs, including the leaves and stems, and in developing spikelets (Figures 3D–G). This result is consistent with those of RT-PCR analysis. Subcellular localization analysis using rice Oc cells showed that OsEAF6 protein was present predominantly in the nucleus and, to a lesser extent, in the cytoplasm, whereas the control mCherry plasmid was detectable throughout the cell (Figures 3H,I). This result indicates that OsEAF6 functions in the nucleus.

### Evolution of *EAF6* in *Oryza* Species

**FIGURE 4:**
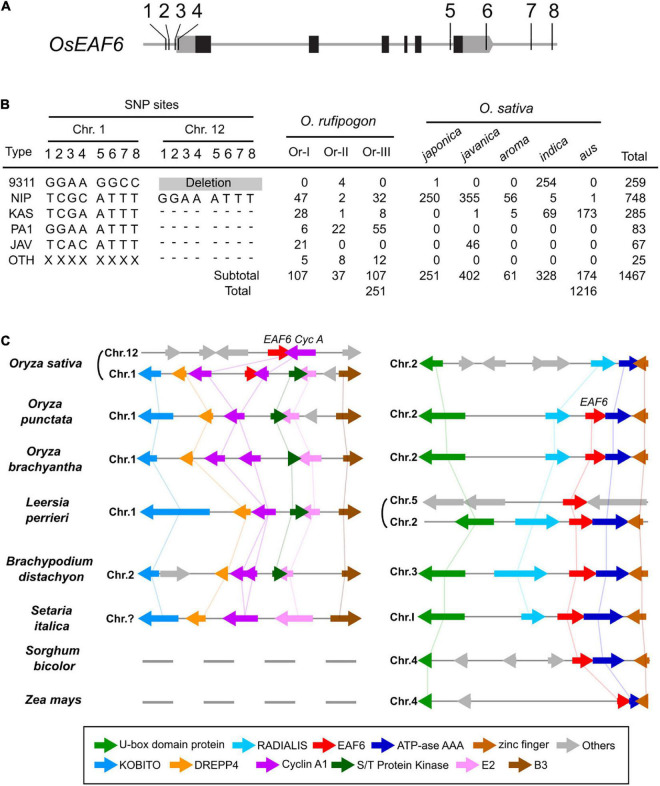
Allele distribution of *OsEAF6* and syntenic gene analysis of *OsEAF6* region. **(A)** Distribution of *hwe1* and *hwe2* alleles in *Oryza sativa–Oryza rufipogon* complex. Positions of eight SNPs (1–8) on the 5′ and 3′ regions of *OsEAF6*. Gray and black boxes denote UTR and CDS of rice *OsEAF6*, respectively. SNP site 1, -309 bp on chr.1 and -304 bp on chr.12; site 2, -299 bp on chr.1 and -294 bp on chr.12; site 3, -205 bp on chr.1 and -206 bp on chr.12; site 4, -157 bp on chr.1 and -158 bp on chr.12; site 5, +2,133 bp on chr.1 and +2,527 bp on chr.12; site 6, +2,494 bp on chr.1 and +2,890 bp on chr.12; site 7, +2,971 bp on chr.1, and site 8, +3,153 bp on chr.12 and +3,555 bp on chr.12. The positions of SNPs (bp) were based on the Nipponbare sequence [+1 refers to **(A)** in the ATG start codon]. **(B)** Distribution of six haplotypes of duplicated *OsEAF6* in *O. rufipogon* and *O. sativa*. We evaluated 251 accessions for *O. rufipogon* and 1,216 accessions for *O. sativa*. The six haplotypes based on eight SNP sites consist of 93-11-type (9311), Nipponbare-type (NIP), Kasalath-type (KAS), PA1-type (PA1), *javanica* type (JAV), and other type (OTH). Hyphens represent identical SNPs with the Nipponbare SNP. **(C)** Conserved syntenic block harboring *EAF6* and adjacent *Cyclin-A1* loci. Gene annotation of the region surrounding the *EAF6* (*right*) and *Cyclin-A1* (*left*) loci in the cereal species. Homologous genes are shown in the same color connected by straight lines.

In the rice genome sequencing project, 450 *O. rufipogon* accessions and 3,000 cultivars were sequenced using next-generation sequencing techniques (Huang et al., 2012; Wang W. et al., 2018). Based on published genome sequence data, we investigated the distribution of *hwe1* and *hwe2* alleles in cultivars and their wild relative *O. rufipogon*. The allelic diversity of duplicated *hwe* loci was examined based on eight SNPs at the 5′ and 3′ terminal regions of *OsEAF6*, which can discriminate between alleles in *HWE1* and *HWE2*. The Nipponbare *Hwe1* allele (called Nip-type) is GGAA-ATTT and is common among the *O. sativa-O. rufipogon* complex. The 93-11 *hwe1* null allele (9311-type) appeared to be distributed in the *indica* ecotype (255 accessions) but was minor in *O. rufipogon* (Figures 4A,B and Supplementary Table 5). We characterized the Nipponbare *hwe2* null allele as TCGC-ATTT (Nip-type) and 9311 *Hwe2* allele as GGAA-GGCC. Most *japonica* subspecies varieties (99.6%, 250/251) and 30–44% of *O. rufipogon* Or-I and Or-III ecotypes carried the Nip-type *hwe2* allele. Since Or-III has been reported as a progenitor of *japonica*, *hwe2* of *japonica* rice may have originated from *Or-III.* Some *O. rufipogon* accessions and *O. sativa* ssp. *indica “aus”* ecotype had two copies of functional *OsEAF6* on chromosomes 1 and 12 (Supplementary Figure 7).

To determine the origin and timing of *EAF6* duplication, *EAF6* homologs were investigated in wild *Oryza* species. We first analyzed the AA genome species most closely related to *O. sativa*. A BLAST search of the LOC_Os12g20310 CDS showed hits on chromosome 1 of other AA genome species (Supplementary Figure 9). Then, we investigated a local synteny pattern around the *EAF6* locus across distantly related *Oryza* species and five grass species. *Oryza* BB and FF genome species and other grass species (*Leersia*, *Brachypodium*, and *Setaria*) showed large blocks of homologous synteny around the *HWE2* region of Nipponbare chromosome 1 but lacked the whole sequence of *EAF6* on this syntenic block. Instead, these species contained a single copy of *EAF6* on other syntenic chromosomes, which were syntenic to *O. sativa* chromosome 2 (Figure 4C). The closest genus *Leersia*, which carried two copies of *EAF6*, showed that the local gene order around the *EAF6* locus on *Leersia* chromosome 5 did not differ among other species, indicating independent duplication events between rice and *Leersia*. The gene order and orientation around the *Cyclin-A1* locus (*LOC_Os01g13260*) were conserved on chromosome 1 of *O. punctata*, *O. brachyantha*, and *Leersia* as a single copy segment. Based on the alignment of other AA genome species, this result suggests that *EAF6* was initially transposed to chromosome 1 from chromosome 2 in the AA genome progenitor, followed by segmental duplication to chromosome 12 (Supplementary Figure 10).

## Discussion

We demonstrated that hybrid breakdown is caused by *HWE1*/*2* encoding a rice homolog of the NuA4 HAT complex subunit protein EAF6. The NuA4 HAT complex is an essential transcriptional coactivator involved in gene regulation, cellular processes, and DNA double-strand break repair in eukaryotes. Yeast EAF6 interacts with another catalytic subunit protein, Esa1, *via* Yng2. The functional role of EAF6 protein in plants remains unclear. We found that the loss of the OsEAF6 protein exerted deleterious pleiotropic effects on both vegetative growth and reproductive development in rice. Particularly, it has a broad impact on reproductive development, ranging from inflorescence development to gametogenesis. During the preparation of this study, Zhou et al. (2022) found that the *Arabidopsis eaf6* mutant shows growth inhibition and leaf yellowing and that the NuA4 complex is involved in transcriptional activation, specifically in light-responsive genes. Another research group reported that the loss of *Arabidopsis* Esa1-associated factor 1 (EAF1) inhibited growth and chloroplast development (Bieluszewski et al., 2022). These findings are consistent with our phenotypic observations, such as growth inhibition with reduced chlorophyll content during the vegetative phase in the double homozygote *hwe1/2*. It is suggested that such deleterious pleiotropic phenotypes occurred due to disorders of the universal chromatin state and transcriptional regulation caused by the loss of OsEAF6 protein.

### Hypothetical Evolutionary History

Extensive genome sequencing and comparative studies revealed conserved microsynteny (gene order patterns) across different cereal species (Jaiswal et al., 2006). Similar to previously reported microsynteny, a conserved gene order around *EAF6* was observed among the monocot crop species, including the wild rice relatives *Leersia* and sorghum (Figure 4C). However, the chromosome position of this synteny block containing *EAF6* was not chromosome 12 or chromosome 1, but rather chromosome 2 in other wild rice species (*O. punctata* and *O. brachyantha*). We hypothesized the evolutionary history of *EAF6* in *Oryza* genomes as follows: (1) *EAF6* has resided on chromosome 2 of primitive *Oryza* species; (2) *EAF6* was transposed to chromosome 1 in an early AA genome progenitor; (3) a segmental duplication occurred and was positioned on chromosome 12 in a subpopulation of *O. rufipogon*; and (4) one copy of the gene was lost in a progenitor population of *O. sativa* ssp. *japonica* (Supplementary Figure 10). This hypothetical scenario was based on the chromosome synteny and distribution of SNPs discriminating the two *OsEAF6* copies in the cultivars and their close relatives (Figures 4A,C). Transposition to chromosome 1 was considered for the following reasons. First, *Cyclin-A1* is not found on the corresponding region of chromosome 12 in primitive *Oryza* species. Second, the *EAF6* CDS was localized on chromosome 1 according to recent next-generation sequencing analyses of other AA genome species (Supplementary Figure 7). Thus, in evolutionary history, *OsEAF6/HWE1* was a copy of *OsEAF6/HWE2* following the transposition from chromosome 2. Other grass species retain microsynteny around the *EAF6* positions. The mechanisms of transposition and duplication of *OsEAF6* remain unclear. Despite the positional differences in *EAF6* in grass species, the protein sequence of EAF6 protein is largely conserved among plant species (Supplementary Figure 8), suggesting that it has an essential function in plant development. Therefore, duplicated *EAF6* in other plant genomes may function as a reproductive isolation system.

### Functional Role as the Reproductive Isolation System

In some animal studies, DNA-binding proteins, such as OdsH, PRDM9, and Zhr, were identified as causal molecules for hybrid sterility (Maheshwari and Barbash, 2011). These factors are likely associated with the dysfunction of chromatin remodeling in heterozygous hybrid progenies. Thus, abnormal chromatin formation during meiotic cell division in hybrids is a common factor responsible for reproductive isolation. From the perspective of the reproductive isolation mechanism, hybrid breakdown by *hwe1/2* occurred due to the loss of gene function and differed from the disharmonious interactions in the animal cases mentioned above, although the mechanism of action targeting nucleosomes is similar. Since no remarkable changes were observed in the heterozygous state, we did not characterize the detailed phenotype of heterozygous plants. However, heterozygous plants for each single locus of *HWE1* and *HWE2* (i.e., *Hwe1/hwe1 hwe2/hwe2* and *hwe1/hwe1 Hwe2/hwe2*) induced reduced transmission of the recessive alleles (*hwe1* and *hwe2*) in the selfed progeny (Kubo and Yoshimura, 2002). Thus, *hwe1/2* strongly impacts the elimination of the specific genotype around these genes in the hybrid population. Additionally, OsEAF6 may be involved in the haplotype gamete phase in rice. According to previous microarray data and laser capture microdissection of male and female gametes (Hobo et al., 2008; Kubo et al., 2013), *OsEAF6* was substantially and constantly expressed in haploid organs, such as microspores and megaspores (Supplementary Figure 11). Furthermore, the involvement of NuA4 in gametogenesis has been previously reported in *Arabidopsis* (Latrasse et al., 2008). Therefore, we believe that OsEAF6 may regulate histone acetylation and transcription levels throughout the rice life-cycle including the diploid and haploid phases. Further studies are required to determine the functions of OsEAF6 as a subunit of the HAT complex in various developmental stages and tissues. This study demonstrated the involvement of EAF6 in plant development and reproductive isolation. These findings will provide a helpful clue to transcriptional regulation by histone acetylation in plant development and also aid to develop an efficient breeding program to overcome reproductive isolation.

## Data Availability Statement

The datasets presented in this study can be found in online repositories. The names of the repository/repositories and accession number(s) can be found in the article/Supplementary Material.

## Author Contributions

TK, AY, and NK conceived and designed the experiments. TK performed the experiments, analyzed the data, and wrote the study, with input from AY and NK. All authors read and approved the final manuscript.

## Conflict of Interest

The authors declare that the research was conducted in the absence of any commercial or financial relationships that could be construed as a potential conflict of interest.

## Publisher’s Note

All claims expressed in this article are solely those of the authors and do not necessarily represent those of their affiliated organizations, or those of the publisher, the editors and the reviewers. Any product that may be evaluated in this article, or claim that may be made by its manufacturer, is not guaranteed or endorsed by the publisher.

## Footnotes

1
http://rice.plantbiology.msu.edu/

2
http://ensembl.gramene.org/Oryza_sativa/Info/Index

3
http://oryzasnp.org/iric-portal/index.zul

4
http://viewer.shigen.info/oryzagenome/mapview/Top.do

5
https://phytozome-next.jgi.doe.gov
