# Supplementary material for: Loss of OsEAF6, a Subunit of the Histone Acetyltransferase Complex, Causes Hybrid Breakdown in Intersubspecific Rice Crosses
Source: Front Plant Sci. 2022 Mar 8;13:866404. doi: 10.3389/fpls.2022.866404 (PMC8957887; doi:10.3389/fpls.2022.866404)
Supplement: Supplementary file 1 [file Data_Sheet_1.PDF]

Supplementary Materials

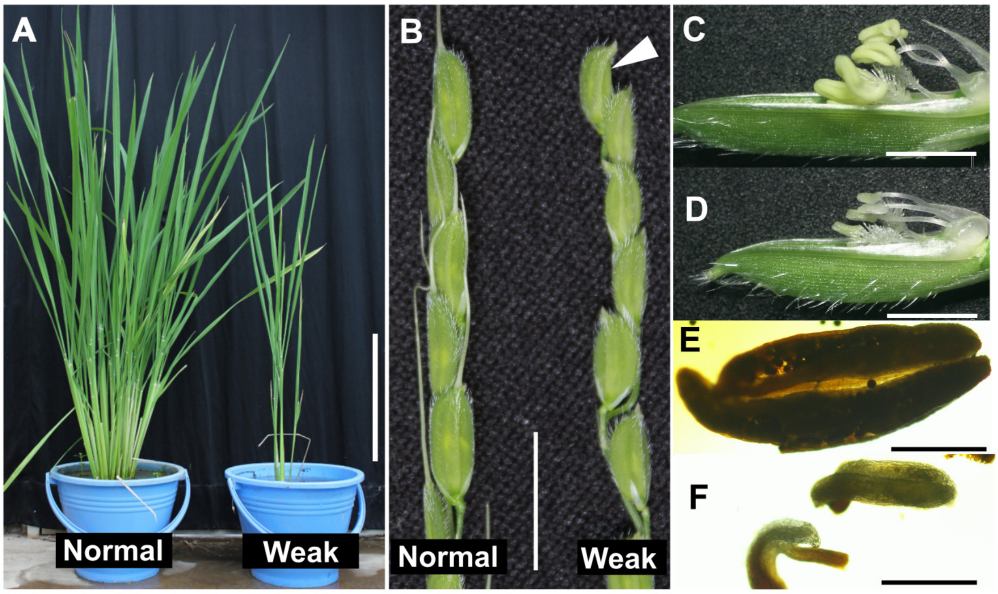

G Genotype		*HWE2*			
		II	IJ	JJ	Total
HWE1	II	Normal 0	Normal 5	Weak 5	10
	IJ	Normal 1	Normal 7	Normal 15	23
	JJ	Normal 1	Normal 10	Normal 4	15
	Total	2	22	24	48

Supplementary Figure 1. Genetics of hybrid breakdown and plant morphology of a weak segregant in a Nipponbare/93-11 population. (**A**) Plant morphology of normal (left) and weak plants (right) found in a BC_2F_2 population of Nipponbare/93-11//Nipponbare. (**B**) Panicle branches of normal and weak plants. Arrowhead represents the depressed palea which is characteristic of weak plants. (**C, D**) Flower organs of normal (**C**) and weak plants (**D**). (**E, F**) Anthers of normal (**E**) and weak plants (**F**), stained using I_2-KI solution. (**G**) Inheritance of weak plants in a selfed progeny (N = 45) of a BC_2F_1 plant heterozygous for both the *HWE1* and *HWE2* loci. DNA markers 12c106 and 1c203 were used to genotype the *HWE1* and *HWE2* loci, respectively. Numbers indicate the number of plants for each genotype class in the population. Genotype is: II: 93-11 homozygote, IJ: heterozygote, JJ: Nipponbare homozygote. Scale bar = 30 cm in (**A**), 10 mm in (**B**), 2 mm in (**C, D**), and 0.5 mm in (**E, F**).

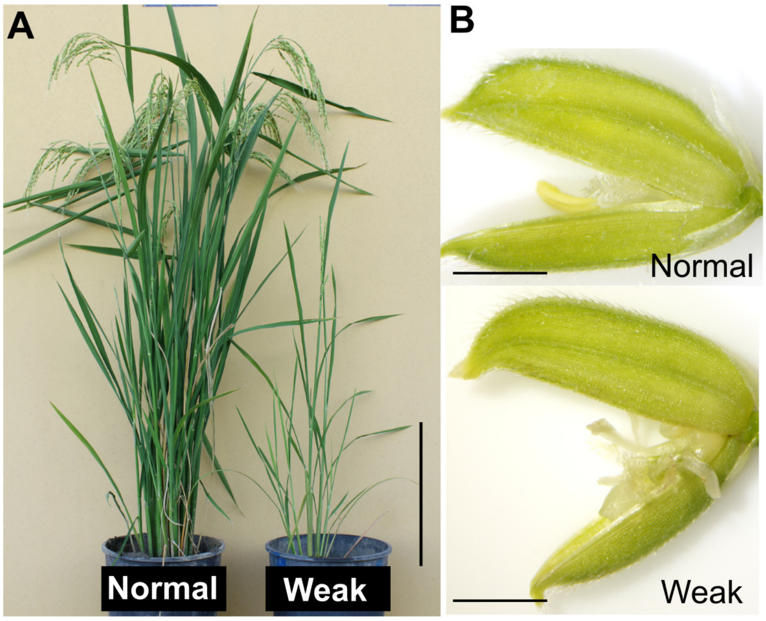

C

Genotype		*HWE2*			
		II	IJ	JJ	Total
HWE1	II	Normal 17	Normal 17	Weak 3	37
	IJ	Normal 28	Normal 34	Normal 24	86
	JJ	Normal 17	Normal 16	Normal 10	43
	Total	62	67	37	166

Supplementary Figure 2. Genetics of hybrid breakdown and plant morphology of a weak segregant in a Nipponbare/IR8 population. (**A**) Plant morphology of normal (*left*) and weak plants (*right*) found in an F_2 population of Nipponbare/IR8. Scale bar = 30 cm. (**B**) Spikelets of normal and weak plants. Scale bar = 2 mm. (**C**) Inheritance of weak plants in a Nipponbare/IR8 F_2 population (N = 166). DNA markers 12c106 and 1c203 were used to genotype the *HWE1* and *HWE2* loci, respectively. Numbers indicate the number of plants for each genotype class in the population. Genotypes are: II: IR8 homozygote, IJ: heterozygote, JJ: Nipponbare homozygote.

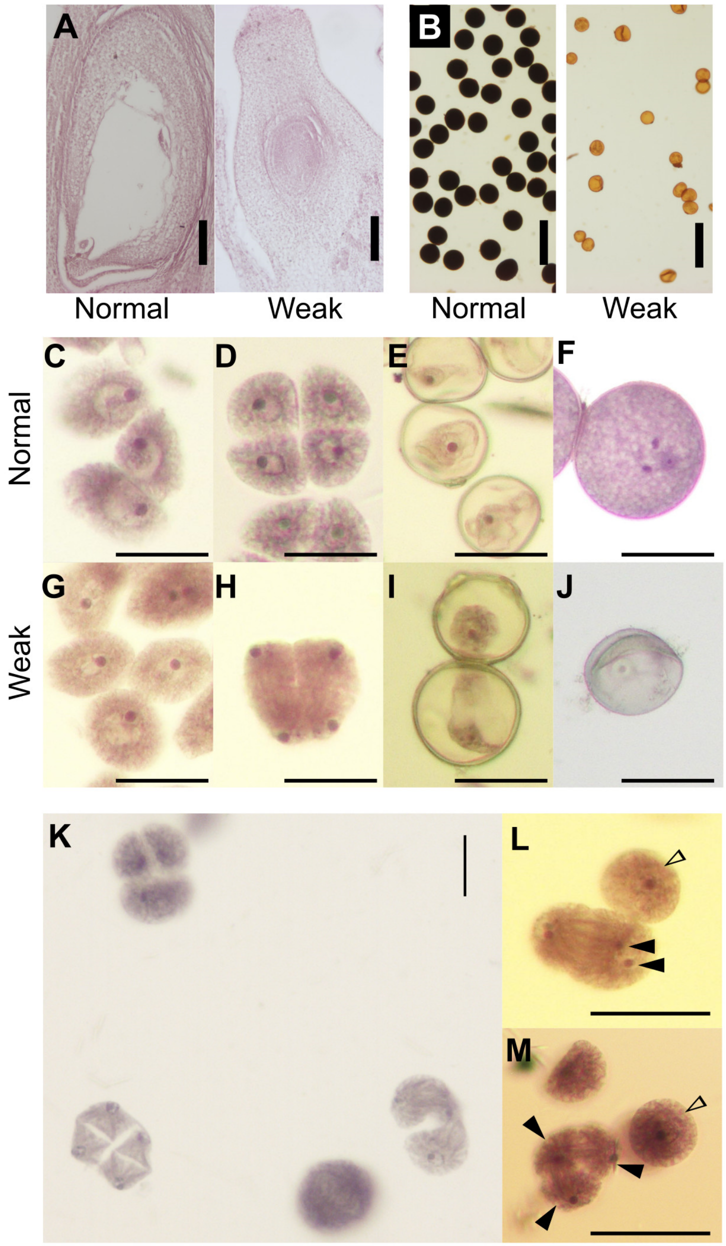

Supplementary Figure 3. Defective gametogenesis in weak plants. (**A**) Embryo sac at flowering stage, stained by toluidine blue O. (**B**) Pollen grains at flowering stage, stained by I_2-KI. (**C–F**) Normal microsporogenesis in a normal segregant. (**G–J**) Abnormal microsporogenesis in a weak segregant. Sporophyte cells at meiotic stage (**C, G**), tetrad (**D, H**), mononucleate microspore (**E, I**), and pollen grains at matured stage (F, J). One microspore developed to the mononucleate stage, whereas the others showed defective meiotic divisions and abnormal tetrad cells (**K–M**). Scale bars = 0.1 mm in (**A–B**), and 20 μm in (**C–M**).

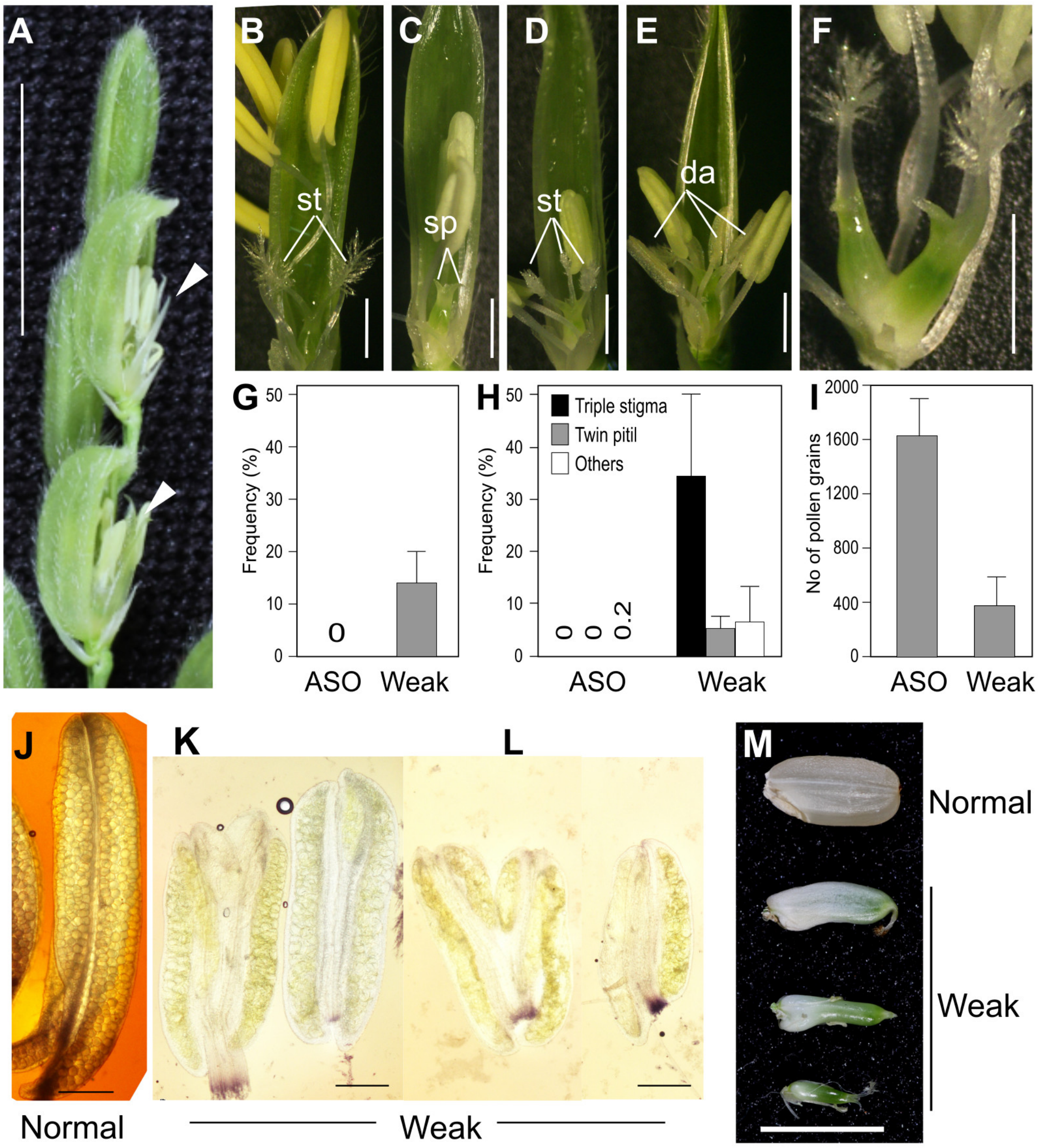

Supplementary Figure 4. Flower organs of weak plants (**A**) Palea-less flower of the weak plant. Lemmas were normally developed, but paleas were degenerated (arrowhead), resulting in exposed inner organs. mrp: marginal region of palea. (**B–F**) Flower organs from normal (**B**) and weak plants (**C–F**). st: stigma, sp: stigmaless pistil, degenerated dpa: degenerated palea, da: degenerated anther. (**B**) Normal flower organs bearing six anthers and one pistil with two stigmas in Asominori, (**C**) stigmaless pistil, (**D**) pistil with three stigmas, and (**E**) degenerated anthers differing in degree. In this panel, four of six anthers were severely degenerated. (**F**) Twin pistils. (**G**) Frequency of depressed paleas per panicle in weak plants compared to in Asominori ($N = 5$). (**H**) Frequency of abnormal pistils in weak plants compared to in Asominori ($N = 5$). (**I**) Number of pollen grains in a single anther from Asominori and weak plant. To evaluate weak plants, the number of empty pollens from the moderate degenerated anthers of weak plants was counted ($N = 3$). (**J**) Normal anther from a single spikelet of the normal plant, stained with hematoxylin. (**K–L**) Degenerated anthers from a single spikelet of a weak plant, stained with hematoxylin. Two anthers (**K**) were likely arrested at earlier developmental stage compared to the other two (**L**) which did not have visible microspores. (**M**) Mature seed from a normal plant and hypertrophic pistils without fertilization from weak plants. Scale bar = 10.0 mm in (**A**), 1.0 mm in (**B–F**), 0.25 mm in (**J–L**), and 5.0 mm in (**M**).

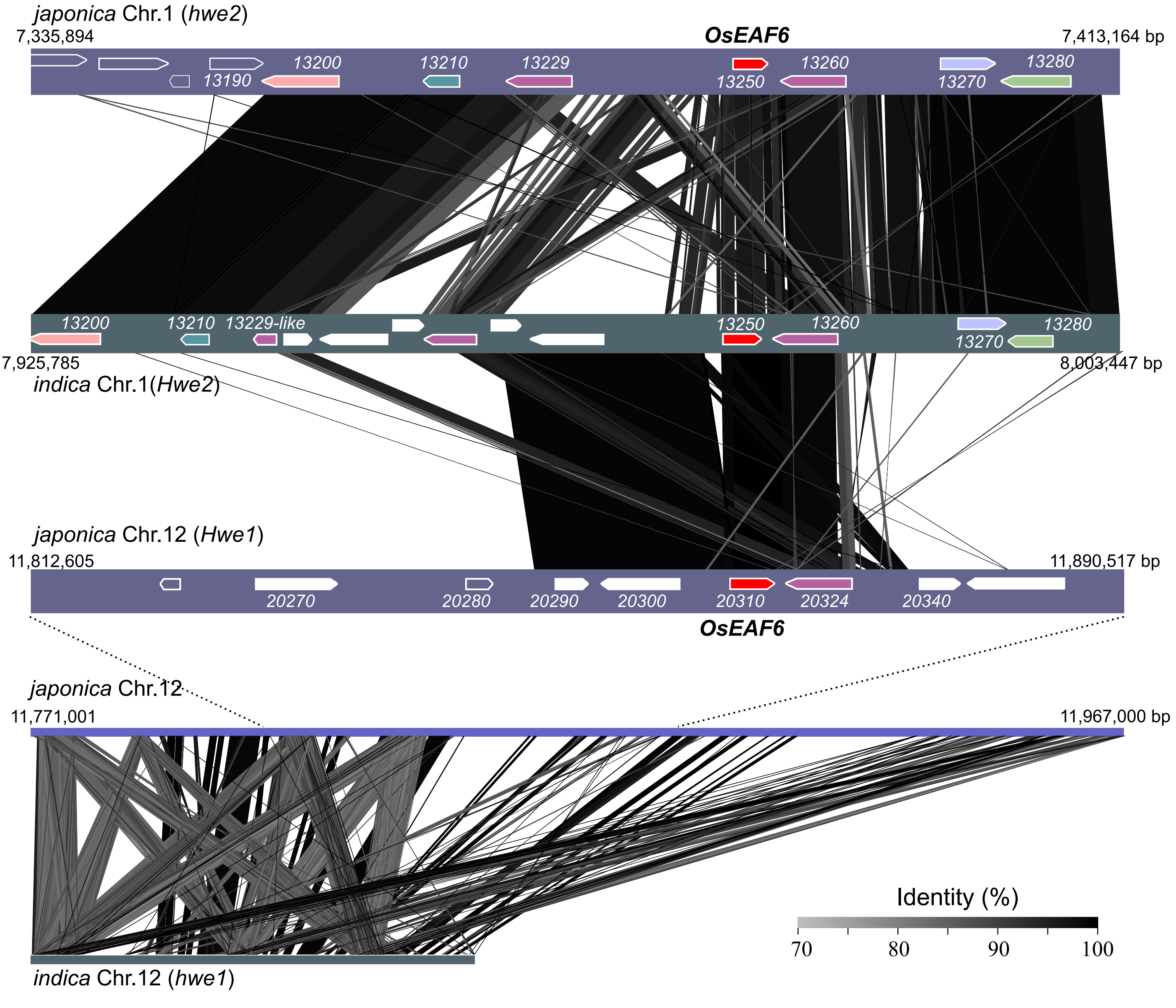

Supplementary Figure 5. Genomic comparison of duplicated region around OsEAF6 locus on chromosome 1 and 12. Percent identity plot between Nipponbare (*japonica*) and 93-11 (*indica*) genome sequences. Color density represents % nucleotide identity. The same genes are shown in the same color to indicate gene order conservation. White solid box denotes transposable element. For convenience, only the last five letters of the locus ID are shown (LOC_Os01gXXXXX or LOC_Os12gXXXXX).

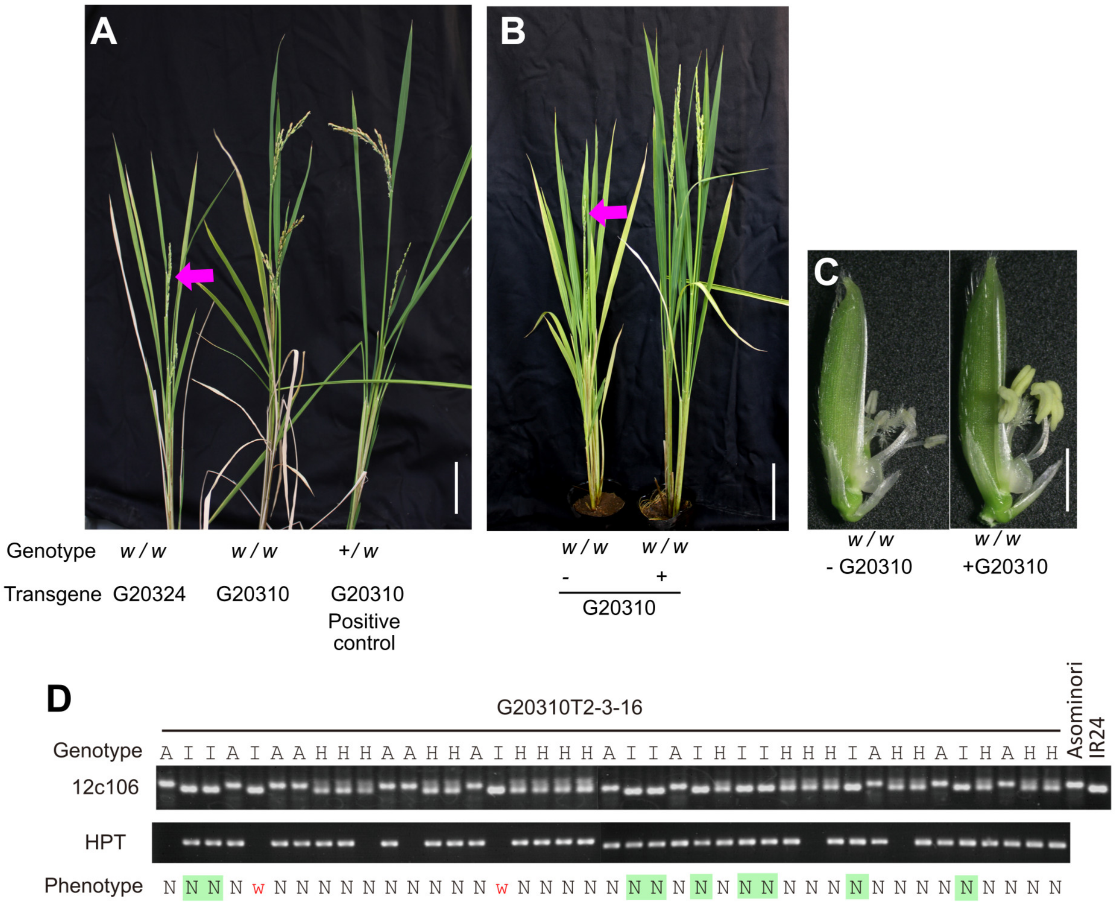

Supplementary Figure 6. Complementation test of hybrid breakdown. (**A**) Photograph of T1 plants transformed with the genome fragment harboring *LOC_Os12g20324* (G20324) or *LOC_Os12g20310* (G20310) at the seed maturation stage. Maturing seeds were observed in the G20310 transformant but not in the G20324 transformant showing the typical weakness phenotype with a sheathed panicle (magenta arrow). (**B**) Plant morphology of T1 plants with or without G20310 transgene. (**C**) Flower morphology of T1 plants with or without G20310 transgene. (**D**) Segregation of weak plants in a selfed progeny of the T1 transplant (T2 generation). Genotype of the DNA marker 12c106 (upper) and phenotype (lower) are shown. Genotype, A: Asominori, I: IR24, H: heterozygous. Phenotype, N: normal, w: weak. Note that IR24 homozygotes for 12c106 lacking hygromycin phosphotransferase (HPT) product had a weak phenotype, whereas those with HPT products recovered with a normal phenotype. Scale bar = 10.0 cm in (**A, B**), and 2.0 mm in (**C**).

```
                      *        20         *        40         *        60         *        80         *       100
ch12_MH6  : TTTTCCCATTGTAATGCACGGGCAACTAACTTGGGTATGGACATTATCTCATGTTTGTGGTCATGATCAAATCGCTCATAAAACATGTGCATCTTAGATT : 100
Unma_KAS  : TTTTCCCATTGTAATGCACGGGCAACTAACTTGGGTATGGACATTATCTCATGTTTGTGGTCATGATCAAATCGCTCATAAAACATGTGCATCTTAGATT : 100
ch12_NIP  : TTTTCCCATTGTAATGCACGGGCAACTAACTAGGGTATGGACATTATCTCATGTTTGTGGTCATGATCAAATCGCTCATAAAACATGTGCATCTTAGATT : 100
ch01-ruf  : TTTTCCCATTGTAATGCACGGGCACCTAACTAGGGTATGGACATTATCTCATGTTTGTGGTCATGATCAAATCGCTCATAAAACATGTGCATCTTAGATT : 100
ch01_MH6  : TTTTCCCATTGTAATGCACGGGCACCTAACTAGGGTATGGACATTATCTCATGTTTGTGGTCATGATCAAATCGCTCATAAAACATGTGCATCTTAGATT : 100
ch01_ZS9  : TTTTCCCATTGTAATGCACGGGCACCTAACTAGGGTATGGACATTATCTCATGTTTGTGGTCATGATCAAATCGCTCATAAAACATGTGCATCTTAGATT : 100
ch01_IR8  : TTTTCCCATTGTAATGCACGGGCACCTAACTAGGGTATGGACATTATCTCATGTTTGTGGTCATGATCAAATCGCTCATAAAACATGTGCATCTTAGATT : 100
ch01_931  : TTTTCCCATTGTAATGCACGGGCACCTAACTAGGGTATGGACATTATCTCATGTTTGTGGTCATGATCAAATCGCTCATAAAACATGTGCATCTTAGATT : 100
ch12_ZS9  : TTTTCCCATTGTAATGCACGGGCACCTAACTAGGGTATGGACATTATCTCATGTTTGTGGTCATGATCAAATCGCTCATAAAACATGTGCATCTTAGATT : 100
ch01-NIP  : TTTTCCCATTGCAACACATGGGCACCTAACTAGGGTATGGACATTATCTCATGTTTGTGGTCATGATCAAATCGCTCATAAAACATGTGCATCTTAGATT : 100
ch01_KAS  : TTTTCCCATTGCAACACATGGGCACCTAACTAGGGTATGGACATTATCTCATGTTTGTGGTCATGATCAAATCGCTCATAAAACATGTGCATCTTAGATT : 100
            TTTTCCCATTGtAAtgCAcGGGCA CTAACTaGGGTATGGACATTATCTCATGTTTGTGGTCATGATCAAATCGCTCATAAAACATGTGCATCTTAGATT

                      *       120         *       140         *       160         *       180         *       200
ch12_MH6  : ATAGGCAGAAATGTATTCCAATAATGTTATTTGGAGTGAAATTCATCCCTCAACAGAAAAAAATT-GTGAAACACAAATTTCTGCTAAATACTCTATTAA : 199
Unma_KAS  : ATAGGCAGAAATGTATTCCAATAATGTTATTTGGAGTGAAATTCATCCCTCAACAGAAAAAAATT-GTGAAACACAAATTTCTGCTAAATACTCTATTAA : 199
ch12_NIP  : ATAGGCAGAAATGTATTCCAATAATGTTATTTGGAGTGAAATTCATCCCTCAACAGAAAAAAA----------------TTCTGCTAAATACTCTATTAA : 184
ch01-ruf  : ATAGGCAGAAATGTATTCCAATAATGTTATTTGGAGTGAAATTCATCCCCCAACAGAAAAAAATT-GTGAAACACAAATTTCTGCTAAATACTCTATTAA : 199
ch01_MH6  : ATAGGCAGAAATGTATTCCAATAATGTTATTTGGAGTGAAATTCATCCCTCAACAGAAAAAAATT-GTGAAACACAAATTTCTGCTAAATACTCTATTAA : 199
ch01_ZS9  : ATAGGCAGAAATGTATTCCAATAATGTTATTTGGAGTGAAATTCATCCCTCAACAGAAAAAAATT-GTGAAACACAAATTTCTGCTAAATACTCTATTAA : 199
ch01_IR8  : ATAGGCAGAAATGTATTCCAATAATGTTATTTGGAGTGAAATTCATCCCTCAACAGAAAAAAATT-GTGAAACACAAATTTCTGCTAAATACTCTATTAA : 199
ch01_931  : ATAGGCAGAAATGTATTCCAATAATGTTATTTGGAGTGAAATTCATCCCTCAACAGAAAAAAATT-GTGAAACACAAATTTCTGCTAAATACTCTATTAA : 199
ch12_ZS9  : ATAGGCAGAAATGTATTCCAATAATGTTATTTGGAGTGAAATTCATCCCTCAACAGAAAAAAATT-GTGAAACACAAATTTCTGCTAAATACTCTATTAA : 199
ch01-NIP  : ATAGGCAGAAATGTATTCCAATAATGTTATTTGGAGTGAAATTTATCCATCAACAGAAAAAAAAA-TTGAAACACAAATTTCTGCTAAATACTCTATTAA : 199
ch01_KAS  : ATAAGCAGAAATGTATTCCAATAATGTTATTTGGAGTGAAATTTATCCATCAACAGAAAAAAAAAAATTGAAACACAAATTTCTGCTAAATACTCTATTAA : 200
            ATAgGCAGAAATGTATTCCAATAATGTTATTTGGAGTGAAATTcATCCctCAACAGAAAAAAA    tgaaacacaaatTTCTGCTAAATACTCTATTAA

                      *       220         *       240         *       260         *       280         *       300
ch12_MH6  : CCTTTTCTGTGTGATACTCATGGGTATGTATGCATTGATTGCTAAACATTTTCACATGTGCATTTGACAGCTCAGGTG------GCAGCAATCAGGAAGC : 293
Unma_KAS  : CCTTTTCTGTGTGATACTCATGGGTATGTATGCATTGATTGCTAAACATTTTCACATGTGCATTTGACAGCTCAGGTG------GCAGCAATCAGGAAGC : 293
ch12_NIP  : CCTTTTCTGTGTGATACTCATGGGTATGTATGCATTGATTGCTAAACATTTTCACATGTGCATTTGACAGCTCAGGTG------GCAGCAAACAGGAAGC : 278
ch01-ruf  : CCTTTTCTGTGTGATACTCATGGGTATGTATGCATTGATTGCTAAACATTTTCACATGTGCATTTGACAGCTCAGGTG------GCAGCAAACAGGAAGC : 293
ch01_MH6  : CCTTTTCTGTGTGATACTCATGGGTATGTATGCATTGATTGCTAAACATTTTCACATGTGCATTTGACAGCTCAGGTG------GCAGCAAACAGGAAGC : 293
ch01_ZS9  : CCTTTTCTGTGTGATACTCATGGGTATGTATGCATTGATTGCTAAACATTTTCACATGTGCATTTGACAGCTCAGGTG------GCAGCAAACAGGAAGC : 293
ch01_IR8  : CCTTTTCTGTGTGATACTCATGGGTATGTATGCATTGATTGCTAAACATTTTCACATGTGCATTTGACAGCTCAGGTG------GCAGCAAACAGGAAGC : 293
ch01_931  : CCTTTTCTGTGTGATACTCATGGGTATGTATGCATTGATTGCTAAACATTTTCACATGTGCATTTGACAGCTCAGGTG------GCAGCAAACAGGAAGC : 293
ch12_ZS9  : CCTTTTCTGTGTGATACTCATGGGTATGTATGCATTGATTGCTAAACATTTTCACATGTGCATTTGACAGCTCAGGTG------GCAGCAAACAGGAAGC : 293
ch01-NIP  : CCTTTTCTGTGTTATACTCATGCGTATGTATGCATTGATTGCTAAACATTTTCACATGTGCATTTGACAGCTTAGGTGTGACTTGCAGCAAACAAGAATC : 299
ch01_KAS  : CCTTTTCTGTGTTATACTCATGCGTATGTATGCATTGATTGCTAAACATTTTCACATGTGCATTTGACAGCTCAGGTGTGACTTGCAGCAAACAAGAATC : 300
            CCTTTTCTGTGTgATACTCATGgGTATGTATGCATTGATTGCTAAACATTTTCACATGTGCATTTGACAGCTcAGGTG      GCAGCAAaCAgGAAgC

                      *       320         *       340         *       360         *       380         *       400
ch12_MH6  : TTTCTCCATTAGAAGGAATCAAACCGATCGAGAAGGATCAAACCAAGAAGAGAAAGGATCCTACATAGTATCTTGATACTGGTTACCTTGGCTAAGAGGA : 393
Unma_KAS  : TTTCTCCATTAGAAGGAATCAAACCGATCGAGAAGGATCAAACCAAGAAGAGAAAGGATCCTACATAGTATCTTGATACTGGTTACCTTGGCTAAGAGGA : 393
ch12_NIP  : TTTCTCCATTAGAAGGAATCAAACCGATCGAGAAGGATCAAACCAAGAAGAGAAAGGATCCTACATAGTATCTTGATACTGGTTACCTTGGCTAAGAGGA : 378
ch01-ruf  : TTTCTCCATTAGAAGGAATCAAACCGATCGAGAAGGATCAAACCA-GAAGAGAAAGGATCCTACATAGTATCTTGATACTGGTTACCTTGGCTAAGAGGA : 392
ch01_MH6  : TTTCTCCATTAGAAGGAATCAAACCGATCGAGAAGGATCAAACCAAGAAGAGAAAGGATCCTACATAGTATCTTGATACTGGTTACCTTGGCTAAGAGGA : 393
ch01_ZS9  : TTTCTCCATTAGAAGGAATCAAACCGATCGAGAAGGATCAAACCAAGAAGAGAAAGGATCCTACATAGTATCTTGATACTGGTTACCTTGGCTAAGAGGA : 393
ch01_IR8  : TTTCTCCATTAGAAGGAATCAAACCGATCGAGAAGGATCAAACCAAGAAGAGAAAGGATCCTACATAGTATCTTGATACTGGTTACCTTGGCTAAGAGGA : 393
ch01_931  : TTTCTCCATTAGAAGGAATCAAACCGATCGAGAAGGATCAAACCAAGAAGAGAAAGGATCCTACATAGTATCTTGATACTGGTTACCTTGGCTAAGAGGA : 393
ch12_ZS9  : TTTCTCCATTAGAAGGAATCAAACCGATCGAGAAGGATCAAACCAAGAAGAGAAAGGATCCTACATAGTATCTTGATACTGGTTACCTTGGCTAAGAGGA : 393
ch01-NIP  : TTTCTCCATTAGAAGGGATCAAACCGATCGAGAAGGATCAAATCAAGAAGAGAAAGGATCCTACCTAGTATCTCGATATTGATTACCTTGGCTAAGAGGA : 399
ch01_KAS  : TTTCTCCATTAGAAGGGATCAAACCGATCGAGAAGGATCAAATCAAGAAGAGAAAGGATCCTACATAGTATCTCGATATTGATTACCTTGGCTAAGAGGA : 400
            TTTCTCCATTAGAAGGaATCAAACCGATCGAGAAGGATCAAAcCAaGAAGAGAAAGGATCCTACaTAGTATCTtGATAcTGgTTACCTTGGCTAAGAGGA

                      *       420         *       440         *       460         *       480         *       500
ch12_MH6  : CCGATCGGACTATTGGAGAGGAGGGGCTAAGTCGTCTCCACAACAGTGAACTGTCTCCGAGCCAAAACAGAACGAACGCCCAAACAGGCAAACCACGAAC : 493
Unma_KAS  : CCGATCGGACTATTGGAGAGGAGGGGCTAAGTCGTCTCCACAACAGTGAACTGTCTCCGAGCCAAAACAGAACGAACGCCCAAACAGGCAAACCACGAAC : 493
ch12_NIP  : CCGATCGTACTATTGGAGAGGAGGGGCTAAGTCGTCTCCACAACAGTGAACTGTCTCCGAGCCAAAACAGAACGAACGCCCAAACAGGCAAACCACGAAC : 478
ch01-ruf  : CCGATCGGACTATTGGAGAGGAGGGGCTAAGTCGTCTCCACAACAGTGAACTGTCTCCGAGCCAAAACAGAACGAACGCCCAAACAGGCAAACCACGAAC : 492
ch01_MH6  : CCGATCGGACTATTGGAGAGGAGGGGCTAAGTCGTCTCCACAACAGTGAACTGTCTCCGAGCCAAAACAGAACGAACGCCCAAACAGGCAAACCACGAAC : 493
ch01_ZS9  : CCGATCGGACTATTGGAGAGGAGGGGCTAAGTCGTCTCCACAACAGTGAACTGTCTCCGAGCCAAAACAGAACGAACGCCCAAACAGGCAAACCACGAAC : 493
ch01_IR8  : CCGATCGGACTATTGGAGAGGAGGGGCTAAGTCGTCTCCACAACAGTGAACTGTCTCCGAGCCAAAACAGAACGAACGCCCAAACAGGCAAACCACGAAC : 493
ch01_931  : CCGATCGGACTATTGGAGAGGAGGGGCTAAGTCGTCTCCACAACAGTGAACTGTCTCCGAGCCAAAACAGAACGAACGCCCAAACAGGCAAACCACGAAC : 493
ch12_ZS9  : CCGATCGGACTATTGGAGAGGAGGGGCTAAGTCGTCTCCACAACAGTGAACTGTCTCCGAGCCAAAACAGAACGAACGCCCAAACAGGCAAACCACGAAC : 493
ch01-NIP  : CCGATCGGATTATTGGAGAGGAGGGGCTA-GTCGTCTCCACAACAGTGAACTGTGTCCGAGCCAAAACAGAACGAACGCCCAAACAGGCAAACCACGAAC : 498
ch01_KAS  : CCGATCGGATTATTGGAGAGGAGGGGCTA-GTCGTCTCCACAACAGTGAACTGTGTCCGAGCCAAAACAGAACGAACGCCCAAACAGGCAAACCACGAAC : 499
            CCGATCGgAcTATTGGAGAGGAGGGGCTAaGTCGTCTCCACAACAGTGAACTGTcTCCGAGCCAAAACAGAACGAACGCCCAAACAGGCAAACCACGAAC

                      *       520         *       540         *       560         *       580         *       600
ch12_MH6  : TCGAACCCTAGAAACCCTGACCATGGACTCGGGAGGCGGAGGAGGGGCATCGCACAAGGCAGCGTCGGGCTCCGCACCCTCGGGGGCGGCGGCCGCGAAC : 593
Unma_KAS  : TCGAACCCTAGAAACCCTGACCATGGACTCGGGAGGCGGAGGAGGGGCATCGCACAAGGCAGCGTCGGGCTCCGCACCCTCGGGGGCGGCGGCCGCGAAC : 593
ch12_NIP  : TCGAACCCTAGAAACCCTGACCATGGACTCGGGAGGCGGAGGAGGGGCATCGCACAAGGCAGCGTCGGGCTCTGCACCCTCGGGGGCGGCGGCCGCGAAC : 578
ch01-ruf  : TCGAACCCTAGAAACCCTGACCATGGACTCGGGAGGCGGAGGAGGGGCATCGCACAAGGCAGCGTCGGGCTCCGCACCCTCGGGGGCGGCGGCCGCGAAC : 592
ch01_MH6  : TCGAACCCTAGAAACCCTGACCATGGACTCGGGAGGCGGAGGAGGGGCATCGCACAAGGCAGCGTCGGGCTCCGCACCCTCGGGGGCGGCGGCCGCGAAC : 593
ch01_ZS9  : TCGAACCCTAGAAACCCTGACCATGGACTCGGGAGGCGGAGGAGGGGCATCGCACAAGGCAGCGTCGGGCTCCGCACCCTCGGGGGCGGCGGCCGCGAAC : 593
ch01_IR8  : TCGAACCCTAGAAACCCTGACCATGGACTCGGGAGGCGGAGGAGGGGCATCGCACAAGGCAGCGTCGGGCTCCGCACCCTCGGGGGCGGCGGCCGCGAAC : 593
ch01_931  : TCGAACCCTAGAAACCCTGACCATGGACTCGGGAGGCGGAGGAGGGGCATCGCACAAGGCAGCGTCGGGCTCCGCACCCTCGGGGGCGGCGGCCGCGAAC : 593
ch12_ZS9  : TCGAACCCTAGAAACCCTGACCATGGACTCGGGAGGCGGAGGAGGGGCATCGCACAAGGCAGCGTCGGGCTCCGCACCCTCGGGGGCGGCGGCCGCGAAC : 593
ch01-NIP  : TCGAACCCTAGAAACCCTAACCATGGACTCGGGAGGCGGAGGAGGGGCATCGCACAAGGCAGCGTCAGGCTCTGCGCCCTCGGGGGCGGCGGCCGCGAAC : 598
ch01_KAS  : TCGAACCCTAGAAACCCTAACCATGGACTCGGGAGGCGGAGGAGGGGCATCGCACAAGGCAGCGTCAGGCTCTGCGCCCTCGGGGGCGGCGGCCGCGAAC : 599
            TCGAACCCTAGAAACCCTgACCATGGACTCGGGAGGCGGAGGAGGGGCATCGCACAAGGCAGCGTCgGGCTC GCaCCCTCGGGGGCGGCGGCCGCGAAC

                      *       620         *       640         *       660         *       680         *       700
ch12_MH6  : CCCACGGCGATGCTCTCCGCCCTGATGAGCAAGCGCGCCAAGCTCCAGGAGGAGCTCCGCTCCATAGAGCGCCAGGTTAGGTCTTACACACTGTTCCCTG : 693
Unma_KAS  : CCCACGGCGATGCTCTCCGCCCTGATGAGCAAGCGCGCCAAGCTCCAGGAGGAGCTCCGCTCCATAGAGCGCCAGGTTAGGTCTTACACACTGTTCCCTG : 693
ch12_NIP  : CCCACGGCGATGCTCTCCGCCCTGATGAGCAAGCGCGCCAAGCTCCAGGAGGAGCTCCGCTCCATAGAGCGCCAGGTTAGGTCTTACACACTGTTCCCTG : 678
ch01-ruf  : CCCACGGCGATGCTCTCCGCCCTGATGAGCAAGCGCGCCAAGCTCCAGGAGGAGCTCCGCTCCATAGAGCGCCAGGTTAGGTCTTACACACTGTTCCCTG : 692
ch01_MH6  : CCCACGGCGATGCTCTCCGCCCTGATGAGCAAGCGTGCCAAGCTCCAGGAGGAGCTCCGCTCCATAGAGCGCCAGGTTAGGTCTTACACACTGTTCCCTG : 693
ch01_ZS9  : CCCACGGCGATGCTCTCCGCCCTGATGAGCAAGCGTGCCAAGCTCCAGGAGGAGCTCCGCTCCATAGAGCGCCAGGTTAGGTCTTACACACTGTTCCCTG : 693
ch01_IR8  : CCCACGGCGATGCTCTCCGCCCTGATGAGCAAGCGTGCCAAGCTCCAGGAGGAGCTCCGCTCCATAGAGCGCCAGGTTAGGTCTTACACACTGTTCCCTG : 693
ch01_931  : CCCACGGCGATGCTCTCCGCCCTGATGAGCAAGCGCGCCAAGCTCCAGGAGGAGCTCCGCTCCATAGAGCGCCAGGTTAGGTCTTACACACTGTTCCCTG : 693
```

```
ch12_ZS9 : CCCACGGCGATGCTCTCCGCCCTGATGAGCAAGCGCGCCAAGCTCCAGGAGGAGCTCCGCTCCATAGAGCGCCAGGTTAGGTCTTACACACTGTTCCCTG :  693
ch01-NIP : CCCACAGCGATGCTCTCCGCCCTGATGAGCAAGCGCGCCAAGCTCCAGGAGGAGCTCCGCTCCATAGAGCGCCAGGTTAGGTCTTACACACAGTCCCC-- :  696
ch01_KAS : CCCACAGCGATGCTCTCCGCCCTGATGAGCAAGCGCGCCAAGCTCCAGGAGGAGCTCCGCTCCATAGAGCGCCAGGTTAGGTCTTACACACAGTCCCC-- :  697
           CCCACgGCGATGCTCTCCGCCCTGATGAGCAAGCG GCCAAGCTCCAGGAGGAGCTCCGCTCCATAGAGCGCCAGGTTAGGTCTTACACACtGTtCCCtg

                *      720         *       740         *       760         *       780         *       800
ch12_MH6 : TCTCTTTTGCGCCTCTCTCTCTCTCTCTCT--TGGAATTAGGGTTTTGTATAGGAGACCATGGATTTGTTGCATATATGTTCAGATGGCAAGGGCTTGCC :  791
Unma_KAS : TCTCTTTTGCGCCTCTCTCTCTCTCTCTCT--TGGAATTAGGGTTTTGTATAGGAGACCATGGATTTGTTGCATATATGTTCAGATGGCAAGGGCTTGCC :  791
ch12_NIP : TCTCTTTTGCGCCTCTCTCTCTCTCTCTCT--TGGAATTAGGGTTTTGTATAGGAGACCATGGATTTGTTGCATATATGTTCAGATGGCAAGGGCTTGCC :  776
ch01-ruf : TCTCTTTTGCGCCTCTCTCTCTCTCTCTCTCTTGGAATTAGGGTTTTGTATAGGAGACCATGGATTTGTTGCATATATGTTCAGATGGCAAGGGCTTGCC :  792
ch01_MH6 : TCTCTTTTGCGCCTCTCTCTCTCTCTCT----TGGAATTAGGGTTTTGTATAGGAGACCATGGATTTGTTGCATATATGTTCAGATGGCAAGGGCTTGCC :  789
ch01_ZS9 : TCTCTTTTGCGCCTCTCTCTCTCTCTCT----TGGAATTAGGGTTTTGTATAGGAGACCATGGATTTGTTGCATATATGTTCAGATGGCAAGGGCTTGCC :  789
ch01_IR8 : TCTCTTTTGCGCCTCTCTCTCTCTCTCT----TGGAATTAGGGTTTTGTATAGGAGACCATGGATTTGTTGCATATATGTTCAGATGGCAAGGGCTTGCC :  789
ch01_931 : TCTCTTTTGCGCCTCTCTCTCTCTCTCT----TGGAATTAGGGTTTTGTATAGGAGACCATGGATTTGTTGCATATATGTTCAGATGGCAAGGGCTTGCC :  789
ch12_ZS9 : TCTCTTTTGCGCCTCTCTCTCTCTCTCTCT--TGGAATTAGGGTTTTGTATAGGAGACCATGGATTTGTTGCATATATGTTCAGATGGCAAGGGCTTGCC :  791
ch01-NIP : ---------------------------------------------------------------------------------------------------- :    -
ch01_KAS : ---------------------------------------------------------------------------------------------------- :    -
           tctcttttgcgcctctctctctctctct    tggaattagggttttgtataggagaccatggatttgttgcatatatgttcagatggcaagggcttgcc

                *      820         *       840         *       860         *       880         *       900
ch12_MH6 : ATTGCTCAACATGCGAATGAGTGGTGCGAATTTACATTGCTGCGGATGGTTGTAAAATTGGAGTTGTGGATACCTGTAGTAGCCTGAATTTATTGTTCAA :  891
Unma_KAS : ATTGCTCAACATGCGAATGAGTGGTGCGAATTTACATTGCTGCGGATGGTTGTAAAATTGGAGTTGTGGATACCTGTAGTAGCCTGAATTTATTGTTCAA :  891
ch12_NIP : ATTGCTCAACATGCGAATGAGTGGTGCGAATTTACATTGCTGCGGATGGTTGTAAAATTGGAGTTGTGGATACCTGTAGTAGCCTGAATTTATTGTTCAA :  876
ch01-ruf : ATTGCTCAACATGCGAATGAGTGGTGCGAATTTACATTGCTGCGGATGGTTGTAAAATTGGAGTTGTGGATACCTGTAGTAGCCTGAATTTATTGTTCAA :  892
ch01_MH6 : ATTGCTCAACATGCGAATGAGTGGTGCGAATTTACATTGCTGCGGATGGTTGTAAAATTGGAGTTGTGGATACCTGTAGTAGCCTGAATTTATTGTTCAA :  889
ch01_ZS9 : ATTGCTCAACATGCGAATGAGTGGTGCGAATTTACATTGCTGCGGATGGTTGTAAAATTGGAGTTGTGGATACCTGTAGTAGCCTGAATTTATTGTTCAA :  889
ch01_IR8 : ATTGCTCAACATGCGAATGAGTGGTGCGAATTTACATTGCTGCGGATGGTTGTAAAATTGGAGTTGTGGATACCTGTAGTAGCCTGAATTTATTGTTCAA :  889
ch01_931 : ATTGCTCAACATGCGAATGAGTGGTGCGAATTTACATTGCTGCGGATGGTTGTAAAATTGGAGTTGTGGATACCTGTAGTAGCCTGAATTTATTGTTCAA :  889
ch12_ZS9 : ATTGCTCAACATGCGAATGAGTGGTGCGAATTTACATTGCTGCGGATGGTTGTAAAATTGGAGTTGTGGATACCTGTAGTAGCCTGAATTTATTGTTCAA :  891
ch01-NIP : --------------------------------------------------------------------------------------AATTTATTGTGCAA :  710
ch01_KAS : --------------------------------------------------------------------------------------AATTTATTGTGCAA :  711
           attgctcaacatgcgaatgagtggtgcgaatttacattgctgcggatggttgtaaaattggagttgtggatacctgtagtagcctgAATTTATTGTtCAA

                *      920         *       940         *       960         *       980         *      1000
ch12_MH6 : TCTTTCTTAATGCCTTGGGAATGATAGAAATATGAACCTATTTGGAACCAATATGCTGGACCAGTGACCACTGATTGTCATTTCACAGAGAGGATCAGTT :  991
Unma_KAS : TCTTTCTTAATGCCTTGGGAATGATAGAAATATGAACCTATTTGGAACCAATATGCTGGACCAGTGACCACTGATTGTCATTTCACAGAGAGGATCAGTT :  991
ch12_NIP : TCTTTCTTAATGCCTTGGGAATGATAGAAATATGAACCTATTTGGAACCAATATGCTGGACCAGTGACCACTGATTGTCATTTCACAGAGAGGATCAGTT :  976
ch01-ruf : TCTTTCTTAATGCCTTGGGAATGATAGAAATATGAACCTATTTGGAACCAATATGCTGGACCAGTGACCACTGATTGTCATTTCACAGAGAGGATCAGTT :  992
ch01_MH6 : TCTTTCTTAATGCCTTGGGAATGATAGAAATATGAACCTATTTGGAACCAATATGCTGGACCAGTGACCACTGATTGTCATTTCACAGAGAGGATCAGTT :  989
ch01_ZS9 : TCTTTCTTAATGCCTTGGGAATGATAGAAATATGAACCTATTTGGAACCAATATGCTGGACCAGTGACCACTGATTGTCATTTCACAGAGAGGATCAGTT :  989
ch01_IR8 : TCTTTCTTAATGCCTTGGGAATGATAGAAATATGAACCTATTTGGAACCAATATGCTGGACCAGTGACCACTGATTGTCATTTCACAGAGAGGATCAGTT :  989
ch01_931 : TCTTTCTTAATGCCTTGGGAATGATAGAAATATGAACCTATTTGGAACCAATATGCTGGACCAGTGACCACTGATTGTCATTTCACAGAGAGGATCAGTT :  989
ch12_ZS9 : TCTTTCTTAATGCCTTGGGAATGATAGAAATATGAACCTATTTGGAACCAATATGCTGGACCAGTGACCACTGATTGTCATTTCACAGAGAGGATCAGTT :  991
ch01-NIP : TCTTTCTTAATGCCTTGGGAATGATAGAAATATGAACCTATTTGGAACCAATATGCTGGACCAGTGACCACTGATTGTCATTTCACAGAGAGGATCAATT :  810
ch01_KAS : TCTTTCTTAATGCCTTGGGAATGATAGAAATATGAACCTATTTGGAACCAATATGCTGGACCAGTGACCACTGATTGTCATTTCACAGAGAGGATCAGTT :  811
           TCTTTCTTAATGCCTTGGGAATGATAGAAATATGAACCTATTTGGAACCAATATGCTGGACCAGTGACCACTGATTGTCATTTCACAGAGAGGATCAgTT

                *     1020         *      1040         *      1060         *      1080         *      1100
ch12_MH6 : GTGACGAACAATTTGGATAGAATTAGGAGGAAGGGAAGAAAGTATCAATTTGATATTCTTGTCGAATATGACCATTCTATTTCTTGTATGTTGTTCTTTG : 1091
Unma_KAS : GTGACGAACAATTTGGATAGAATTAGGAGGAAGGGAAGAAAGTATCAATTTGATATTCTTGTCGAATATGACCATTCTATTTCTTGTATGTTGTTCTTTG : 1091
ch12_NIP : GTGACGAACAATTTGGATAGAATTAGGAGGAAGGGAAGAAAGTATCAATTTGATATTCTTGTCGAATATGACCATTCTATTTCTTGTATGTTGTTCTTTG : 1076
ch01-ruf : GTGACGAACAATTTGGATAGAATTAGGAGGAAGGGAAGAAAGTATCAATTTGATATTCTTGTCGAATATGACCATTCTATTTCTTGTATGTTGTTCTTTG : 1092
ch01_MH6 : GTGACGAACAATTTGGATAGAATTAGGAGGAAGGGAAGAAAGTATCAATTTGATATTCTTGTCGAATATGACCATTCTATTTCTTGTATGTTGTTCTTTG : 1089
ch01_ZS9 : GTGACGAACAATTTGGATAGAATTAGGAGGAAGGGAAGAAAGTATCAATTTGATATTCTTGTCGAATATGACCATTCTATTTCTTGTATGTTGTTCTTTG : 1089
ch01_IR8 : GTGACGAACAATTTGGATAGAATTAGGAGGAAGGGAAGAAAGTATCAATTTGATATTCTTGTCGAATATGACCATTCTATTTCTTGTATGTTGTTCTTTG : 1089
ch01_931 : GTGACGAACAATTTGGATAGAATTAGGAGGAAGGGAAGAAAGTATCAATTTGATATTCTTGTCGAATATGACCATTCTATTTCTTGTATGTTGTTCTTTG : 1089
ch12_ZS9 : GTGACGAACAATTTGGATAGAATTAGGAGGAAGGGAAGAAAGTATCAATTTGATATTCTTGTCGAATATGACCATTCTATTTCTTGTATGTTGTTCTTTG : 1091
ch01-NIP : GTGACGAACAATTTGGATAGAATTAGGAGGAAGGGAAGAAAGTATCAATTTGATATTCTTGTCGAATATGACCATTCTATTTCTTGTATGTTGTTCTTTG :  910
ch01_KAS : GTGACGAACAATTTGGATAGAATTAGGAGGAAGGGAAGAAAGTATCAATTTGATATTCTTGTCGAATATGACCATTCTATTTCTTGTATGTTGTTCTTTG :  911
           GTGACGAACAATTTGGATAGAATTAGGAGGAAGGGAAGAAAGTATCAATTTGATATTCTTGTCGAATATGACCATTCTATTTCTTGTATGTTGTTCTTTG

                *     1120         *      1140         *      1160         *      1180         *      1200
ch12_MH6 : TGCCTGAGAACACTTTGTTATTCTAAGGTAAAAAAGAAAATCAGTTAGCTACAACCTGAAAATTCCATCTGTTCTTCTATCCTCGAGTTAAAATGTGGAG : 1191
Unma_KAS : TGCCTGAGAACACTTTGTTATTCTAAGGTAAAAAAGAAAATCAGTTAGCTACAACCTGAAAATTCCATCTGTTCTTCTATCCTCGAGTTAAAATGTGGAG : 1191
ch12_NIP : TGCCTGAGAACACTTTGTTATTCTAAGGTAAAAAAGAAAATCAGTTAGCTACAACCTGAAAATTCCATCTGTTCTTCTATCCTCGAGTTAAAATGTGGAG : 1176
ch01-ruf : TGCCTGAGAACACTTTGTTATTCTAAGGTAAAAAAGAAAATCAGTTAGCTACAACCTGAAAATTCCATCTGTTCTTCTATCCTCGAGTTAAAATGTGGAG : 1192
ch01_MH6 : TGCCTGAGAACACTTTGTTATTCTAAGGTAAAAAAGAAAATCAGTTAGCTACAACCTGAAAATTCCATCTGTTCTTCTATCCTCGAGTTAAAATGTGGAG : 1189
ch01_ZS9 : TGCCTGAGAACACTTTGTTATTCTAAGGTAAAAAAGAAAATCAGTTAGCTACAACCTGAAAATTCCATCTGTTCTTCTATCCTCGAGTTAAAATGTGGAG : 1189
ch01_IR8 : TGCCTGAGAACACTTTGTTATTCTAAGGTAAAAAAGAAAATCAGTTAGCTACAACCTGAAAATTCCATCTGTTCTTCTATCCTCGAGTTAAAATGTGGAG : 1189
ch01_931 : TGCCTGAGAACACTTTGTTATTCTAAGGTAAAAAAGAAAATCAGTTAGCTACAACCTGAAAATTCCATCTGTTCTTCTATCCTCGAGTTAAAATGTGGAG : 1189
ch12_ZS9 : TGCCTGAGAACACTTTGTTATTCTAAGGTAAAAAAGAAAATCAGTTAGCTACAACCTGAAAATTCCATCTGTTCTTCTATCCTCGAGTTAAAATGTGGAG : 1191
ch01-NIP : TGCCTGAGAACACTTTGTTGCTCTAGGGTAAAAATGAAAATCAGTTAGCTACAACCTGTAAATTCCATCTGTTCTT--ATTATCAAGATAAGATGTGGAG : 1008
ch01_KAS : TGCCTGAGAACACTTTGTTGCTCTAGGGTAAAAATGAAAATCAGTTAGCTACAACCTGTAAATTCCATCTGTTCTT--ATTATCAAGATAAGATGTGGAG : 1009
           TGCCTGAGAACACTTTGTTatTCTAaGGTAAAAAaGAAAATCAGTTAGCTACAACCTGaAAATTCCATCTGTTCTTctATccTCgAGtTAAaATGTGGAG

                *     1220         *      1240         *      1260         *      1280         *      1300
ch12_MH6 : TTTCTACTGTGTCTTAGTCAGAAATTATTCGCAGCAAGTATCAAATGGGCTACTTCTATTCATAGTATTTCAAAGTTACACTATGTGGTCCAAATTCCAA : 1291
Unma_KAS : TTTCTACTGTGTCTTAGTCAGAAATTATTCGCAGCAAGTATCAAATGGGCTACTTCTATTCATAGTATTTCAAAGTTACACTATGTGGTCCAAATTCCAA : 1291
ch12_NIP : TTTCTACTGTGTCTTAGTCAGAAATTATTCGCAGCAAGTATCAAATGAGCTACTTCTATTCATAGTATTTCAAAGTTACACTATGTGGTCCAAATTCCAA : 1276
ch01-ruf : TTTCTACTGTGTCTTAGTCAGAAATTATTCGCAGCAAGTATCAAATGAGCTACTTCTATTCATAGTATTTCAAAGTTACACTATGTGGTCCAAATTCCAA : 1292
ch01_MH6 : TTTCTACTGTGTCTTAGTCAGAAATTATTCGCAGCAAGTATCAAATGAGCTACTTCTATTCATAGTATTTCAAAGTTACACTATGTGGTCCAAATTCCAA : 1289
ch01_ZS9 : TTTCTACTGTGTCTTAGTCAGAAATTATTCGCAGCAAGTATCAAATGAGCTACTTCTATTCATAGTATTTCAAAGTTACACTATGTGGTCCAAATTCCAA : 1289
ch01_IR8 : TTTCTACTGTGTCTTAGTCAGAAATTATTCGCAGCAAGTATCAAATGAGCTACTTCTATTCATAGTATTTCAAAGTTACACTATGTGGTCCAAATTCCAA : 1289
ch01_931 : TTTCTACTGTGTCTTAGTCAGAAATTATTCGCAGCAAGTATCAAATGAGCTACTTCTATTCATAGTATTTCAAAGTTACACTATGTGGTCCAAATTCCAA : 1289
ch12_ZS9 : TTTCTACTGTGTCTTAGTCAGAAATTATTCGCAGCAAGTATCAAATGAGCTACTTCTATTCATAGTATTTCAAAGTTACACTATGTGGTCCAAATTCCAA : 1291
ch01-NIP : TTTCTACTGTGTCTTAGTCAGAAATTATTTGCAACAAGTATCAAATGAGCTACTTCTATTCAAAGTATTTCAAAGTTACACTATGTGCTCCAACTTCCAA : 1108
ch01_KAS : TTTCTACTGTGTCTTAGTCAGAAATTATTTGCAACAAGTATCAAATGAGCTACTTCTATTCAAAGTATTTCAAAGTTACACTATGTGCTCCAACTTCCAA : 1109
           TTTCTACTGTGTCTTAGTCAGAAATTATTcGCAgCAAGTATCAAATGaGCTACTTCTATTCAtAGTATTTCAAAGTTACACTATGTGgTCCAAaTTCCAA

                *     1320         *      1340         *      1360         *      1380         *      1400
ch12_MH6 : TATATGTTTGTGATGGTGAATCTTGGTGTGTCATCCAAAATCATTAAATAAGAGGAGCGTTTGGTTTAGTAGAAACCATAATCCTAAAAAGGAGGAAAG : 1391
Unma_KAS : TATATGTTTGTGATGGTGAATCTTGGTGTGTCATCCAAAATCATTAAATAAGAGGAGCGTTTGGTTTAGTAGAAACCATAATCCTAAAAAGGAGGAAAG : 1391
ch12_NIP : TATATGTTTGTGATGGTGAATCTTGGTGTGTCATCCAAAATCATTAAATAAGAGGAGCGTTTGGTTTAGTAGAAACCATAATCCTAAAAAGGAGGAAAG : 1376
ch01-ruf : TATATGTTTGTGATGGTGAATCTTGGTGTGTCATCCAAAATCATTAAATAAGAGGAGCGTTTGGTTTAGTAGAAACCATAATCCTAAAAAGGAGGAAAG : 1392
```

```
ch01_MH6 : TATATGTTTGTGATGGTGAATCTTGGTGTGTCATCCAAAATCATTAAATAAGAGGAGCGTTTGGTTTAGTAGAAACCATAATCCTAAAAAGGGAGGAAAG : 1389
ch01_ZS9 : TATATGTTTGTGATGGTGAATCTTGGTGTGTCATCCAAAATCATTAAATAAGAGGAGCGTTTGGTTTAGTAGAAACCATAATCCTAAAAAGGGAGGAAAG : 1389
ch01_IR8 : TATATGTTTGTGATGGTGAATCTTGGTGTGTCATCCAAAATCATTAAATAAGAGGAGCGTTTGGTTTAGTAGAAACCATAATCCTAAAAAGGGAGGAAAG : 1389
ch01_931 : TATATGTTTGTGATGGTGAATCTTGGTGTGTCATCCAAAATCATTAAATAAGAGGAGCGTTTGGTTTAGTAGAAACCATAATCCTAAAAAGGGAGGAAAG : 1389
ch12_ZS9 : TATATGTTTGTGATGGTGAATCTTGGTGTGTCATCCAAAATCATTAAATAAGAGGAGCGTTTGGTTTAGTAGAAACCATAATCCTAAAAAGGGAGGAAAG : 1391
ch01-NIP : TCTATGTTTGTGATGGTGAATCTTGGTGTGTCATCCAAAATCATTAAATAAGAGGAGCATTTGGTTTAGTAGAAACCATAATCCTAAAAAGGGAGGAAAT : 1208
ch01_KAS : TCTATGTTTGTGATGGTGAATCTTGGTGTGTCATCCAAAATCATTAAATAAGAGGAGCATTTGGTTTAGTAGAAACCATAATCCTAAAAAGGGAGGAAAT : 1209
           TaTATGTTTGTGATGGTGAATCTTGGTGTGTCATCCAAAATCATTAAATAAGAGGAGCgTTTGGTTTAGTAGAAACCATAATCCTAAAAAGGGAGGAAAg

                  *      1420         *      1440         *      1460         *      1480         *      1500
ch12_MH6 : TGCTATTGCTCTCATATCAAGTATTTTAATGGCACTGATATGTGTGTTTTTTTTAGAAAGAACAAAACAGTCGCTCTTTAATACCAGGAACAACACATGA : 1491
Unma_KAS : TGCTATTGCTCTCATATCAAGTATTTTAATGGCACTGATATGTGTGTTTTTTTTAGAAAGAACAAAACAGTCGCTCTTTAATACCAGGAACAACACATGA : 1491
ch12_NIP : TGCTATTGCTCTCATATCAAGTATTTTAATGGCACTGATATGTGTGTTTTTTTTAGAAAGAACAAAACAGTCGCTCTTTAATACCAGGAACAACACATGA : 1476
ch01-ruf : TGCTATTGCTCTCATATCAAGTATTTTAATGGCACTGATATGTGTGTTTTTTTTAGAAAGAACAAAACAGTCGCTCTTTAATACCAGGAACAACACATGA : 1492
ch01_MH6 : TGCTATTGCTCTCATATCAAGTATTTTAATGGCACTGATATGTGTGTTTTTTTTAGAAAGAACAAAACAGTCGCTCTTTAATACCAGGAACAACACATGA : 1489
ch01_ZS9 : TGCTATTGCTCTCATATCAAGTATTTTAATGGCACTGATATGTGTGTTTTTTTTAGAAAGAACAAAACAGTCGCTCTTTAATACCAGGAACAACACATGA : 1489
ch01_IR8 : TGCTATTGCTCTCATATCAAGTATTTTAATGGCACTGATATGTGTGTTTTTTTTAGAAAGAACAAAACAGTCGCTCTTTAATACCAGGAACAACACATGA : 1489
ch01_931 : TGCTATTGCTCTCATATCAAGTATTTTAATGGCACTGATATGTGTGTTTTTTTTAGAAAGAACAAAACAGTCGCTCTTTAATACCAGGAACAACACATGA : 1489
ch12_ZS9 : TGCTATTGCTCTCATATCAAGTATTTTAATGGCACTGATATGTGTGTTTTTTTTAGAAAGAACAAAACAGTCGCTCTTTAATACCAGGAACAACACATGA : 1491
ch01-NIP : GGGTATTGCTCTCATATCAAGTATTTTAATGGCACAGATATGTGT-TTTTTTTTAGAAAGAACAAAACAGTCGCTCTTTAATACCAGGAACAACACATGA : 1307
ch01_KAS : GGGTATTGCTCTCATATCAAGTATTTTAATGGCACAGATATGTGT-TTTTTT--AGAAAGAACAAAACAGTCGCTCTTTAATACCAGGAACAACACATGA : 1306
           tGcTATTGCTCTCATATCAAGTATTTTAATGGCACtGATATGTGTgTTTTTTttAGAAAGAACAAAACAGTCGCTCTTTAATACCAGGAACAACACATGA

                  *      1520         *      1540         *      1560         *      1580         *      1600
ch12_MH6 : TCTATAATGTCAACCATCTTTGCTTGACTTTAAGGGGAAGTTTTGTATATGATATTAAACACTCATAATTTAACAAAATATCTATTCATGCTTTTAACAG : 1591
Unma_KAS : TCTATAATGTCAACCATCTTTGCTTGACTTTAAGGGGAAGTTTTGTATATGATATTAAACACTCATAATTTAACAAAATATCTATTCATGCTTTTAACAG : 1591
ch12_NIP : TCTATAATGTCAACCATCTTTGCTTGACTTTAAGGGGAAGTTTTGTATATGATATTAAACACTCATAATTTAACAAAATATCTATTCATGCTTTTAACAG : 1576
ch01-ruf : TCTATAATGTCAACCATCTTTGCTTGACTTTAAGGGGAAGTTTTGTATATGATATTAAACACTCATAATTTAACAAAATATCTATTCATGCTTTTAACAG : 1592
ch01_MH6 : TCTATAATGTCAACCATCTTTGCTTGACTTTAAGGGGAAGTTTTGTATATGATATTAAACACTCATAATTTAACAAAATATCTATTCATGCTTTTAACAG : 1589
ch01_ZS9 : TCTATAATGTCAACCATCTTTGCTTGACTTTAAGGGGAAGTTTTGTATATGATATTAAACACTCATAATTTAACAAAATATCTATTCATGCTTTTAACAG : 1589
ch01_IR8 : TCTATAATGTCAACCATCTTTGCTTGACTTTAAGGGGAAGTTTTGTATATGATATTAAACACTCATAATTTAACAAAATATCTATTCATGCTTTTAACAG : 1589
ch01_931 : TCTATAATGTCAACCATCTTTGCTTGACTTTAAGGGGAAGTTTTGTATATGATATTAAACACTCATAATTTAACAAAATATCTATTCATGCTTTTAACAG : 1589
ch12_ZS9 : TCTATAATGTCAACCATCTTTGCTTGACTTTAAGGGGAAGTTTTGTATATGATATTAAACACTCATAATTTAACAAAATATCTATTCATGCTTTTAACAG : 1591
ch01-NIP : TCTATAATGTCAACCATCTTTGCTTGACTTTAAGGGGAAGTTTTGTATATGATATTAAACACTCATAATTTAACAAAATATCTATTCATGCTTTTAACAG : 1407
ch01_KAS : TCTATAATGTCAACCATCTTTGCTTGACTTTAAGGGGAAGTTTTGTATATGATATTAAACACTCATAATTTAACAAAATATCTATTCATGCTTTTAACAG : 1406
           TCTATAATGTCAACCATCTTTGCTTGACTTTAAGGGGAAGTTTTGTATATGATATTAAACACTCATAATTTAACAAAATATCTATTCATGCTTTTAACAG

                  *      1620         *      1640         *      1660         *      1680         *      1700
ch12_MH6 : AATTGTTCTTATACAACCTGCAAATGATGTTTATGTCCATATGTTGCAGGTTTATGAAATGGAGACAACCTACCTACAAGAGTCTAATCAATTCGGAAGT : 1691
Unma_KAS : AATTGTTCTTATACAACCTGCAAATGATGTTTATGTCCATATGTTGCAGGTTTATGAAATGGAGACAACCTACCTACAAGAGTCTAATCAATTCGGAAGT : 1691
ch12_NIP : AATTGTTCTTATACAACCTGCAAATGATGTTTATGTCCATATGTTGCAGGTTTATGAAATGGAGACAACCTACCTACAAGAGTCTAATCAATTCGGAAGT : 1676
ch01-ruf : AATTGTTCTTATACAACCTGCAAATGATGTTTATGTCCATATGTTGCAGGTTTATGAAATGGAGACAACCTACCTACAAGAGTCTAATCAATTCGGAAGT : 1692
ch01_MH6 : AATTGTTCTTATACAACCTGCAAATGATGTTTATGTCCATATGTTGCAGGTTTATGAAATGGAGACAACCTACCTACAAGAGTCTAATCAATTCGGAAGT : 1689
ch01_ZS9 : AATTGTTCTTATACAACCTGCAAATGATGTTTATGTCCATATGTTGCAGGTTTATGAAATGGAGACAACCTACCTACAAGAGTCTAATCAATTCGGAAGT : 1689
ch01_IR8 : AATTGTTCTTATACAACCTGCAAATGATGTTTATGTCCATATGTTGCAGGTTTATGAAATGGAGACAACCTACCTACAAGAGTCTAATCAATTCGGAAGT : 1689
ch01_931 : AATTGTTCTTATACAACCTGCAAATGATGTTTATGTCCATATGTTGCAGGTTTATGAAATGGAGACAACCTACCTACAAGAGTCTAATCAATTCGGAAGT : 1689
ch12_ZS9 : AATTGTTCTTATACAACCTGCAAATGATGTTTATGTCCATATGTTGCAGGTTTATGAAATGGAGACAACCTACCTACAAGAGTCTAATCAATTCGGAAGT : 1691
ch01-NIP : AATTGTTCTTATACAACCTGCAAATGATGTTTATGTCCATATGTTGCAGGTTTATGAAATGGAGACAACCTACCTACAAGAGTCTAATCAATTCGGAAGT : 1507
ch01_KAS : AATTGTTCTTATACAACCTGCAAATGATGTTTATGTCCATATGTTGCAGGTTTATGAAATGGAGACAACCTACCTACAAGAGTCTAATCAATTCGGAAGT : 1506
           AATTGTTCTTATACAACCTGCAAATGATGTTTATGTCCATATGTTGCAGGTTTATGAAATGGAGACAACCTACCTACAAGAGTCTAATCAATTCGGAAGT

                  *      1720         *      1740         *      1760         *      1780         *      1800
ch12_MH6 : GTGCTAAAGGGTTTTGAATCATTCTTATCATCGTCAAAGAACACTTCCAAGTAAGAAATCTTTATTTTCTTAGACTGCTACGCCCTTTACTATTTGGAGG : 1791
Unma_KAS : GTGCTAAAGGGTTTTGAATCATTCTTATCATCGTCAAAGAACACTTCCAAGTAAGAAATCTTTATTTTCTTAGACTGCTACGCCCTTTACTATTTGGAGG : 1791
ch12_NIP : GTGCTAAAGGGTTTTGAATCATTCTTATCATCGTCAAAGAACACTTCCAAGTAAGAAATCTTTATTTTCTTAGACTGCTACGCCCTTTACTATTTGGAGG : 1776
ch01-ruf : GTGCTAAAGGGTTTTGAATCATTCTTATCATCGTCAAAGAACACTTCCAAGTAAGAAATCTTTATTTTCTTAGACTGCTACGCCCTTTACTATTTGGAGG : 1792
ch01_MH6 : GTGCTAAAGGGTTTTGAATCATTCTTATCATCGTCAAAGAACACTTCCAAGTAAGAAATCTTTATTTTCTTAGACTGCTACGCCCTTTACTATTTGGAGG : 1789
ch01_ZS9 : GTGCTAAAGGGTTTTGAATCATTCTTATCATCGTCAAAGAACACTTCCAAGTAAGAAATCTTTATTTTCTTAGACTGCTACGCCCTTTACTATTTGGAGG : 1789
ch01_IR8 : GTGCTAAAGGGTTTTGAATCATTCTTATCATCGTCAAAGAACACTTCCAAGTAAGAAATCTTTATTTTCTTAGACTGCTACGCCCTTTACTATTTGGAGG : 1789
ch01_931 : GTGCTAAAGGGTTTTGAATCATTCTTATCATCGTCAAAGAACACTTCCAAGTAAGAAATCTTTATTTTCTTAGACTGCTACGCCCTTTACTATTTGGAGG : 1789
ch12_ZS9 : GTGCTAAAGGGTTTTGAATCATTCTTATCATCGTCAAAGAACACTTCCAAGTAAGAAATCTTTATTTTCTTAGACTGCTACGCCCTTTACTATTTGGAGG : 1791
ch01-NIP : GTGCTAAAGGGTTTTGAATCATTCTTATCATCGTCAAAGAACACTTCCAAGTAAGAAACCTT----TTCTTAGACTGCTATGCCCTTTACTATTTGGAGG : 1603
ch01_KAS : GTGCTAAAGGGTTTTGAATCATTCTTATCATCGTCAAAGAACACTTCCAAGTAAGAAACCTT----TTCTTAGACTGCTATGCCCTTTACTATTTGGAGG : 1602
           GTGCTAAAGGGTTTTGAATCATTCTTATCATCGTCAAAGAACACTTCCAAGTAAGAAAtCTTtattTTCTTAGACTGCTAcGCCCTTTACTATTTGGAGG

                  *      1820         *      1840         *      1860         *      1880         *      1900
ch12_MH6 : TCTTCATTCATTTTCTTTTGACATCCCACTGTATACTTACCAGTTCGAAACCTTCAGAATTCATGCCACATTCTTTGCTTACTTTCAACTGTTGATTGAA : 1891
Unma_KAS : TCTTCATTCATTTTCTTTTGACATCCCACTGTATACTTACCAGTTCGAAACCTTCAGAATTCATGCCACATTCTTTGCTTACTTTCAACTGTTGATTGAA : 1891
ch12_NIP : TCTTCATTCATTTTCTTTTGACATCCCACTGTATACTTACCAGTTCGAAACCTTCAGAATTCATGCCACATTCTTTGCTTACTTTCAACTGTTGATTGAA : 1876
ch01-ruf : TCTTCATTCATTTTCTTTTGACATCCCACTGTATACTTACCAGTTCGAAACCTTCAGAATTCATGCCACATTCTTTGCTTACTTTCAACTGTTGATTGAA : 1892
ch01_MH6 : TCTTCATTCATTTTCTTTTGACATCCCACTGTATACTTACCAGTTCGAAACCTTCAGAATTCATGCCACATTCTTTGCTTACTTTCAACTGTTGATTGAA : 1889
ch01_ZS9 : TCTTCATTCATTTTCTTTTGACATCCCACTGTATACTTACCAGTTCGAAACCTTCAGAATTCATGCCACATTCTTTGCTTACTTTCAACTGTTGATTGAA : 1889
ch01_IR8 : TCTTCATTCATTTTCTTTTGACATCCCACTGTATACTTACCAGTTCGAAACCTTCAGAATTCATGCCACATTCTTTGCTTACTTTCAACTGTTGATTGAA : 1889
ch01_931 : TCTTCATTCATTTTCTTTTGACATCCCACTGTATACTTACCAGTTCGAAACCTTCAGAATTCATGCCACATTCTTTGCTTACTTTCAACTGTTGATTGAA : 1889
ch12_ZS9 : TCTTCATTCATTTTCTTTTGACATCCCACTGTATACTTACCAGTTCGAAACCTTCAGAATTCATGCCACATTCTTTGCTTACTTTCAACTGTTGATTGAA : 1891
ch01-NIP : TCTTCATTCATTTTCTTTTGACATCCCACTGTATACTTACCAGTTCGAAACCTTCAGAATTCATGCCACATTCTTTGCTTACTTCTGACTGTTGATTGAA : 1703
ch01_KAS : TCTTCATTCATTTTCTTTTGACATCCCACTGTATACTTACCAGTTCGAAACCTTCAGAATTCATGCCACATTCTTTGCTTACTTCTGACTGTTGATTGAA : 1702
           TCTTCATTCATTTTCTTTTGACATCCCACTGTATACTTACCAGTTCGAAACCTTCAGAATTCATGCCACATTCTTTGCTTACTTtcaACTGTTGATTGAA

                  *      1920         *      1940         *      1960         *      1980         *      2000
ch12_MH6 : TGACCTACTGTCCTTCCCTTGAACTTTCTACCAGCCTCTGATCACATATAATTTAGCTAATATTCGGATGAATAATTTATAGTTATAGATATTGAACAAA : 1991
Unma_KAS : TGACCTACTGTCCTTCCCTTGAACTTTCTACCAGCCTCTGATCACATATAATTTAGCTAATATTCGGATGAATAATTTATAGTTATAGATATTGAACAAA : 1991
ch12_NIP : TGACCTACTGTCCTTCCCTTGAACTTTCTACCAGCCTCTGATCACATATAATTTAGCTAATATTCGGATGAATAATTTATAGTTATAGATATTGAACAAA : 1976
ch01-ruf : TGACCTACTGTCCTTCCCTTGAACTTTCTACCAGCCTCTGATCACATATAATTTAGCTAATATTCGGATGAATAATTTATAGTTATAGATATTGAACAAA : 1992
ch01_MH6 : TGACCTACTGTCCTTCCCTTGAACTTTCTACCAGCCTCTGATCACATATAATTTAGCTAATATTCGGATGAATAATTTATAGTTATAGATATTGAACAAA : 1989
ch01_ZS9 : TGACCTACTGTCCTTCCCTTGAACTTTCTACCAGCCTCTGATCACATATAATTTAGCTAATATTCGGATGAATAATTTATAGTTATAGATATTGAACAAA : 1989
ch01_IR8 : TGACCTACTGTCCTTCCCTTGAACTTTCTACCAGCCTCTGATCACATATAATTTAGCTAATATTCGGATGAATAATTTATAGTTATAGATATTGAACAAA : 1989
ch01_931 : TGACCTACTGTCCTTCCCTTGAACTTTCTACCAGCCTCTGATCACATATAATTTAGCTAATATTCGGATGAATAATTTATAGTTATAGATATTGAACAAA : 1989
ch12_ZS9 : TGACCTACTGTCCTTCCCTTGAACTTTCTACCAGCCTCTGATCACATATAATTTAGCTAATATTCGGATGAATAATTTATAGTTATAGATATTGAACAAA : 1991
ch01-NIP : TGACCTACTGTCCTGCCCTTGAACTTTCTACCAGCCTCTGATCACATATAATTTAGCTAATATTCAGATGAATAATTTATAGTTATAGATATTGAACAAA : 1803
ch01_KAS : TGACCTACTGTCCTGCCCTTGAACTTTCTACCAGCCTCTGATCACATATAATTTAGCTAATATTCAGATGAATAATTTATAGTTATAGATATTGAACAAA : 1802
           TGACCTACTGTCCTtCCCTTGAACTTTCTACCAGCCTCTGATCACATATAATTTAGCTAATATTCgGATGAATAATTTATAGTTATAGATATTGAACAAA

                  *      2020         *      2040         *      2060         *      2080         *      2100
```

```
ch12_MH6 : AGATCCTCTGATCACATATAATTTACTAATGATCAGATGAATGATGTTGAACAAAAGATCCTCTGATCACATATAATTTACTAATGATCAGATGAACAAA : 2091
Unma_KAS : AGATCCTCTGATCACATATAATTTACTAATGATCAGATGAATGATGTTGAACAAAAGATCCTCTGATCACATATAATTTACTAATGATCAGATGAACAAA : 2091
ch12_NIP : AGATCCTCTGATCACATATAATTTACTAATGATCAGATGAATGATGTTGAACAAAAGATCCTCTGATCACATATAATTTACTAATGATCAGATGAACAAA : 2076
ch01-ruf : AGATCCTCTGATCACATATAATTTACTAATGATCAGATGAATGATGTTGAACAAAAGATCCTCTGATCACATATAATTTACCAATGATCAGATGAACAAA : 2092
ch01_MH6 : AGATCCTCTGATCACATATAATTTACTAATGATCAGATGAATGATGTTGAACAAAAGATCCTCTGATCACATATAATTTACTAATGATCAGATGAACAAA : 2089
ch01_ZS9 : AGATCCTCTGATCACATATAATTTACTAATGATCAGATGAATGATGTTGAACAAAAGATCCTCTGATCACATATAATTTACTAATGATCAGATGAACAAA : 2089
ch01_IR8 : AGATCCTCTGATCACATATAATTTACTAATGATCAGATGAATGATGTTGAACAAAAGATCCTCTGATCACATATAATTTACTAATGATCAGATGAACAAA : 2089
ch01_931 : AGATCCTCTGATCACATATAATTTACTAATGATCAGATGAATGATGTTGAACAAAAGATCCTCTGATCACATATAATTTACTAATGATCAGATGAACAAA : 2089
ch12_ZS9 : AGATCCTCTGATCACATATAATTTACTAATGATCAGATGAATGATGTTGAACAAAAGATCCTCTGATCACATATAATTTACTAATGATCAGATGAACAAA : 2091
ch01-NIP : AAATCCTCTGATCACTTATAATTTA---------------------------------------------------TTTACTAATGATCAGATGAACAAA : 1852
ch01_KAS : AAATCCTCTGATCACTTATAATTTA---------------------------------------------------TTTACTAATGATCAGATGAACAAA : 1851
           AgATCCTCTGATCACaTATAATTTActaatgatcagatgaatgatgttgaacaaaagatcctctgatcacatataaTTTACtAATGATCAGATGAACAAA

                   *      2120        *      2140        *      2160        *      2180        *      2200
ch12_MH6 : AGATCCTCTGATCACTATAGTGGCAGTGGCAGGTATTATACAACAGATTCATAACCCAAAATATGCTATGATAGTGTTATGTCAGTTTATTGGCCAATTG : 2191
Unma_KAS : AGATCCTCTGATCACTATAGTGGCAGTGGCAGGTATTATACAACAGATTCATAACCCAAAATATGCTATGATAGTGTTATGTCAGTTTATTGGCCAATTG : 2191
ch12_NIP : AGATCCTCTGATCACTATAGTGGCAGTGGCAGGTATTATACAACAGATTCATAACCCAAAATATGCTATGATAGTGTTATGTCAGTTTATTGGCCAATTG : 2176
ch01-ruf : AGATCCTCTGATCACTATAGTGGCAGTGGCAGGTATTATACAACAGATTCATAACCCAAAATATGCTATGATAGTGTTATGTCAGTTTATTGGCCAATTG : 2192
ch01_MH6 : AGATCCTCTGATCACTATAGTGGCAGTGGCAGGTATTATACAACAGATTCATAACCCAAAATATGCTATGATAGTGTTATGTCAGTTTATTGGCCAATTG : 2189
ch01_ZS9 : AGATCCTCTGATCACTATAGTGGCAGTGGCAGGTATTATACAACAGATTCATAACCCAAAATATGCTATGATAGTGTTATGTCAGTTTATTGGCCAATTG : 2189
ch01_IR8 : AGATCCTCTGATCACTATAGTGGCAGTGGCAGGTATTATACAACAGATTCATAACCCAAAATATGCTATGATAGTGTTATGTCAGTTTATTGGCCAATTG : 2189
ch01_931 : AGATCCTCTGATCACTATAGTGGCAGTGGCAGGTATTATACAACAGATTCATAACCCAAAATATGCTATGATAGTGTTATGTCAGTTTATTGGCCAATTG : 2189
ch12_ZS9 : AGATCCTCTGATCACTATAGTGGCAGTGGCAGGTATTATACAACAGATTCATAACCCAAAATATGCTATGATAGTGTTATGTCAGTTTATTGGCCAATTG : 2191
ch01-NIP : AGATCCTCTGATCACTCTAGTGGCAGTGGCAGGTATTATACAACAGATTCATAACCCAAAATATGCTATGATAGTGTTATATCAGTTG--TGGCCAATTG : 1950
ch01_KAS : AGATCCTCTGATCACTCTAGTGGCAGTGGCAGGTATTACACAACAGATTCATAACCCAAAATATGCTATGATAGTGTTATATCAGTTG--TGGCCAATTG : 1949
           AGATCCTCTGATCACTaTAGTGGCAGTGGCAGGTATTAtACAACAGATTCATAACCCAAAATATGCTATGATAGTGTTATgTCAGTTtatTGGCCAATTG

                   *      2220        *      2240        *      2260        *      2280        *      2300
ch12_MH6 : CAGGGGTGCTCTATATGCACTATCTAGCATCACCAAGGGAGGGTTGGATTTATGATTACCATTGGCATAATGTTGCGTGTTCATTATTCTGCACAAGCAA : 2291
Unma_KAS : CAGGGGTGCTCTATATGCACTATCTAGCATCACCAAGGGAGGGTTGGATTTATGATTACCATTGGCATAATGTTGCGTGTTCATTATTCTGCACAAGCAA : 2291
ch12_NIP : CAGGGGTGCTCTATATGCACTATCTAGCATCACCAAGGGAGGGTTGGATTTATGATTACCATTGGCATAATGTTGCGTGTTCATTATTCTGCACAAGCAA : 2276
ch01-ruf : CAGGGGTGCTCTATATGCACTATCTAGCATCACCAAGGGAGGGTTGGATTTATGATTACCATTGGCATAATGTTGCGTGTTCATTATTCTGCACAAGCAA : 2292
ch01_MH6 : CAGGGGTGCTCTATATGCACTATCTAGCATCACCAAGGGAGGGTTGGATTTATGATTACCATTGGCATAATGTTGCGTGTTCATTATTCTGCACAAGCAA : 2289
ch01_ZS9 : CAGGGGTGCTCTATATGCACTATCTAGCATCACCAAGGGAGGGTTGGATTTATGATTACCATTGGCATAATGTTGCGTGTTCATTATTCTGCACAAGCAA : 2289
ch01_IR8 : CAGGGGTGCTCTATATGCACTATCTAGCATCACCAAGGGAGGGTTGGATTTATGATTACCATTGGCATAATGTTGCGTGTTCATTATTCTGCACAAGCAA : 2289
ch01_931 : CAGGGGTGCTCTATATGCACTATCTAGCATCACCAAGGGAGGGTTGGATTTATGATTACCATTGGCATAATGTTGCGTGTTCATTATTCTGCACAAGCAA : 2289
ch12_ZS9 : CAGGGGTGCTCTATATGCACTATCTAGCATCACCAAGGGAGGGTTGGATTTATGATTACCATTGGCATAATGTTGCGTGTTCATTATTCTGCACAAGCAA : 2291
ch01-NIP : CAGGGGTGCTCTATATGCACTATCTAGCATCACCAAGGGAGGGTTGGATTTATGATAACCATTGGCATAATCTTCCGTGTTCATTATTCTGCACAAGCAA : 2050
ch01_KAS : CAGGGGTGCTCTATATGCACTATCTAGCATCACCAAGGGAGGGTTGGATTTATGATAACCATTGGCATAATCTTCCGTGTTCATTATTCTGCACAAGCAA : 2049
           CAGGGGTGCTCTATATGCACTATCTAGCATCACCAAGGGAGGGTTGGATTTATGATtACCATTGGCATAATgTTgCGTGTTCATTATTCTGCACAAGCAA

                   *      2320        *      2340        *      2360        *      2380        *      2400
ch12_MH6 : ATGTTTATCATTTCTGTTCTGCTAATAGATCACACAGAATTAGTTATGACAGTTTCTATCTTATTATTTTCAGTCTCAAGCGCTCCAGGAAGTTCCAGG : 2391
Unma_KAS : ATGTTTATCATTTCTGTTCTGCTAATAGATCACACAGAATTAGTTATGACAGTTTCTATCTTATTATTTTCAGTCTCAAGCGCTCCAGGAAGTTCCAGG : 2391
ch12_NIP : ATGTTTATCATTTCTGTTCTGCTAATAGATCACACAGAATTAGTTATGACAGTTTCTATCTTATTATTTTCAGTCTCAAGCGCTCCAGGAAGTTCCAGG : 2376
ch01-ruf : ATGTTTATCATTTCTGTTCTGCTAATAGATCACACAGAATTAGTTATGACAGTTTCTATCTTATTATTTTCAGTCTCAAGCGCTCCAGGAAGTTCCAGG : 2392
ch01_MH6 : ATGTTTATCATTTCTGTTCTGCTAATAGATCACACAGAATTAGTTATGACAGTTTCTATCTTATTATTTTCAGTCTCAAGCGCTCCAGGAAGTTCCAGG : 2389
ch01_ZS9 : ATGTTTATCATTTCTGTTCTGCTAATAGATCACACAGAATTAGTTATGACAGTTTCTATCTTATTATTTTCAGTCTCAAGCGCTCCAGGAAGTTCCAGG : 2389
ch01_IR8 : ATGTTTATCATTTCTGTTCTGCTAATAGATCACACAGAATTAGTTATGACAGTTTCTATCTTATTATTTTCAGTCTCAAGCGCTCCAGGAAGTTCCAGG : 2389
ch01_931 : ATGTTTATCATTTCTGTTCTGCTAATAGATCACACAGAATTAGTTATGACAGTTTCTATCTTATTATTTTCAGTCTCAAGCGCTCCAGGAAGTTCCAGG : 2389
ch12_ZS9 : ATGTTTATCATTTCTGTTCTGCTAATAGATCACACAGAATTAGTTATGACAGTTTCTATCTTATTATTTTCAGTCTCAAGCGCTCCAGGAAGTTCCAGG : 2391
ch01-NIP : ATGTTTATCATGCCTGTTCTGCTAATAGGTCACATAGAATTAGTTATGACAGTTTCTGTCTTATTAATTTCAGTCTCAAGCGCTCCAGGAAGTTCCAGG : 2150
ch01_KAS : ATGTTTATCATGCCTGTTCTGCTAATAGGTCACATAGAATTAGTTATGACAGTTTCTGTCTTATTAATTTCAGTCTCAAGCGCTCCAGGAAGTTCCAGG : 2149
           ATGTTTATCATttCTGTTCTGCTAATAGaTCACAcAGAATTAGTTATGACAGTTTCTaTCTTATTAtTTTCAGTCTCAAGCGCTCCAGGAAGTTCCAGG

                   *      2420        *      2440        *      2460        *      2480        *      2500
ch12_MH6 : CTGATGAGAGGCTGTTCTCCCTGTCATCGGTTACATCTCCAGCGGTATGACTAATGATCCTAAATCCTAATGC-ACTGAAAAGCCCGTGCCC-CTTTTTT : 2489
Unma_KAS : CTGATGAGAGGCTGTTCTCCCTGTCATCGGTTACATCTCCAGCGGTATGACTAATGATCCTAAATCCTAATGC-ACTGAAAAGCCCGTGCCC-CTTTTTT : 2489
ch12_NIP : CTGATGAGAGGCTGTTCTCCCTGTCATCGGTTACATCTCCAGCGGTATGACTAATGATCCTAAATCCTAATGC-ACTGAAAAGCCCGTGCCC-CTTTTTT : 2474
ch01-ruf : CTGATGAGAGGCTGTTCTCCCTGTCATCGGTTACATCTCCAGCGGTATGACTAATGATCCTAAATCCTAATGC-ACTGAAAAGCCCGTGCCC-CTTTTTT : 2490
ch01_MH6 : CTGATGAGAGGCTGTTCTCCCTGTCATCGGTTACATCTCCAGCGGTATGACTAATGATCCTAAATCCTAATGC-ACTGAAAAGCCCGTGCCC-CTTTTTT : 2487
ch01_ZS9 : CTGATGAGAGGCTGTTCTCCCTGTCATCGGTTACATCTCCAGCGGTATGACTAATGATCCTAAATCCTAATGC-ACTGAAAAGCCCGTGCCC-CTTTTTT : 2487
ch01_IR8 : CTGATGAGAGGCTGTTCTCCCTGTCATCGGTTACATCTCCAGCGGTATGACTAATGATCCTAAATCCTAATGC-ACTGAAAAGCCCGTGCCC-CTTTTTT : 2487
ch01_931 : CTGATGAGAGGCTGTTCTCCCTGTCATCGGTTACATCTCCAGCGGTATGACTAATGATCCTAAATCCTAATGC-ACTGAAAAGCCCGTGCCC-CTTTTTT : 2487
ch12_ZS9 : CTGATGAGAGGCTGTTCTCCCTGTCATCGGTTACATCTCCAGCGGTATGACTAATGATCCTAAATCCTAATGC-ACTGAAAAGCCCGTGCCC-CTTTTTT : 2489
ch01-NIP : CTGATGAGAGGCTGTTCTCCCTGTCATCGGTTACATCTCCAGCGGTATGACTAATGATCCTAAATCCTAATGCCACTGAAAAGCCAGTGCCCGTTTTTTT : 2250
ch01_KAS : CTGATGAGAGGCTGTTCTCCCTGTCATCGGTTACATCTCCAGCGGTATGACTAATGATCCTAAATCCTAATGCCACTGAAAAGCCAGTGCCCGTTTTTTT : 2249
           CTGATGAGAGGCTGTTCTCCCTGTCATCGGTTACATCTCCAGCGGTATGACTAATGATCCTAAATCCTAATGC ACTGAAAAGCCcGTGCCC cTTTTTT

                   *      2520        *      2540        *      2560        *      2580        *      2600
ch12_MH6 : AATCTTTTGGTTTGCTTTGATCGATGATTGTTATTCCCCCTGTGCTATACTGATATTTGTGAGTTTGCTAATACATACCATTATTTTCTATTCATTCCAG : 2589
Unma_KAS : AATCTTTTGGTTTGCTTTGATCGATGATTGTTATTCCCCCTGTGCTATACTGATATTTGTGAGTTTGCTAATACATACCATTATTTTCTATTCATTCCAG : 2589
ch12_NIP : AATCTTTTGGTTTGCTTTGATTGATGATTGTTATTCCCCCTGTGCTATACTGATATTTGTGAGTTTGCTAATACATACCATTATTTTCTATTCATTCCAG : 2574
ch01-ruf : AATCTTTTGGTTTGCTTTGATCGATGATTGTTATTCCCCCTGTGCTATACTGATATTTGTGAGTTTGCTAATACATACCATTATTTTCTATTCATTCCAG : 2590
ch01_MH6 : AATCTTTTGGTTTGCTTTGATCGATGATTGTTATTCCCCCTGTGCTATACTGATATTTGTGAGTTTGCTAATACATACCATTATTTTCTATTCATTCCAG : 2587
ch01_ZS9 : AATCTTTTGGTTTGCTTTGATCGATGATTGTTATTCCCCCTGTGCTATACTGATATTTGTGAGTTTGCTAATACATACCATTATTTTCTATTCATTCCAG : 2587
ch01_IR8 : AATCTTTTGGTTTGCTTTGATCGATGATTGTTATTCCCCCTGTGCTATACTGATATTTGTGAGTTTGCTAATACATACCATTATTTTCTATTCATTCCAG : 2587
ch01_931 : AATCTTTTGGTTTGCTTTGATCGATGATTGTTATTCCCCCTGTGCTATACTGATATTTGTGAGTTTGCTAATACATACCATTATTTTCTATTCATTCCAG : 2587
ch12_ZS9 : AATCTTTTGGTTTGCTTTGATCGATGATTGTTATTCCCCCTGTGCTATACTGATATTTGTGAGTTTGCTAATACATACCATTATTTTCTATTCATTCCAG : 2589
ch01-NIP : AATCTGTTGGTTTGCTTTGATCGATGATTGTTATTCCCCCTGTGCTATACCGATATTTGTGAGGTTGCTAATACATACCATTATTTTTTATTCATTCCAG : 2350
ch01_KAS : AATCTGTTGGTTTGCTTTGATCGATGATTGTTATTCCCCCTGTGCTATACCGATATTTGTGAGGTTGCTAATACATACCATTATTTTTTATTCATTCCAG : 2349
           AATCTtTTGGTTTGCTTTGATcGATGATTGTTATTCCCCCTGTGCTATACtGATATTTGTGAGtTTGCTAATACATACCATTATTTTcTATTCATTCCAG

                   *      2620        *      2640        *      2660        *      2680        *      2700
ch12_MH6 : GTCGATGAACATATGGCTGGACGAGATGGTAAGTTCTCTTTCTTGTTTTCATTAAGCTGAAACCAGGAACAATTGAGACTCTGGGCTCAATACTAGCTGC : 2689
Unma_KAS : GTCGATGAACATATGGCTGGACGAGATGGTAAGTTCTCTTTCTTGTTTTCATTAAGCTGAAACCAGGAACAATTGAGACTCTGGGCTCAATACTAGCTGC : 2689
ch12_NIP : GTCGATGAACATATGGCTGGACGAGATGGTAAGTTCTCTTTCTTGTTTTCATTAAGCTGAAACCAGGAACAATTGAGACTCTGGGCTCAATACTAGCTGC : 2674
ch01-ruf : GTCGATGAACATATGGCTGGACGAGATGGTAAGTTCTCTTTCTTGTTTTCATTAAGCTGAAACCAGGAACAATTGAGACTCTGGGCTCAATACTAGCTGC : 2690
ch01_MH6 : GTCGATGAACATATGGCTGGACGAGATGGTAAGTTCTCTTTCTTGTTTTCATTAAGCTGAAACCAGGAACAATTGAGACTCTGGGCTCAATACTAGCTGC : 2687
ch01_ZS9 : GTCGATGAACATATGGCTGGACGAGATGGTAAGTTCTCTTTCTTGTTTTCATTAAGCTGAAACCAGGAACAATTGAGACTCTGGGCTCAATACTAGCTGC : 2687
ch01_IR8 : GTCGATGAACATATGGCTGGACGAGATGGTAAGTTCTCTTTCTTGTTTTCATTAAGCTGAAACCAGGAACAATTGAGACTCTGGGCTCAATACTAGCTGC : 2687
ch01_931 : GTCGATGAACATATGGCTGGACGAGATGGTAAGTTCTCTTTCTTGTTTTCATTAAGCTGAAACCAGGAACAATTGAGACTCTGGGCTCAATACTAGCTGC : 2687
ch12_ZS9 : GTCGATGAACATATGGCTGGACGAGATGGTAAGTTCTCTTTCTTGTTTTCATTAAGCTGAAACCAGGAACAATTGAGACTCTGGGCTCAATACTAGCTGC : 2689
ch01-NIP : GTCGATGAACATATGGCTGGACGAGATGGTAAGTTCTCTTTCTTGTTTTCATTAAGCTGAAACCAGGAACAATTGAGACTCTGGGCTCAATACCAGCTGC : 2450
```

```
ch01_KAS  : GTCGATGAACATATAGCTGGACGAGATGGTAAGTTCTCTTTCTTGTTTTCATTAAGCTGAAACCAGGAACAATTGAGACTCTGGGCTCAATACCAGCTGC : 2449
            GTCGATGAACATATgGCTGGACGAGATGGTAAGTTCTCTTTCTTGTTTTCATTAAGCTGAAACCAGGAACAATTGAGACTCTGGGCTCAATACtAGCTGC

                   *      2720         *      2740         *      2760         *      2780         *      2800
ch12_MH6  : ATCCTGGCAGATGGAAGAGAATATGGATCAGGTCGTTCGAAAGGTGCAACCACTCCTGCAAACGGGCAGTAAGTGCTCAGATTACCTTTGTGGCAAAAAA : 2789
Unma_KAS  : ATCCTGGCAGATGGAAGAGAATATGGATCAGGTCGTTCGAAAGGTGCAACCACTCCTGCAAACGGGCAGTAAGTGCTCAGATTACCTTTGTGGCAAAAAA : 2789
ch12_NIP  : ATCCTGGCAGATGGAAGAGAATATGGATCAGGTCGTTCGAAAGGTGCAACCACTCCTGCAAACGGGCAGTAAGTGCTCAGATTACCTTTGTGGCAAAAAA : 2774
ch01-ruf  : ATCCTGGCAGATGGAAGAGAATATGGATCAGGTCGTTCGAAAGGTGCAACCACTCCTGCAAACGGGCAGTAAGTGCTCAGATTACCTTTGTGGCAAAAAA : 2790
ch01_MH6  : ATCCTGGCAGATGGAAGAGAATATGGATCAGGTCGTTCGAAAGGTGCAACCACTCCTGCAAACGGGCAGTAAGTGCTCAGATTACCTTTGTGGCAAAAAA : 2787
ch01_ZS9  : ATCCTGGCAGATGGAAGAGAATATGGATCAGGTCGTTCGAAAGGTGCAACCACTCCTGCAAACGGGCAGTAAGTGCTCAGATTACCTTTGTGGCAAAAAA : 2787
ch01_IR8  : ATCCTGGCAGATGGAAGAGAATATGGATCAGGTCGTTCGAAAGGTGCAACCACTCCTGCAAACGGGCAGTAAGTGCTCAGATTACCTTTGTGGCAAAAAA : 2787
ch01_931  : ATCCTGGCAGATGGAAGAGAATATGGATCAGGTCGTTCGAAAGGTGCAACCACTCCTGCAAACGGGCAGTAAGTGCTCAGATTACCTTTGTGGCAAAAAA : 2787
ch12_ZS9  : ATCCTGGCAGATGGAAGAGAATATGGATCAGGTCGTTCGAAAGGTGCAACCACTCCTGCAAACGGGCAGTAAGTGCTCAGATTACCTTTGTGGCAAAAAA : 2789
ch01-NIP  : ATCCTGGCAGATGGAAGAGAATATGGATCAGGTCGTTCGAAAGGCGCAACCACTCCTGCAAACGGGCAGTAAGTGCTCAGATTACTTTTGCGGCAAAAAA : 2550
ch01_KAS  : ATCCTGGCAGATGGAAGAGAATATGGATCAGGTCGTTCGAAAGGCGCAACCACTCCTGCAAACGGGCAGTAAGTGCTCAGATTACTTTTGCGGCAAAAAA : 2549
            ATCCTGGCAGATGGAAGAGAATATGGATCAGGTCGTTCGAAAGGtGCAACCACTCCTGCAAACGGGCAGTAAGTGCTCAGATTACcTTTGtGGCAAAAAA

                   *      2820         *      2840         *      2860         *      2880         *      2900
ch12_MH6  : TGTGCTATCCTTATCAGCTTTACATACATCCTTGCAAGGGGCAGTGTTTGTCTAACACTTCTTTATATTAAAAAAAAAACAGTGTTTCTAGGAACAACCCT : 2889
Unma_KAS  : TGTGCTATCCTTATCAGCTTTACATACATCCTTGCAAGGGGCAGTGTTTGTCTAACACTTCTTTATATTAAAAAAAA-CAGTGTTTCTAGGAACAACCCT : 2888
ch12_NIP  : TGTGCTATCCTTATCAGCTTTACATACATCCTTGCAAGGGGCAGTGTTTGTCTAACACTTCTTTATATTAAAAAAAA-CAGTGTTTCTAGGAACAACCCT : 2873
ch01-ruf  : TGTGCTATCCTTATCAGCTTTACATACATCCTTGCAAGGGGCAGTGTTTGTCTAACACTTCTTTATATTAAAAAAAA-CAGTGTTTCTAGGAACAACCCT : 2889
ch01_MH6  : TGTGCTATCCTTATCAGCTTTACATACATCCTTGCAAGGGGCAGTGTTTGTCTAACACTTCTTTATATTAAAAAAAA-CAGTGTTTCTAGGAACAACCCT : 2886
ch01_ZS9  : TGTGCTATCCTTATCAGCTTTACATACATCCTTGCAAGGGGCAGTGTTTGTCTAACACTTCTTTATATTAAAAAAAA-CAGTGTTTCTAGGAACAACCCT : 2886
ch01_IR8  : TGTGCTATCCTTATCAGCTTTACATACATCCTTGCAAGGGGCAGTGTTTGTCTAACACTTCTTTATATTAAAAAAAA-CAGTGTTTCTAGGAACAACCCT : 2886
ch01_931  : TGTGCTATCCTTATCAGCTTTACATACATCCTTGCAAGGGGCAGTGTTTGTCTAACACTTCTTTATATTAAAAAAAA-CAGTGTTTCTAGGAACAACCCT : 2886
ch12_ZS9  : TGTGCTATCCTTATCAGCTTTACATACATCCTTGCAAGGGGCAGTGTTTGTCTAACACTTCTTTATATTAAAAAAAA-CAGTGTTTCTAGGAACAACCCT : 2888
ch01-NIP  : TGTGCTATCGTTCTCAACTTTACATACATTCTTGCCAGGGGCAGTGTTTGTCTAACACTTCTTTATATTAAAAATAC-CAGTGTTTCTAGGAACAACCCT : 2649
ch01_KAS  : TGTGCTATCGTTCTCAACTTTACATACATTCTTGCCAGGGGCAGTGTTTGTCTAACACTTCTTTATATTAAAAATAC-CAGTGTTTCTAGGAACAACCCT : 2648
            TGTGCTATCcTTaTCAgCTTTACATACATCcTTGCaAGGGGCAGTGTTTGTCTAACACTTCTTTATATTAAAAAaAa CAGTGTTTCTAGGAACAACCCT

                   *      2920         *      2940         *      2960         *      2980         *      3000
ch12_MH6  : TTTAGCCCTTGTTTAGTTCCAAACTTTTTCTTCAAACTTCCAACTTTTCCATCACATCAAAACTTTCCTACACGCACAAACTTCCAACTTTTCCGTCACA : 2989
Unma_KAS  : TTTAGCCCTTGTTTAGTTCCAAACTTTTTCTTCAAACTTCCAACTTTTCCATCACATCAAAACTTTCCTACACGCACAAACTTCCAACTTTTCCGTCACA : 2988
ch12_NIP  : TTTAGCCCTTGTTTAGTTCCAAACTTTTTCTTCAAACTTCCAACTTTTCCATCACATCAAAACTTTCCTACACGCACAAACTTCCAACTTTTCCGTCACA : 2973
ch01-ruf  : TTTAGCCCTTGTTTAGTTCCAAACTTTTTCTTCAAACTTCCAACTTTTCCATCACATCAAAACTTTCCTACACGCACAAACTTCCAACTTTTCCGTCACA : 2989
ch01_MH6  : TTTAGCCCTTGTTTAGTTCCAAACTTTTTCTTCAAACTTCCAACTTTTCCATCACATCAAAACTTTCCTACACGCACAAACTTCCAACTTTTCCGTCACA : 2986
ch01_ZS9  : TTTAGCCCTTGTTTAGTTCCAAACTTTTTCTTCAAACTTCCAACTTTTCCATCACATCAAAACTTTCCTACACGCACAAACTTCCAACTTTTCCGTCACA : 2986
ch01_IR8  : TTTAGCCCTTGTTTAGTTCCAAACTTTTTCTTCAAACTTCCAACTTTTCCATCACATCAAAACTTTCCTACACGCACAAACTTCCAACTTTTCCGTCACA : 2986
ch01_931  : TTTAGCCCTTGTTTAGTTCCAAACTTTTTCTTCAAACTTCCAACTTTTCCATCACATCAAAACTTTCCTACACGCACAAACTTCCAACTTTTCCGTCACA : 2986
ch12_ZS9  : TTTAGCCCTTGTTTAGTTCCAAACTTTTTCTTCAAACTTCCAACTTTTCCATCACATCAAAACTTTCCTACACGCACAAACTTCCAACTTTTCCGTCACA : 2988
ch01-NIP  : TTT------------------------------------------------------------------------------------------------- : 2652
ch01_KAS  : TTT------------------------------------------------------------------------------------------------- : 2651
            TTTagcccttgtttagttccaaactttttcttcaaacttccaacttttccatcacatcaaaactttcctacacgcacaaacttccaacttttccgtcaca

                   *      3020         *      3040         *      3060         *      3080         *      3100
ch12_MH6  : TTGTTCCAATTTCAACCAAACTTCCAATTTTAGCGTGAACTAAACACACCCTTAGTAGAATTGCCTAGTTCTGACCCCTCGTGTGTTTGGACAGAGGAAA : 3089
Unma_KAS  : TTGTTCCAATTTCAACCAAACTTCCAATTTTAGCGTGAACTAAACACACCCTTAGTAGAATTGCCTAGTTCTGACCCCTCGTGTGTTTGGACAGAGGAAA : 3088
ch12_NIP  : TTGTTCCAATTTCAACCAAACTTCCAATTTTAGCGTGAACTAAACACACCCTTAGTAGAATTGCCTAGTTCTGACCCCTCGTGTGTTTGGACAGAGGAAA : 3073
ch01-ruf  : TTGTTCCAATTTCAACCAAACTTCCAATTTTAGCGTGAACTAAACACACCCTTAGTAGAATTGCCTAGTTCTGACCCCTCGTGTGTTTGGACAGAGGAAA : 3089
ch01_MH6  : TTGTTCCAATTTCAACCAAACTTCCAATTTTAGCGTGAACTAAACACACCCTTGGTAGAATTGCCTAGTTCTGACCCCTCGTGTGTTTGGACAGAGGAAA : 3086
ch01_ZS9  : TTGTTCCAATTTCAACCAAACTTCCAATTTTAGCGTGAACTAAACACACCCTTGGTAGAATTGCCTAGTTCTGACCCCTCGTGTGTTTGGACAGAGGAAA : 3086
ch01_IR8  : TTGTTCCAATTTCAACCAAACTTCCAATTTTAGCGTGAACTAAACACACCCTTGGTAGAATTGCCTAGTTCTGACCCCTCGTGTGTTTGGACAGAGGAAA : 3086
ch01_931  : TTGTTCCAATTTCAACCAAACTTCCAATTTTAGCGTGAACTAAACACACCCTTGGTAGAATTGCCTAGTTCTGACCCCTCGTGTGTTTGGACAGAGGAAA : 3086
ch12_ZS9  : TTGTTCCAATTTCAACCAAACTTCCAATTTTAGCGTGAACTAAACACACCCTTAGTAGAATTGCCTAGTTCTGACCCCTCGTGTGTTTGGACAGAGGAAA : 3088
ch01-NIP  : -----------------------------------------------------AGTTGAATTGCCTAGTTCTGACCCCTCGTGTGTTTGGACAGAGGAAA : 2699
ch01_KAS  : -----------------------------------------------------AGTTGAATTGCCTAGTTCTGACCTCTCGTGTGTTTGGACAGAGGAAA : 2698
            ttgttccaatttcaaccaaacttccaattttagcgtgaactaaacacacccctt GTaGAATTGCCTAGTTCTGACCcCTCGTGTGTTTGGACAGAGGAAA

                   *      3120         *      3140         *      3160         *      3180         *      3200
ch12_MH6  : ACCAAAGAAGGGAGGTCGCCCTGGAGGGAGAGACGGCAAGAGAATACGGCCATCAAATGATCCAGATCTGGACGACGAAGAGGACTTCTAACTCGACATG : 3189
Unma_KAS  : ACCAAAGAAGGGAGGTCGCCCTGGAGGGAGAGACGGCAAGAGAATACGGCCATCAAATGATCCAGATCTGGACGACGAAGAGGACTTCTAACTCGACATG : 3188
ch12_NIP  : ACCAAAGAAGGGAGGTCGCCCTGGAGGGAGAGACGGCAAGAGAATACGGCCATCAAATGATCCAGATCTGGACGACGAAGAGGACTTCTAACTCGACATG : 3173
ch01-ruf  : ACCAAAGAAGGGAGGTCGCCCTGGAGGGAGAGACGGCAAGAGAATACGGCCATCAAATGATCCAGATCTGGACGACGAAGAGGACTTCTAACTCGACATG : 3189
ch01_MH6  : ACCAAAGAAGGGAGGTCGCCCTGGAGGGAGAGACGGCAAGAGAATACGGCCATCAAATGATCCAGATCTGGACGACGAAGAGGACTTCTAACTCGACATG : 3186
ch01_ZS9  : ACCAAAGAAGGGAGGTCGCCCTGGAGGGAGAGACGGCAAGAGAATACGGCCATCAAATGATCCAGATCTGGACGACGAAGAGGACTTCTAACTCGACATG : 3186
ch01_IR8  : ACCAAAGAAGGGAGGTCGCCCTGGAGGGAGAGACGGCAAGAGAATACGGCCATCAAATGATCCAGATCTGGACGACGAAGAGGACTTCTAACTCGACATG : 3186
ch01_931  : ACCAAAGAAGGGAGGTCGCCCTGGAGGGAGAGACGGCAAGAGAATACGGCCATCAAATGATCCAGATCTGGACGACGAAGAGGACTTCTAACTCGACATG : 3186
ch12_ZS9  : ACCAAAGAAGGGAGGTCGCCCTGGAGGGAGAGACGGCAAGAGAATACGGCCATCAAATGATCCAGATCTGGACGACGAAGAGGACTTCTAACTCGACATG : 3188
ch01-NIP  : ACCAAAGAAAGGAGGTCGCCCTGGAGGGAGAGACGGCAAGAGAATACGGCCATCAAATGATCCAGATCTGGACGATGAAGAGGACTTCTAACTCGACATG : 2799
ch01_KAS  : ACCAAAGAAAGGAGGTCGCCCTGGAGGGAGAGACGGCAAGAGAATACGGCCATCAAATGATCCAGATCTGGACGATGAAGAGGACTTCTAACTCGACATG : 2798
            ACCAAAGAAgGGAGGTCGCCCTGGAGGGAGAGACGGCAAGAGAATACGGCCATCAAATGATCCAGATCTGGACGAcGAAGAGGACTTCTAACTCGACATG

                   *      3220         *      3240         *      3260         *      3280         *      3300
ch12_MH6  : TTGTAGGCCTAGCTCAGTGATGATTTAACCTTGCTAGACTCATGCTTCGAATCATTCTTTCTGTATTGTAATTTTTTTT-AATCTTGTGTTGCAATCCAT : 3288
Unma_KAS  : TTGTAGGCCTAGCTCAGTGATGATTTAACCTTGCTAGACTCATGCTTCGAATCATTCTTTCTGTATTGTAATTTTTTTTTAATCTTGTGTTGCAATCCAT : 3288
ch12_NIP  : TTGTAGGCCTAGCTCAGTGATGATTTAACCTTGCTAGACTCATGCTTCGAATCATTCTTTCTGTATTGTAATTTTTTTTTAATCTTGTGTTGCAATCCAT : 3273
ch01-ruf  : TTGTAGGCCTAGCTCAGTGATGATTTAACCTTGCTAGACTCATGCTTCGAATCATTCTTTCTGTATTGTAATTTTTTTTTAATCTTGTGTTGCAATCCAT : 3289
ch01_MH6  : TTGTAGGCCTAGCTCAGTGATGATTTAACCTTGCTAGACTCATGCTTCGAATCATTCTTTCTGTATTGTAATTTTTTTTTAATCTTGTGTTGCAATCCAT : 3286
ch01_ZS9  : TTGTAGGCCTAGCTCAGTGATGATTTAACCTTGCTAGACTCATGCTTCGAATCATTCTTTCTGTATTGTAATTTTTTTTTAATCTTGTGTTGCAATCCAT : 3286
ch01_IR8  : TTGTAGGCCTAGCTCAGTGATGATTTAACCTTGCTAGACTCATGCTTCGAATCATTCTTTCTGTATTGTAATTTTTTTTTAATCTTGTGTTGCAATCCAT : 3286
ch01_931  : TTGTAGGCCTAGCTCAGTGATGATTTAACCTTGCTAGACTCATGCTTCGAATCATTCTTTCTGTATTGTAATTTTTTTTTAATCTTGTGTTGCAATCCAT : 3286
ch12_ZS9  : TTGTAGGCCTAGCTCAGTGATGATTTAACCTTGCTAGACTCATGCTTCGAATCATTCTTTCTGTATTGTAATTTTTTTTTAATCTTGTGTTGCAATCCAT : 3288
ch01-NIP  : TTGTAGGCCTAGCTCAGTGATGATTTAACCTTGCTAGACTCATGCTTCGAGTCATTCTTTCTGTATTGTAATTTTTTTT-AATCTTGTGTTGCAATCCAT : 2898
ch01_KAS  : TTGTAGGCCTAGCTCAGTGATGATTTAACCTTGCTAGACTCATGCTTCGAATCATTCTTTCTGTATTGTAATTTTTTTT-AATCTTGTGTTGCAATCCAT : 2897
            TTGTAGGCCTAGCTCAGTGATGATTTAACCTTGCTAGACTCATGCTTCGAaTCATTCTTTCTGTATTGTAATTTTTTTT AATCTTGTGTTGCAATCCAT

                   *      3320         *      3340         *      3360         *      3380         *      3400
ch12_MH6  : GGCAAGCAAAGCCTTTTCCCCAGGGAGTAGCCTTGCTCATCTAGTCATCTTATCGTCGGCATTTGCCTGTGCTT-GCATTTTCTTCCTTTTCAACCCACC : 3387
Unma_KAS  : GGCAAGCAAAGCCTTTTCCCCAGGGAGTAGCCTTGCTCATCTAGTCATCTTATCGTCGGCATTTGCCTGTGCTT-GCATTTTCTTCCTTTTCAACCCACC : 3387
ch12_NIP  : GGCAAGCAAAGCCTTTTCCCCAGGGAGTAGCCTTGCTCATCTAGTCATCTTATCGTCGGCATTTGCCTGTGCTT-GCATTTTCTTCCTTTTCAACCCACC : 3372
ch01-ruf  : GGCAAGCAAAGCCTTTTCCCCAGGGAGTAGCCTTGCTCATCTAGTCATCTTATCGTCGGCATTTGCCTGTGCTT-GCATTTTCTTCCTTTTCAACCCACC : 3388
ch01_MH6  : GGCAAGCAAAGCCTTTTCCCCAGGGAGTAGCCTTGCTCATCTAGTCATCTTATCGTCGGCATTTGCCTGTGCTT-GTATTTTCTTCCTTTTCAACCCACC : 3385
ch01_ZS9  : GGCAAGCAAAGCCTTTTCCCCAGGGAGTAGCCTTGCTCATCTAGTCATCTTATCGTCGGCATTTGCCTGTGCTT-GTATTTTCTTCCTTTTCAACCCACC : 3385
```

```
ch01_IR8  : GGCAAGCAAAGCCTTTTCCCCAGGGAGTAGCCTTGCTCATCTAGTCATCTTATCGTCGGCATTTGCCTGTGCTT-GTATTTTCTTCCTTTTCAACCCACC : 3385
ch01_931  : GGCAAGCAAAGCCTTTTCCCCAGGGAGTAGCCTTGCTCATCTAGTCATCTTATCGTCGGCATTTGCCTGTGCTT-GTATTTTCTTCCTTTTCAACCCACC : 3385
ch12_ZS9  : GGCAAGCAAAGCCTTTTCCCCAGGGAGTAGCCTTGCTCATCTAGTCATCTTATCGTCGGCATTTGCCTGTGCTT-GCATTTTCTTCCTTTTCAACCCACC : 3387
ch01-NIP  : GGCAAGCAAAGCCTTTTCCCCAGGGAGTAGCCTTGCTCATCTAGT-ATCTTATCATCGGCATTTGCCTGTGCTT-GCATTTTCTTCCTTTTCAACCCACC : 2996
ch01_KAS  : GGCAAGCAAAGCCTTTTCCCCAGGGAGTAGCCTTGCTCATCTAGT-ATATTATCATCGGCATTTGCCTGTGCTTTGCATTTTCTTCCTTTTCAACCCACC : 2996
            GGCAAGCAAAGCCTTTTCCCCAGGGAGTAGCCTTGCTCATCTAGTcATcTTATCgTCGGCATTTGCCTGTGCTT G ATTTTCTTCCTTTTCAACCCACC

                  *     3420         *      3440         *      3460         *      3480         *      3500
ch12_MH6  : CCGGCGTTGTCTGATTGTCTCCCGTTTTGTAAAGCAGTAGCAAGTTAATTA---------------------------------GACGGTGATAATTTCT : 3454
Unma_KAS  : CCGGCGTTGTCTGATTGTCTCCCGTTTTGTAAAGCAGTAGCAAGTTAATTA---------------------------------GACGGTGATAATTTCT : 3454
ch12_NIP  : CCGGCGTTGTCTGATTGTCTCCCGTTTTGTAAAGCAGTAGCAAGTTAATTA---------------------------------GACGGTGATAATTTCT : 3439
ch01-ruf  : CCGGCGTTGTCTGATTGTCTCCCGTTTTGTAAAGCAGTAGCAAGTTAATTA---------------------------------GACGGTGATAATTTCT : 3455
ch01_MH6  : CCGGCGTTGTCTGATTGGCTCCCGTTTTGTAAAGCAGTAGCAAGTTAATTA---------------------------------GACGGTGATAATTTCT : 3452
ch01_ZS9  : CCGGCGTTGTCTGATTGGCTCCCGTTTTGTAAAGCAGTAGCAAGTTAATTA---------------------------------GACGGTGATAATTTCT : 3452
ch01_IR8  : CCGGCGTTGTCTGATTGGCTCCCGTTTTGTAAAGCAGTAGCAAGTTAATTA---------------------------------GACGGTGATAATTTCT : 3452
ch01_931  : CCGGCGTTGTCTGATTGGCTCCCGTTTTGTAAAGCAGTAGCAAGTTAATTA---------------------------------GACGGTGATAATTTCT : 3452
ch12_ZS9  : CCGGCGTTGTCTGATTGTCTCCCGTTTTGTAAAGCAGTAGCAAGTTAATTA---------------------------------GACGGTGATAATTTCT : 3454
ch01-NIP  : TCGGCGTTGTCTGATTGTCTCCCGTTTTGTAAAGCAGTAGCAGGTTAATTA---------------------------------GACGGTGATAATTTCT : 3063
ch01_KAS  : TCGGCGTTGTCTGATTGTCTCCCGTTTTGTAAAGCAGTAGCAGGTTAATTATCTCCGTTTTGTAAAGCAGTAGCAGGTTAATTAGACGGTGATAATTTCT : 3096
            cCGGCGTTGTCTGATTG CTCCCGTTTTGTAAAGCAGTAGCAaGTTAATTA                                 GACGGTGATAATTTCT

                  *     3520         *      3540         *      3560         *      3580         *      3600
ch12_MH6  : GTTAACTCTACTGTAAACAACGTTGCTATAGATTCCAGACTCCAGTGAGACGAGGATGAGACTCCAGCGAGACGAGAATCTGGATTGGCCGATTCTTAGT : 3554
Unma_KAS  : GTTAACTCTACTGTAAACAACGTTGCTATAGATTCCAGACTCCAGTGAGACGAGGATGAGACTCCAGCGAGACGAGAATCTGGATTGGCCGATTCTTAGT : 3554
ch12_NIP  : GTTAACTCTACTGTAAACAACGTTGCTATAGATTCCAGACTCCAGTGAGACGAGGATGAGACTCCAGCGAGACGAGAATCTGGATTGGCCGATTCTTAGT : 3539
ch01-ruf  : GTTAACTCTACTGTAAACAACGTTGCTATAGATTCCAGACTCCAGTGAGACGAGGATGAGACTCCAGCGAGACGAGAATCTGGATTGGCCGATTCTTAGT : 3555
ch01_MH6  : GTTAACTCTACTGTAAACAACGTTGCTATAGATTCCAGACTCCAGTGAGACGAGGATGAGACTCCAGCGAGACGAGAATCTGGATTGGCCGATTCTTAGT : 3552
ch01_ZS9  : GTTAACTCTACTGTAAACAACGTTGCTATAGATTCCAGACTCCAGTGAGACGAGGATGAGACTCCAGCGAGACGAGAATCTGGATTGGCCGATTCTTAGT : 3552
ch01_IR8  : GTTAACTCTACTGTAAACAACGTTGCTATAGATTCCAGACTCCAGTGAGACGAGGATGAGACTCCAGCGAGACGAGAATCTGGATTGGCCGATTCTTAGT : 3552
ch01_931  : GTTAACTCTACTGTAAACAACGTTGCTATAGATTCCAGACTCCAGTGAGACGAGGATGAGACTCCAGCGAGACGAGAATCTGGATTGGCCGATTCTTAGT : 3552
ch12_ZS9  : GTTAACTCTACTGTAAACAACGTTGCTATAGATTCCAGACTCCAGTGAGACGAGGATGAGACTCCAGCGAGACGAGAATCTGGATTGGCCGATTCTTAGT : 3554
ch01-NIP  : GTTAACTCTACTGTAAACAACGTTGCTATAGATTCCAGACTCCAGTGAGACGAGGATGAGACTCCAGCGAGACGAGAATCTGGATTGGCCAATTCTTAGT : 3163
ch01_KAS  : GTTAACTCTACTGTAAACAACGTTGCTATAGATTCCAGACTCCAGTGAGACGAGGATGAGACTCCAGCGAGACGAGAATCTGGATTGGCCGATTCTTAGT : 3196
            GTTAACTCTACTGTAAACAACGTTGCTATAGATTCCAGACTCCAGTGAGACGAGGATGAGACTCCAGCGAGACGAGAATCTGGATTGGCCgATTCTTAGT

                  *     3620         *      3640         *      3660         *      3680         *      3700
ch12_MH6  : ACCAGAAAAAAAATGTCTCATCCGATTATAGGTTTTTATATTTTGGAACGTGTAGTTGCTAGCACACCG--------CCGCGTTTGCCAGGGCCAAGGTGG : 3646
Unma_KAS  : ACCAGAAAAAAAATGTCTCATCCGATTATAGGTTTTTATATTTTGGAACGTGTAGTTGCTAGCACACCG--------CCGCGTTTGCCAGGGCCAAGGTGG : 3646
ch12_NIP  : ACCAGAAAAAAAATGTCTCATCCGATTATAGGTTTTTATATTTTGGAACGTGTAGTTGCTAGCACACCG--------CCGCGTTTGCCAGGGCCAAGGTGG : 3631
ch01-ruf  : ACCAGAAAAAAAATGTCTCATCCGATTATAGGTTTTTATATTTTGGAACGTGTAGTTGCTAGCACACCG--------CCGCGTTTGCCAGGGCCAAGGTGG : 3647
ch01_MH6  : ACCAGAAAAAAAATGTCTCATCCGATTATAGGTTTTTATATTTTGGAACGTGTAGTTGCTAGCACACCG--------CCGCGTTTGCCAGGGCCAAGGTG- : 3643
ch01_ZS9  : ACCAGAAAAAAAATGTCTCATCCGATTATAGGTTTTTATATTTTGGAACGTGTAGTTGCTAGCACACCG--------CCGCGTTTGCCAGGGCCAAGGTG- : 3643
ch01_IR8  : ACCAGAAAAAAAATGTCTCATCCGATTATAGGTTTTTATATTTTGGAACGTGTAGTTGCTAGCACACCG--------CCGCGTTTGCCAGGGCCAAGGTG- : 3643
ch01_931  : ACCAGAAAAAAAATGTCTCATCCGATTATAGGTTTTTATATTTTGGAACGTGTAGTTGCTAGCACACCG--------CCGCGTTTGCCAGGGCCAAGGTG- : 3643
ch12_ZS9  : ACCAGAAAAAAAATGTCTCATCCGATTATAGGTTTTTATATTTTGGAACGTGTAGTTGCTAGCACACCG--------CCGCGTTTGCCAGGGCCAAGGTGG : 3646
ch01-NIP  : ACTAGAAAAAAAATGTCTCATCTGATTATAGGTTTTTATATTTTGGGACGTGTAGTTGCTAGCACACCGGCACACCGCCACGTTTGCAAGGGCCAAGGTGG : 3263
ch01_KAS  : ACTAGAAAAAAAATGTCTCATCTGATTATAGGCTTTTATATTTTGGGACGTGTAGTTGCTAGCACACCGGCACACCGCCACGTTTGCAAGGGCCAAGGTGG : 3296
            ACcAGAAAAAAAATGTCTCATCcGATTATAGGtTTTTATATTTTGGaACGTGTAGTTGCTAGCACACCG        CCgCGTTTGCcAGGGCCAAGGTG

                  *     3720         *      3740         *      3760         *      3780         *      3800
ch12_MH6  : AGTTGAATCTGTGAGTTCACCTGTTGCATTGCAAGACAAGCTGGAACTTGTAATCTCAACAATTAGATTGCAGGGTGCCTGAGGTTAATTTGTGTACACT : 3746
Unma_KAS  : AGTTGAATCTGTGAGTTCACCTGTTGCATTGCAAGACAAGCTGGAACTTGTAATCTCAACAATTAGATTGCAGGGTGCCTGAGGTTAATTTGTGTACACT : 3746
ch12_NIP  : AGTTGAATCTGTGAGTTCACCTGTTGCATTGCAAGACAAGCTGGAACTTGTAATCTCAACAATTAGATTGCAGGGTGCCTGAGGTTAATTTGTGTACACT : 3731
ch01-ruf  : AGTTGAATCTGTGAGTTCACCTGTTGCATTGCAAGACAAGCTGGAACTTGTAATCTCAACAATTAGATTGCAGGGTGCCTGAGGTTAATTTGTGTACACT : 3747
ch01_MH6  : ------------GAGTTCACCTGTTGCATTGCAAGACAAGCTGGAACTTGTAATCTCAACAATTAGATTGCAGGGTGCCTGAGGTTAATTTGTGTACACT : 3731
ch01_ZS9  : ------------GAGTTCACCTGTTGCATTGCAAGACAAGCTGGAACTTGTAATCTCAACAATTAGATTGCAGGGTGCCTGAGGTTAATTTGTGTACACT : 3731
ch01_IR8  : ------------GAGTTCACCTGTTGCATTGCAAGACAAGCTGGAACTTGTAATCTCAACAATTAGATTGCAGGGTGCCTGAGGTTAATTTGTGTACACT : 3731
ch01_931  : ------------GAGTTCACCTGTTGCATTGCAAGACAAGCTGGAACTTGTAATCTCAACAATTAGATTGCAGGGTGCCTGAGGTTAATTTGTGTACACT : 3731
ch12_ZS9  : AGTTGAATCTGTGAGTTCACCTGTTGCATTGCAAGACAAGCTGGAACTTGTAATCTCAACAATTAGATTGCAGGGTGCCTGAGGTTAATTTGTGTACACT : 3746
ch01-NIP  : AGTTGAATCTGTGAGTTCACCTGTTGCATTGCAAGACAAGCTGGAACTTGTAATCTCAACCATTAGATTGCAGGGTGCCTGAGGTTAATTTGTGTACACT : 3363
ch01_KAS  : AGTTGAATCTGTGAGTTCACCTGTTGCATTGCAAGACAAGCTGGAACTTGTAATCTCAACCATTAGATTGCAGGGTGCCTGAGGTTAATTTGTGTACACT : 3396
                        GAGTTCACCTGTTGCATTGCAAGACAAGCTGGAACTTGTAATCTCAACaATTAGATTGCAGGGTGCCTGAGGTTAATTTGTGTACACT

                  *     3820         *      3840         *      3860         *      3880         *      3900
ch12_MH6  : TTTTTTTAGTGTAGCTAGCAAAGACTACAAGTAATGCTTTTTATATTAGTATAGGTGATTAATTAGCTCTAAGTACAATATGATGATGCAACACGCAGGC : 3846
Unma_KAS  : TTTTTTTAGTGTAGCTAGCAAAGACTACAAGTAATGCTTTTTATATTAGTATAGGTGATTAATTAGCTCTAAGTACAATATGATGATGCAACACGCAGGC : 3846
ch12_NIP  : TTTTTTTAGTGTAGCTAGCAAAGACTACAAGTAATGCTTTTTATATTAGTATAGGTGATTAATTAGCTCTAAGTACAATATGATGATGCAACACGCAGGC : 3831
ch01-ruf  : TTTTTTTAGTGTAGCTAGCAAAGACTACAAGTAATGCTTTTTATATTAGTATAGGTGATTAATTAGCTCTAAGTACAATATGATGATGCAACACGCAGGC : 3847
ch01_MH6  : TTTTTTTAGTGTAGCTAGCAAAGACTACAAGTAATGCTTTTTATATTAGTATAGGTGATTAATTAGCTCTAAGTACAATATGATGATGCAACACGCAGGC : 3831
ch01_ZS9  : TTTTTTTAGTGTAGCTAGCAAAGACTACAAGTAATGCTTTTTATATTAGTATAGGTGATTAATTAGCTCTAAGTACAATATGATGATGCAACACGCAGGC : 3831
ch01_IR8  : TTTTTTTAGTGTAGCTAGCAAAGACTACAAGTAATGCTTTTTATATTAGTATAGGTGATTAATTAGCTCTAAGTACAATATGATGATGCAACACGCAGGC : 3831
ch01_931  : TTTTTTTAGTGTAGCTAGCAAAGACTACAAGTAATGCTTTTTATATTAGTATAGGTGATTAATTAGCTCTAAGTACAATATGATGATGCAACACGCAGGC : 3831
ch12_ZS9  : TTTTTTTAGTGTAGCTAGCAAAGACTACAAGTAATGCTTTTTATATTAGTATAGGTGATTAATTAGCTCTAAGTACAATATGATGATGCAACACGCAGGC : 3846
ch01-NIP  : TTTTTT-AGTGTAGCTAGCAAAGACTACAAGTAATGCTTTTTATATTAGTATAGGTGATTAGT-----CTAAGTACAATATGATGGTGCAACACGCAGGC : 3457
ch01_KAS  : TTTTTT-AGTGTAGCTAGCAAAGACTACAAGTAATGCTTTTTATATTAGTATAGGTGATTAATTAGCTCTAAGTACAATATGATGGTGCAACACGCAGGC : 3495
           TTTTTTtAGTGTAGCTAGCAAAGACTACAAGTAATGCTTTTTATATTAGTATAGGTGATTAaTtagctCTAAGTACAATATGATGaTGCAACACGCAGGC

                  *     3920         *      3940         *      3960         *      3980         *      4000
ch12_MH6  : ATGCATGTCTAAACAGCTGTGCTTAGCTTCAAGTATGGAAATAGCTTGAGCATCACTTCTTCAACAGGTTGCATTGACAAATAATCCCTCCGTCCCAAAA : 3946
Unma_KAS  : ATGCATGTCTAAACAGCTGTGCTTAGCTTCAAGTATGGAAATAGCTTGAGCATCACTTCTTCAACAGGTTGCATTGACAAATAATCCCTCCGTCCCAAAA : 3946
ch12_NIP  : ATGCATGTCTAAACAGCTGTGCTTAGCTTCAAGTATGGAAATAGCTTGAGCATCACTTCTTCAACAGGTTGCATTGACAAATACTCCCTCCGTCCCAAAA : 3931
ch01-ruf  : ATGCATGTCTAAACAGCTGTGCTTAGCTTCAAGTATGGAAATAGCTTGAGCATCACTTCTTCAACAGGTTGCATTGACAAATACTCCCTCCGTCCCAAAA : 3947
ch01_MH6  : ATGCATGTCTAAACAGCTGTGCTTAGCTTCAAGCATGGAAATAGCTTGAGCATCACTTCTTCAACAGGTTGCATTGACAAATACTCCCTCCGTCCCGAAA : 3931
ch01_ZS9  : ATGCATGTCTAAACAGCTGTGCTTAGCTTCAAGCATGGAAATAGCTTGAGCATCACTTCTTCAACAGGTTGCATTGACAAATACTCCCTCCGTCCCGAAA : 3931
ch01_IR8  : ATGCATGTCTAAACAGCTGTGCTTAGCTTCAAGCATGGAAATAGCTTGAGCATCACTTCTTCAACAGGTTGCATTGACAAATACTCCCTCCGTCCCGAAA : 3931
ch01_931  : ATGCATGTCTAAACAGCTGTGCTTAGCTTCAAGCATGGAAATAGCTTGAGCATCACTTCTTCAACAGGTTGCATTGACAAATACTCCCTCCGTCCCGAAA : 3931
ch12_ZS9  : ATGCATGTCTAAACAGCTGTGCTTAGCTTCAAGTATGGAAATAGCTTGAGCATCACTTCTTCAACAGGTTGCATTGACAAATACTCCCTCCGTCCCAAAA : 3946
ch01-NIP  : ATGCATGTCTAAACAGCTGTGCTTAGCTTCAAGTATGGAAATAGCTTGAGCATCACTTCTTCAACAGGTTGCATTGACAAATACTCCCTCCGTCCCAAAA : 3557
ch01_KAS  : ATGCATGTCTAAACAGCTGTGCTTAGCTTCAAGTATGGAAATAGCTTGAGCATCACTTCTTCAACAGGTTGCATTGACAAATACTCCCTCCGTCCCAAAA : 3595
            ATGCATGTCTAAACAGCTGTGCTTAGCTTCAAG ATGGAAATAGCTTGAGCATCACTTCTTCAACAGGTTGCATTGACAAATAcTCCCTCCGTCCC AAA

                  *     4020         *      4040         *      4060         *      4080         *      4100
ch12_MH6  : TAAGTGCAGTTTTGCACTATTTATCTTCAATGTTTGATTATGTTTGATTGTTCATCTTATTT-AAAAAAAAATTATGATTACTATTTTTATTGTTATTAG : 4045
Unma_KAS  : TAAGTGCAGTTTTGCACTATTTATCTTCAATGTTTGATTATGTTTGATTGTTCATCTTATTT-AAAAAAAA-TTATGATTACTATTTTTATTGTTATTAG : 4044
```

```
ch12_NIP  : TAAGTGCAGTTTTGCACTATTTATCTTCAATGTTTGATTATGTTTGATTTTTCGTCTTATTT-AAAAAAAA-TTATGATTACTATTTTTATTGTTATTAG : 4029
ch01-ruf  : TAAGTGCAGTTTTGCACTATTTATCTTCAATGTTTGATTATGTTTGATTGTTCGTCTTATTT-AAAAAAAAATTATGATTACTATTTTTATTGTTATTAG : 4046
ch01_MH6  : TAAGTGCAGTTTTGCACTATTTATCTTCAATGTTTGATTATGTTTGATTGTTCGTCTTATTT-AAAAAAAAATTATGATTACTATTTTTATTGTTATTAG : 4030
ch01_ZS9  : TAAGTGCAGTTTTGCACTATTTATCTTCAATGTTTGATTATGTTTGATTGTTCGTCTTATTT-AAAAAAAAATTATGATTACTATTTTTATTGTTATTAG : 4030
ch01_IR8  : TAAGTGCAGTTTTGCACTATTTATCTTCAATGTTTGATTATGTTTGATTGTTCGTCTTATTT-AAAAAAAAATTATGATTACTATTTTTATTGTTATTAG : 4030
ch01_931  : TAAGTGCAGTTTTGCACTATTTATCTTCAATGTTTGATTATGTTTGATTGTTCGTCTTATTT-AAAAAAAAATTATGATTACTATTTTTATTGTTATTAG : 4030
ch12_ZS9  : TAAGTGCAGTTTTGCACTATTTATCTTCAATGTTTGATTATGTTTGATTGTTCGTCTTATTT-AAAAAAAAATTATGATTACTATTTTTATTGTTATTAG : 4045
ch01-NIP  : TAAGTGCAGTTTTGCACTATTTATCTTTAATTTTTGATT----------GTTCGTCTTATTTGAAAAAAAATTTATGATTACTATTTTTATTGTTATTAG : 3647
ch01_KAS  : TAAGTGCAGTTTTGCACTATTTATCTTCCATGTTTGATT----------GTTTGTCTTATTTGAAAAAAAAATTATGATTACTATTTTTATTGTTATTAG : 3685
            TAAGTGCAGTTTTGCACTATTTATCTTcaATgTTTGATTatgtttgattgTTcgTCTTATTT AAAAAAAA TTATGATTACTATTTTTATTGTTATTAG

                  *     4120         *     4140         *     4160         *     4180         *     4200
ch12_MH6  : ATGATAAAACATGAATAGTACTTTATGTGTGACTACTTTTACTTTAGGATGGACGTAGTATTTTTCATTTTCAGAATCTTAAATGCATTTGTC-CA--TG : 4142
Unma_KAS  : ATGATAAAACATGAATAGTACTTTATGTGTGACTACTTTTACTTTAGGATGGACGTAGTATTTTTCATTTTCAGAATCTTAAATGCATTTGTC-CA--TG : 4141
ch12_NIP  : ATGATAAAACATGAATAGTACTTTATGTGTGACTACTTTTAAAAAATAAT--TCATAAATTTTTCAAATAAAACGGACGGTCAAACATTGGACACT--TT : 4125
ch01-ruf  : ATGATAAAACATGAATAGTACTTTATGTGTGACTACTTTTAAAAAATAAT--TCATAAATTTTTCAAATAAAACGGACGGTCAAACATTGGACACT--TT : 4142
ch01_MH6  : ATGATAAAACATGAATAGTACTTTACGTGTGACTACTTTTAAAAAATAAT--TCATAAATTTTTCAAATAAAACGGACGGTCAAACATTGGACACT--TT : 4126
ch01_ZS9  : ATGATAAAACATGAATAGTACTTTACGTGTGACTACTTTTAAAAAATAAT--TCATAAATTTTTCAAATAAAACGGACGGTCAAACATTGGACACT--TT : 4126
ch01_IR8  : ATGATAAAACATGAATAGTACTTTACGTGTGACTACTTTTAAAAAATAAT--TCATAAATTTTTCAAATAAAACGGACGGTCAAACATTGGACACT--TT : 4126
ch01_931  : ATGATAAAACATGAATAGTACTTTACGTGTGACTACTTTTAAAAAATAAT--TCATAAATTTTTCAAATAAAACGGACGGTCAAACATTGGACACT--TT : 4126
ch12_ZS9  : ATGATAAAACATGAATAGTACTTTATGTGTGACTACTTTTAAAAAATAAT--TCATAAATTTTTCAAATAAAACGGACGGTCAAACATTGGACACT--TT : 4141
ch01-NIP  : ATGATAAAACATAAATAGTACTTTATGTGTGACTATTTTTAAAAAATAAT--TCATAAATTTTTCAAATAAGACGGACGGTCAAGCATTGGACACTGGTT : 3745
ch01_KAS  : ATGATAAAACATGAATAGTACTTTATATGTGACTATTTTTAAAAAATAAT--TCATAAATTTTTCAAATAAGACGGACGGTCAAGTATTGGACACTGGTT : 3783
            ATGATAAAACATgAATAGTACTTTA gTGTGACTAcTTTTAaaaaAtaAT  tCaTAaatTTTTcaAaTaa AcggaCggtcAa cATTgGaCaCt  Tt

                  *     4220         *     4240
ch12_MH6  : AAAACATCTTTCGTGGAAAAGATGT-CAGAAGCTTTTAT---- : 4180
Unma_KAS  : AAAACATCTTTCGTGGAAAAGATGT-CAGAAGCTTTTAT---- : 4179
ch12_NIP  : AGGATGGACGTAGTATTTTTCATTTTCAGAATCTTAAAT---- : 4164
ch01-ruf  : AGGATGGACGTAGTATTTTTCATTTTCAGAATCTTAAAT---- : 4181
ch01_MH6  : AGGATGGACGTAGTATTTTTCATTTTCAGAATCTTAAAT---- : 4165
ch01_ZS9  : AGGATGGACGTAGTATTTTTCATTTTCAGAATCTTAAAT---- : 4165
ch01_IR8  : AGGATGGACGTAGTATTTTTCATTTTCAGAATCTTAAAT---- : 4165
ch01_931  : AGGATGGACGTAGTATTTTTCATTTTCAGAATCTTAAAT---- : 4165
ch12_ZS9  : AGGATGGACGTAGTATTTTTCATTTTCAGAATCTTAAA----- : 4179
ch01-NIP  : TCCACGACTGCACTTATTTTGGGATGGAGGTAGTATTTTTCAT : 3788
ch01_KAS  : TCCACG------------------------------------- : 3789
            a  A g    t gt       at t cagaa ctt  at
```

Supplementary Figure 7. Alignment of genome sequences of *EAF6* gene. Franking sequences (-500 bp upstream and +1000 bp downstream sequences) are included in order to show the positions of SNPs used for allele distribution analysis (Figure 4A). The SNPs are highlighted in magenta, and start and stop codons are highlighted in green. NIP: Nipponbare (*japonica*), 931: 93-11(*indica*), IR8: IR8 (*indica*), MH6: Minghui 63 (*indica*), ZH9: Zhenshan 97 (*indica*), KAS: Kasalath (*indica*), ruf: *O. rufipogon* W1943. ch1: chromosome 1, ch12: chromosome 12, Unma: Unmapped sequence.

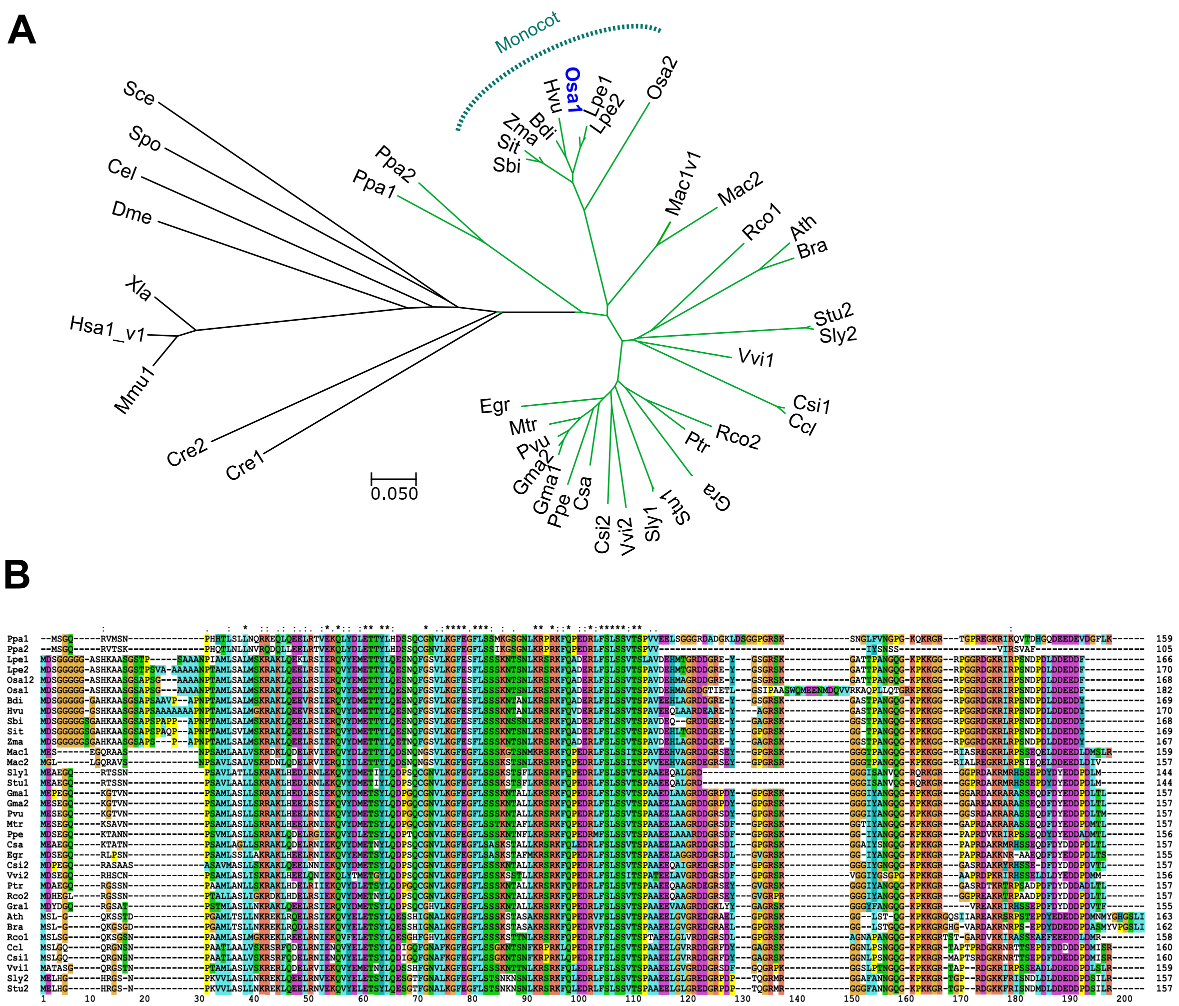

Supplementary Figure 8. Phylogenetic tree analysis and multiple alignment of EAF6 protein in plants and other eukaryotic organisms. (**A**) Un-rooted neighbor-joining tree for the total protein sequences of EAF6 protein from 33 organisms. Branch of the plant species is shown with a green line. Details of the protein homologs and species names are shown in Table S2. (**B**) Multiple sequence alignment of EAF6 proteins from 25 species. Similar amino acids are shown with the same color box.

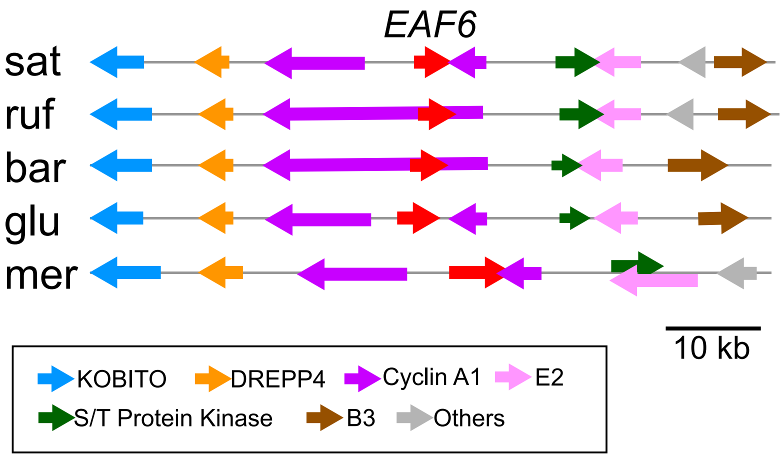

Supplementary Figure 9. Conserved gene block on chromosome 1 among *Oryza* AA genome species. Gene annotation of the region surrounding the *EAF6* locus (red arrow) was shown. sat: *Oryza sativa* ssp. *japonica*, ruf: *O. rufipogon*, bar: *O.barthii*, glu: *O. glumaepatula*, mer: *O. meridionalis*.

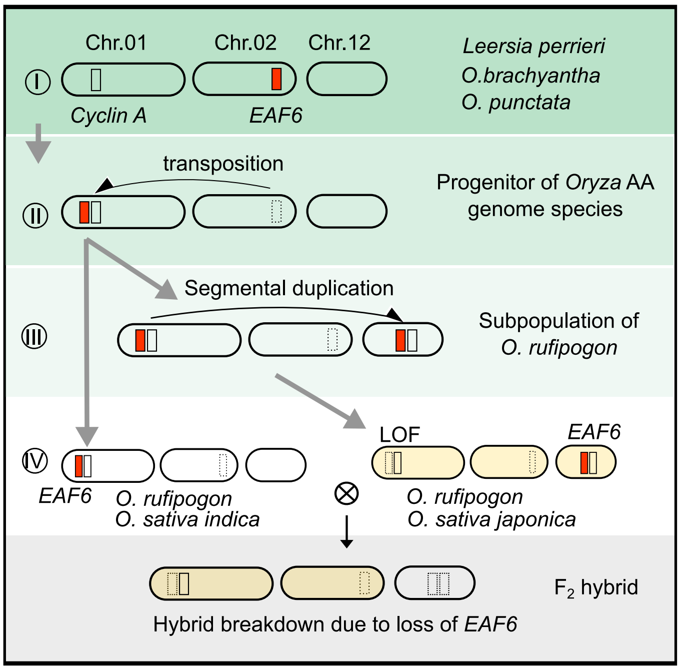

Supplementary Figure 10. Hypothetical model for evolution process of *Oryza EAF6*. Step I: *EAF6* is located on chromosome 2 of the common ancestor of *Oryza* and *Leersia*. II: Transposition in the progenitor of the *Oryza* AA genome species. *EAF6–Cyclin-A1* linkage block arose in this step. III: Segmental duplication occurred, and the new copied segment jumped to chromosome 12 (in ancestral *O. rufpipogon* species). IV: Loss of function mutation (LOF) of the *EAF6* gene on chromosome 12 in a subpopulation of *O. rufipogon*.

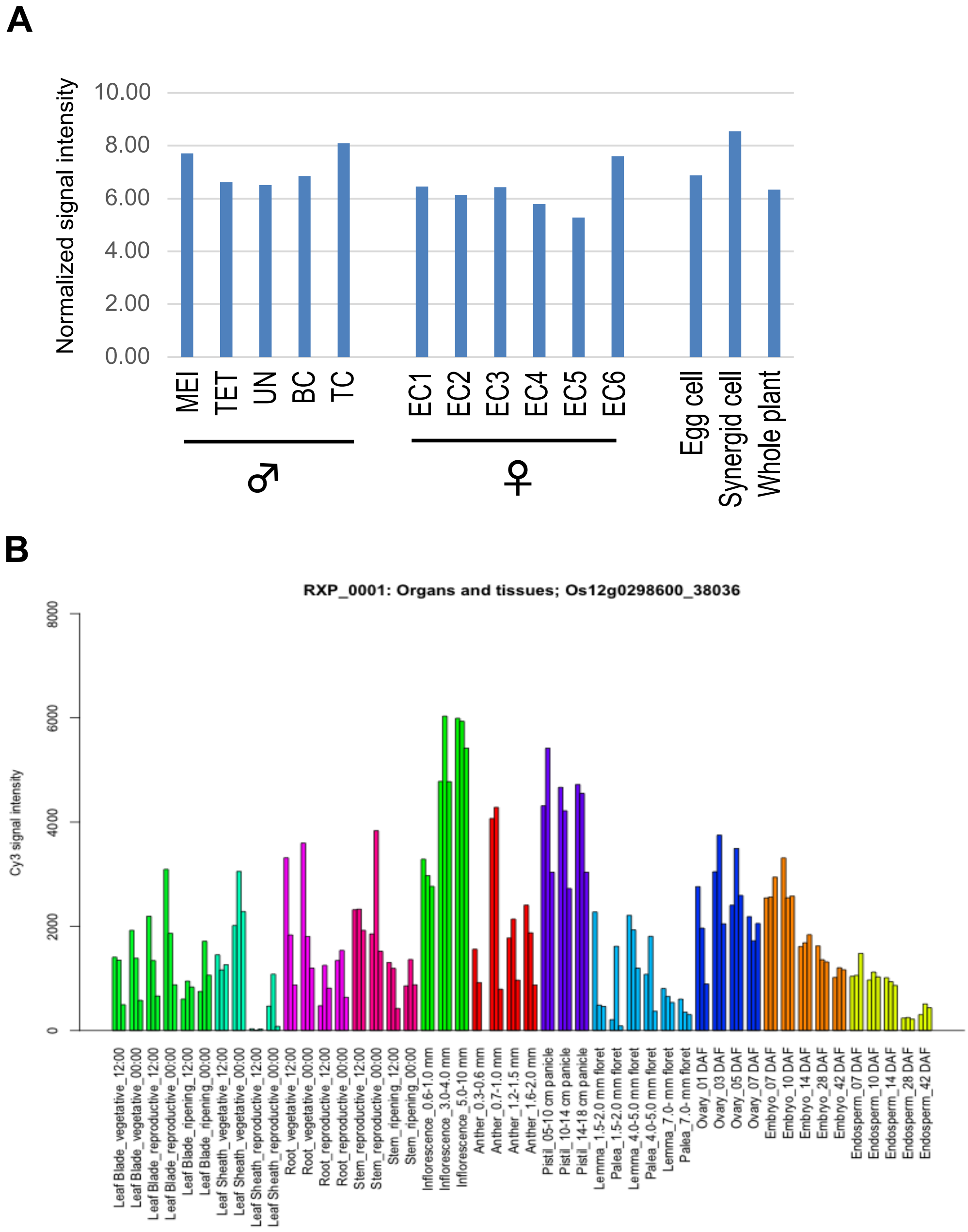

Supplementary Figure 11. Expression pattern of *OsEAF6* (*Os12g0298600*) based on the public microarray database (Gene Expression Omnibus). (**A**) Expression in male and female gametophytes prepared, ♂: GSE29217 (Hobo et al. 2008), and ♀: GSE35760 (Kubo et al. 2013). Egg cell and synergid cells: GSE21074 (Onishi et al. 2011). (**B**) Data from RiceXpro database.

Supplementary Table 1. List of primers used in this study.

Primer	Forward	Reverse	Chr.	Position (bp) #
**PCR marker for genotyping**				
12c106	CCTTTGACGGGGTGAAAGTA	GGCCGTAATAGACGAGACCA	12	10,615,896
12c107	GTTTGCAGCGGAACTCACTA	TAGCATCTGGCTGCTCACTG	12	11,429,960
12c102	AACTCGTCCTCCTCCCTCTC	TCTGGTGTGAGCACTGATCG	12	14,210,148
12c101	TAAGTTCCTGCTCCCTCTGC	TGGTTGGATGGCTAAAATCTG	12	15,825,298
1c203	TGTGATTGTGAAAGGGGACA	ACTGCCTCACTGGCTCACTC	1	7,175,814
1c208	TGAAACGGAGGGAGTAGCAG	CCACCGAAGCAGAAGCTAGA	1	7,315,500
1c210	CCGTGATGATTTGCACTTGA	GCGTATGCCGCTTCACTTAT	1	7,352,250
1c215	CACTTGGCATGCAAGAACAT	CATGTTTAATCACCCCCAAAA	1	7,363,863
1c213	GCGTCTCCCAGGTTGATGTA	GATGAGAAGCAATTCGTCCAA	1	7,370,122
1c217	CGAGGGTTTGGGTGCTCTT	AGCACATGCCACTGCTTGAA	1	7,393,665
1c218	TTGGTGTGGAGCAAAGTGAG	GGAGATGCAAACGAGCTACC	1	7,398,425
1c219*	GCTTGCACATTGACATGGAT	CCATGCTTCAGAAAATTCCA	1	7,402,593
1c206	ACGAAGGCTTGTCCAATACG	TCGGATTTGTTCTGTTGCTG	1	7,408,970
1c216	TTGATTCCTCTTTCCCTTGC	ATACCTTCACACTATCATCTTTG	1	7,414,418
1c212	CATGGACAGCCTCCAAGAAG	GAGGCTGTTGCTGAGGTGTT	1	7,429,989
1c214	TGACCATTCGTCTTATTCAAAA	GTAGTGGTTGTATCGTACTC	1	7,437,623
HPT	CGCAAGGAATCGGTCAATAC	CGTCTGCTGCTCCATACAAG	-	-
**RT-PCR**				
eaf6sp	ACCCTAGAAACCCTGACCA	AGTTAGAAGTCCTCTTCGTC	12	11,862,836
eaf6co	ATTCGGAAGTGTGCTAAAGG	TCGAAGCATGAGTCTAGCAA	12	11,864,020
Act1	AACTGGGATGATATGGAGAA	CCTCCAATCCAGACACTGTA	3	29,074,326
**Gene cloning**				
G20310	TGGACTCCCCTCTCACACTT	CTGTTCGTGTTGCTCTTGTG	12	11,859,982
G20310pro	ggtaccTGGACTCCCCTCTCACACTT	actagtGGTCAGGGTTTCTAGGGTTCG	12	11,859,982
G20324	CGGACTTCGGACCTGTTTTA	CATTGCAAGACAAGCTGGAA	12	11,860,529
we1fulSpF	CGCactagtGACTCGGGAGGCGGAGGA	CGCactagtGAGTTAGAAGTCCTCTTCGTCGT	12	11,862,857
mCheNde-f	cccccatATGGTGAGCAAGGGCGAGGAG	catatgAatcgataaATTTAAATCCactagtGTGAGCA AGGGCGAGGAG	-	-

* 1c219 is a CAPS marker which needs *ScaI* digestion after PCR amplification

#Position of the forward primer is shown based on Nipponbare genome (IRGSP1.0)

Supplementary Table 2. List of EAF6 homologs used in phylogenetic analysis.

Symbol	Taxon	Chr. No.	Locus ID	Protein	Protein length
**Monocot**					
Osa1	*Oryza sativa*	12	LOC_Os12g20310	ABA97832	168
Osa2	*Oryza sativa*	1	LOC_Os01g13250		182
Bdi	*Brachypodium distachyon*	3	LOC100823473	XP_003570195	169
Lpe1	*Leersia perrieri*	5	LPERR05G21690	A0A0D9WJU5	166
Lpe2	*Leersia perrieri*	2	LPERR02G24000	A0A0D9VK07	170
Sbi	*Sorghum bicolor*	4	Sb04g030500	XP_002454419.1	168
Sit	*Setaria italica*	I	LOC101756945	XP_004953620.1	169
Zma	*Zea mays*	4	LOC100274188	XP_008679014.1	167
Hvu	*Hordeum vulgare*	6H	AK361108.1	BAJ92315.1	170
**Dicot**					
Ath	*Arabidopsis thaliana*	4	AT4G14385.1	NP_567429.1	163
Bra	*Brassica rapa*	A4	LOC103863523	XP_009139514.1	162
Ccl	*Citrus clementina*	-	Ciclev10006103m	XP_006420226.1	160
Csa	*Cucumis sativus*	7	LOC101220539	XP_011659255.1	157
Csi1	*Citrus sinensis*	9	LOC102608676	XP_006489735.1	160
Csi2	*Citrus sinensis*	5	LOC102617199	XP_006479546.1	157
Egr	*Eucalyptus grandis*	11	Eucgr.K03263.1	XP_010037979.1	155
Gma1	*Glycine max*	13	LOC100499798	NP_001236194.1	157
Gma2	*Glycine max*	10	LOC100306348	NP_001237402.1	157
Gra	*Gossypium raimondii*	4	LOC105792308	XP_012476272.1	155
Mtr	*Medicago truncatula*	1	Medtr1g069925.1	XP_013468491.1	157
Ppe	*Prunus persica*	-	PRUPE_ppa012749mg	XP_007200424.1	156
Ptr	*Populus trichocarpa*	LGII	POPTR_0002s03980g	XP_002302046	157
Pvu	*Phaseolus vulgaris*	7	PHAVU_007G193100g	XP_007144901.1	157
Rco1	*Ricinus communis*	-	RCOM_1006710	XP_002531526.1	158
Rco2	*Ricinus communis*	-	RCOM_1214110	XP_002524619.1	157
Sly1	*Solanum lycopersicum*	9	LOC101252026	XP_004247520.1	158
Sly2	*Solanum lycopersicum*	9	LOC101252527	XP_004247732.1	157
Stu1	*Solanum tuberosum*	-	LOC102599499	XP_006351676.1	144
Stu2	*Solanum tuberosum*	-	LOC102588268	XP_006354485.1	157
Vvi1	*Vitis vinifera*	5	LOC100259638	XP_002285184.1	159
Vvi2	*Vitis vinifera*	7	LOC100260000	XP_010652367.1	156
**Other plant species**					
Mac1	*Musa acuminata*	6	LOC103987354	XP_009403912.1	159
Mac2	*Musa acuminata*	10	LOC103969831	XP_009381747.1	157
Ppa1	*Physcomitrella patens*	-	PHYPADRAFT_199311	XP_001783399.1	159
Ppa2	*Physcomitrella patens*	-	PHYPADRAFT_141449	XP_001774534	105
**Other species**					
Cre1	*Chlamydomonas reinhardtii*	-	CHLREDRAFT_144397	XP_001690646	96
Cre2	*Chlamydomonas reinhardtii*	-	CHLREDRAFT_184521	XP_001695403	119
Dme	*Drosophila melanogaster*	3	NT_037436.4	AAF50760.1	225
Xla	*Xenopus laevis*	-	BC094399	NP_001090025.1	188
Cel	*Caenorhabditis elegans*	I	B0025.4	CCD61198.1	148
Mmu1	*Mus musculus*	4	NM_027310	NP_081586.1	192
Hsa1_v1	*Homo sapiens*	1	NM_022756	NP_073593.2	201
Spo	*Schizosaccharomyces pombe*	I	N.A	NP_593902.1	138
Sce	*Saccharomyces cerevisiae*	X	N.A	P47128.1	113

Chromosome -: Unknown

Supplementary Table 3. Frequency of the *HWE1* genotype in the transgenic populations.

Transgene	T1 line #	*HWE1* genotype[a] II	IJ	JJ	Total	χ^2 (1:2:1)
G20324 (Cycline-A1)	T1-3	4 (0)	12 (2)	5 (0)	20 (2)	0.52[ns]
	T1-6	3 (1)	16 (3)	5 (0)	24 (4)	3.00[ns]
	T1-12	2 (0)	6 (0)	3 (0)	11 (0)	0.27[ns]
G20310 (EAF6)	T1-3	8 (0)	13 (2)	6 (1)	27 (3)	0.33[ns]
	T1-27	12 (0)	40 (5)	12 (5)	64 (10)	4.00[ns]
	T1-23	17 (3)	23 (4)	17 (3)	57 (10)	2.12[ns]
	T1-6	9 (0)	21 (4)	17 (3)	47 (7)	3.26[ns]
	T1-4	5 (0)	9 (2)	6 (0)	20 (2)	0.30[ns]
	T2-3-16	11(2)	18 (3)	12 (2)	41 (7)	0.67[ns]

a: T1 and T2 seedlings were genotyped with the DNA marker *12c106* linked to the *hwe1* gene. II: IR24 homozygote, IJ: heterozygote, JJ: Asominori homozygote. The seedlings homozygous for the IR24 allele (*hwe1/hwe1*) were transplanted and used for phenotyping of individuals in Supplementary Table S4.

b: Number of the individuals without the transgene was shown in parentheses.

ns: not significant at P=0.05

Supplementary Table 4. Phenotype of the transgenic plants that harbor G20324 or G20310.

		No. of plants				
		Normal		Weak		
Transgene	Line #	+	−	+	−	Total
G20324 (Cycline-A1)	T1-3	0	0	4	0	4
	T1-6	0	0	2	1	3
	T1-12	0	0	2	0	2
G20310 (EAF6)	T1-3	7	0	0	0	7
	T1-27	11	0	0	0	11
	T1-23	14	0	0	3	17
	T1-6	7	0	0	0	7
	T1-4	4	0	0	0	4
	T2-3-16	9	0	0	2	11

#See Supplementary Table 3 for population description.
+: the individuals with the transgene, −: without the transgene.
